# Superhydrophobic Cell‐Repellent Microstructures: Plastron‐Mediated Inhibition of A549 Epithelial Cell Adhesion

**DOI:** 10.1002/smll.202506022

**Published:** 2025-08-19

**Authors:** Mohammad Awashra, Ville Jokinen

**Affiliations:** ^1^ School of Chemical Engineering Department of Chemistry and Materials Science Aalto University Tietotie 3 Espoo 02150 Finland

**Keywords:** air plastron, biointerface, cell adhesion, microstructures, physical repellency, superhydrophobic

## Abstract

Control of cell adhesion is essential for biomedical devices, biosensors, and anti‐fouling coatings. Here, adhesion of A549 epithelial cells is systematically evaluated on silicon substrates with tunable wettability (superhydrophilic to superhydrophobic) and topography (smooth, nanostructured, and micropillared). Superhydrophobic surfaces stabilize a trapped air plastron that minimizes solid–liquid contact, enabling *plastron‐mediated physical repellency of cells*. The most cell‐repellent surface, composed of 5 µm micropillars with a 7.4% solid–liquid contact fraction, reduced cell density by ≈83% versus a smooth hydrophobic control and ≈95% versus a hydrophilic control at 4 h, and by ≈90% and ≈93%, respectively, after 24 h of incubation, corresponding to an approximate tenfold decrease in cell adhesion. Micropillar arrays outperform nanostructures in resisting cell attachment, owing to large air‐filled gaps exceeding 10 µm that *physically* prevent cell adhesion. A trade‐off is observed: lower solid‐fraction micropillars provide greater short‐term repellency but lose the plastron over time, enabling delayed fouling, whereas higher‐fraction structures preserve the air layer beyond 72 h but are initially less cell‐repellent due to higher effective cell contact area and smaller air gaps. These results establish that optimized microscale superhydrophobic textures achieve superior and time‐dependent bio‐repellency and introduce a rational design strategy for non‐fouling materials.

## Graphical abstract

Engineered superhydrophobic micropillars reduced A549 cell adhesion tenfold after 1 day of incubation via a plastron‐mediated physical repellency mechanism, independent of cell‐surface biochemical interactions. Microstructured surfaces outperformed nanostructures, providing the first experimental evidence of microscale superiority. Optimization of pillar size and solid fraction revealed a trade‐off between effective cell contact area and plastron stability, informing antifouling surface design.

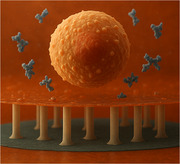

## 1 Introduction

Superhydrophobic (SHB) surfaces have emerged as promising candidates for a wide range of applications due to their ability to repel liquids, resist contamination through self‐cleaning, and reduce drag.^[^ ^1^, ^2^, ^3^, ^4^, ^5^, ^6^ ^]^ Their capacity to repel biofluids, proteins, sugars, blood, bacteria, and mammalian cells^[^ ^7^, ^8^, ^9^, ^10^, ^11^, ^12^, ^13^ ^]^ has enabled biomedical innovations such as micropatterned arrays for nucleic acid detection,^[^ ^14^ ^]^ single‐cell trapping platforms,^[^ ^15^ ^]^ drag‐reducing and blood‐repellent coatings,^[^ ^16^, ^17^, ^18^, ^19^ ^]^ antibacterial coatings,^[^ ^20^, ^21^ ^]^ and spheroid culture scaffolds.^[^ ^22^ ^]^

A defining feature of superhydrophobic surfaces is the formation of air plastron, a thin layer of air retained at the solid–liquid interface.^[^ ^23^, ^24^, ^25^ ^]^ This layer physically isolates the substrate from the surrounding medium, minimizing solid–liquid contact and thus reducing biomolecule and cell adhesion.^[^ ^12^, ^26^, ^27^, ^28^, ^29^ ^]^ However, plastron lifetimes vary widely, from seconds to weeks, depending on the surface chemistry, micro‐/nano‐structural topography, and environmental conditions.^[^ ^12^, ^30^, ^31^, ^32^, ^33^, ^34^, ^35^, ^36^, ^37^, ^38^, ^39^, ^40^, ^41^ ^]^ Sustaining the plastron over extended periods remains a key challenge, particularly upon immersion in complex biological fluids.

Cell adhesion is a multistep process initiated by protein adsorption onto the substrate, forming a conditioning layer that mediates integrin–ligand interactions and governs subsequent attachment, spreading, and proliferation.^[^ ^42^ ^]^ Adsorbed extracellular matrix proteins such as fibronectin, laminin, and collagen are key ligands for integrins, triggering signaling pathways that promote focal adhesion assembly and actin cytoskeleton organization.^[^ ^43^ ^]^ This process is highly sensitive to substrate properties including wettability, topography, surface charge, and chemical composition, which influence protein conformation and kinetics.^[^ ^9^, ^28^, ^42^, ^44^, ^45^, ^46^, ^47^, ^48^ ^]^ Atomic vacancies and electrochemical doping have also been shown to influence adhesion, likely by altering surface energy and molecular interactions.^[^ ^49^, ^50^, ^51^ ^]^

Maximal protein and cell adhesion typically occur on surfaces with moderate wettability (contact angles ≈40–70°).^[^ ^28^, ^42^, ^52^, ^53^, ^54^, ^55^ ^]^ The behavior of cells on superhydrophobic surfaces, however, remains under debate.^[^ ^56^ ^]^ While many studies report reduced cell adhesion due to minimized contact area and altered protein conformation,^[^ ^9^, ^27^, ^28^, ^29^, ^42^, ^45^, ^46^, ^47^, ^48^, ^49^, ^50^, ^54^, ^57^, ^58^, ^59^, ^60^, ^61^, ^62^, ^63^, ^64^, ^65^, ^66^, ^67^, ^68^, ^69^, ^70^, ^71^, ^72^, ^73^, ^74^, ^75^, ^76^, ^77^, ^78^, ^79^, ^80^, ^81^, ^82^, ^83^, ^84^, ^85^, ^86^, ^87^, ^88^, ^89^, ^90^, ^91^, ^92^, ^93^, ^94^, ^95^, ^96^, ^97^, ^98^ ^]^ others observe enhanced adhesion under certain surface chemistries or morphologies.^[^ ^99^, ^100^, ^101^, ^102^, ^103^, ^104^, ^105^, ^106^, ^107^, ^108^ ^]^ A wettability gradient surface using carbon nanoparticles was shown to guide NIH/3T3 fibroblast adhesion, with cells adhering preferentially to superhydrophilic (SHL) and Wenzel state regions (i.e., regions of full wetting) and being repelled from Cassie–Baxter regions (i.e., regions with retained air pockets).^[^ ^46^ ^]^

While extensive research has been devoted to nanostructured superhydrophobic surfaces, microscale architectures have been less systematically studied for their role in submerged cell‐repellency. Nanostructures impact cell adhesion by altering protein adsorption and integrin clustering, which govern focal adhesion formation and strength.^[^ ^109^, ^110^ ^]^ Microstructures, on the other hand, primarily influence cell alignment, orientation, and spreading by providing physical cues.^[^ ^47^ ^]^ Micropatterned silicon stripes enabled over 95% alignment fidelity of cells,^[^ ^57^ ^]^ and PDMS micropillars, despite lacking plastrons, guided cancer cell adhesion and migration via geometrical cues.^[^ ^111^ ^]^

Although microscale structures are widely used to study cell mechanics and morphology, few studies have examined their ability repel cells by sustaining air plastron when submerged. Achieving Cassie–Baxter wetting state at the microscale, though challenging, is attainable through appropriate roughness and aspect ratio design. Expanded discussion on the microscale‐only research gap is in Section S0 (Supporting Information). Our previous work demonstrated that microscale features with optimized spacing and aspect ratios can stabilize air plastron and improve biofluid repellency.^[^ ^12^ ^]^ Wang et al.^[^ ^112^ ^]^ fabricated chemically heterogeneous SU‐8 micropillars (30–100 µm) and showed that cells adhered only to the hydrophilic (HL) pillar tops, while being repelled from the hydrophobic (HB) pillar sides and trenches due to the air plastron barrier. This behavior exemplifies the so‐called “*Salvinia* effect”. A recent study by Yu et al.^[^ ^113^ ^]^ used the same wettability effect but to repel blood instead of cell trapping. The authors demonstrated that chemically heterogeneous nanostructured candle soot templated silica surfaces incorporating hydrophilic, protein‐repellent polyethylene glycol (PEG)‐coated tops are able to trap plastron by mimicking the *Salvinia* effect and can drastically prolong Cassie–Baxter stability in blood due to the pinning effect at the hydrophilic sites. This engineered wetting contrast extended the blood‐repellent (superhemophobicity) time over tenfold compared to conventional homogeneous superhydrophobic surfaces and achieved sustained hemocompatibility for more than 55 h in vivo.

Over recent decades, hydrophilic polymer coatings such as PEG,^[^ ^114^ ^]^ zwitterionic materials,^[^ ^115^, ^116^ ^]^ and hydrogels,^[^ ^117^ ^]^ have been widely applied to biofluid‐contacting surfaces to reduce protein adsorption and cell adhesion.^[^ ^7^, ^16^ ^]^ However, PEG‐based coatings are known to be thermally sensitive, with dynamic viscosity and density declining with temperature affecting their antifouling performance in physiological conditions.^[^ ^118^ ^]^ Hydrogel coatings are often limited to simple geometries,^[^ ^117^ ^]^ and grafted biomacromolecules like heparin, while effective short‐term, suffer from degradation, uneven coverage, and nonspecific fouling in biological media.^[^ ^7^, ^16^, ^119^ ^]^ Liquid‐infused surfaces, including slippery liquid‐infused porous surfaces (SLIPS),^[^ ^120^ ^]^ provide low‐friction, omniphobic barriers,^[^ ^121^, ^122^ ^]^ but lose functionality over time due to lubricant depletion and substrate exposure. While some coatings, such as Lipidure, offer advantages like low cost and biocompatibility,^[^ ^123^ ^]^ their performance is typically short‐lived in dynamic biological environments. These limitations highlight the need for antifouling strategies that rely on physical (i.e., plastron in superhydrophobic surfaces) rather than purely chemical mechanisms.

In this study, we systematically investigate how microscale‐only superhydrophobic surfaces with varied pillar sizes, solid fractions (i.e., percentage of substrate area that is solid (pillar tops) vs air gaps), and roughness affect A549 cell adhesion under submerged conditions. We benchmark these against hydrophilic and hydrophobic smooth, nanostructured, and microstructured surfaces. Our results demonstrate that 5 µm‐micropillars with a 7.4% solid fraction exhibit the strongest cell‐repellency, surpassing nanostructures. We propose that the key to micropillar cell‐repellency is to introduce air gaps that exceed cell dimensions, thus inhibiting focal adhesion. By resolving how geometry, plastron lifetime, and surface wetting together regulate adhesion, this study advances the understanding of functional superhydrophobic surface design and identifies microscale architectures as a superior, time‐sensitive strategy for biomedical antifouling interfaces.

## 2 Results and Discussion

### 2.1 Surface Morphology and Wettability

**Figure 1:**
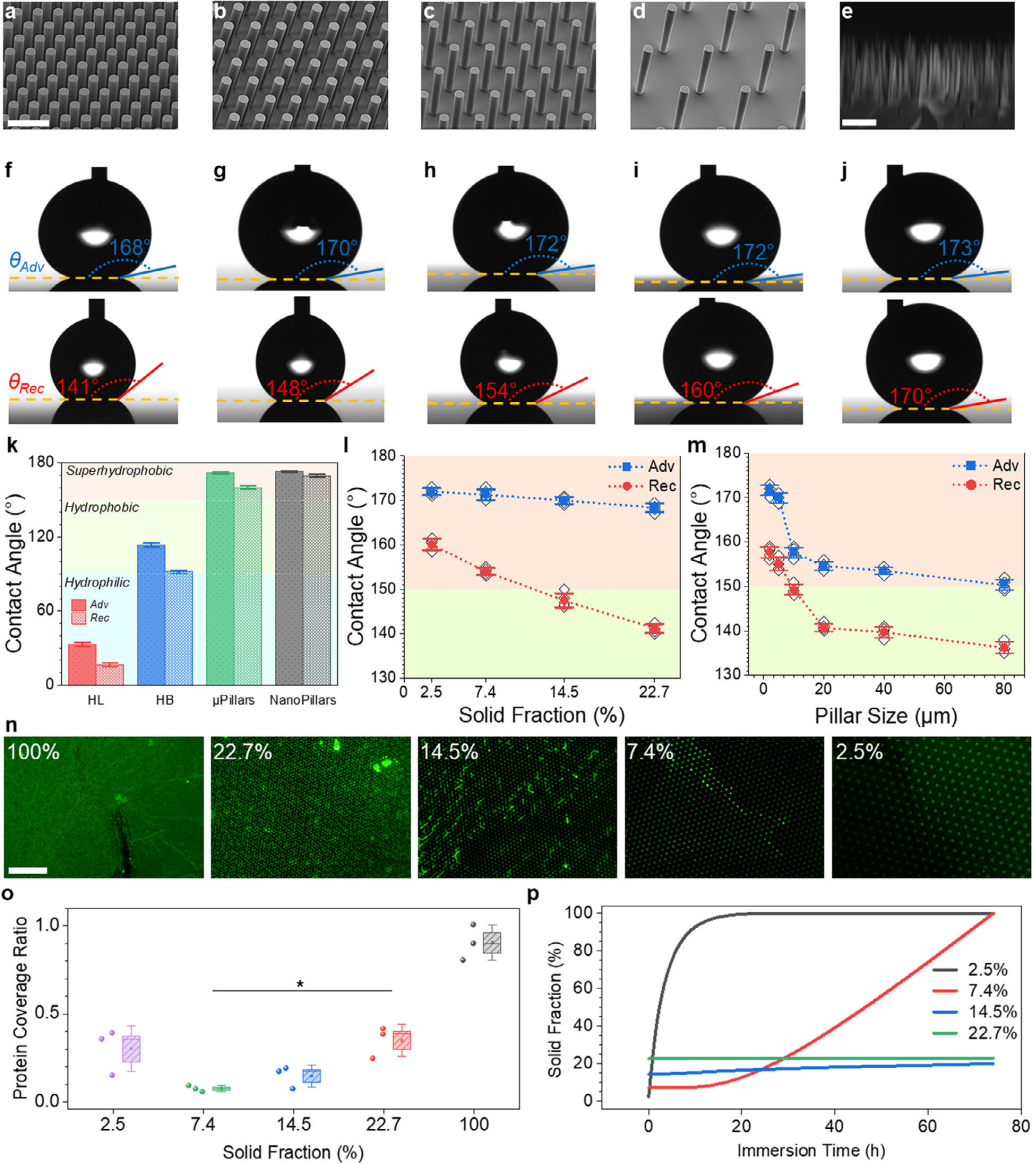
Structural and wetting characterization of the surfaces. a–d) Tilted SEM images of 5 µm silicon micropillars with solid fractions of 22.7%, 14.5%, 7.4%, and 2.5%, respectively. Scale bar: 20 µm. e) Cross‐sectional SEM image of silicon nanopillars. Scale bar: 1 µm. f–j) Water contact angle images displaying advancing (θ_Adv_, top) and receding (θ_Rec_, bottom) contact angles for the corresponding surfaces above each panel. Needle diameter: 311 µm. k) Contact angles for smooth hydrophilic, hydrophobic, micropillared, and nanopillared surfaces. l,m) Influence of solid fraction and pillar size on contact angles. In k–m, data are presented as mean ± standard deviation (SD), with *n* = 3 per group. n) Fluorescence microscopy images of protein adsorption on hydrophobic control (100%) and 5 µm pillars with varying solid fraction. Scale bar: 200 µm. o) Quantification of protein coverage as a function of solid fraction. Data are presented as box plots showing the mean (square dot), with boxes representing the standard error, whiskers indicating standard deviation, and horizontal lines showing the median. Individual data points correspond to independent fluorescence imaging fields. Statistical significance was assessed using one‐way analysis of variance (ANOVA) with Tukey's post hoc test (*p* < 0.05). *n* = 3 per group. ^*^ *p* < 0.05, (the 100% group showed *p* < 0.0001 versus all other groups). p) Time‐dependent solid–liquid area fraction during immersion in cell‐containing culture medium, indicating plastron stability.

We fabricated silicon micropillar arrays with tunable pillar sizes (2–50 µm) and solid fractions (22.7%, 14.5%, 7.4%, and 2.5%) using photolithography and BOSCH deep reactive ion etching (DRIE), followed by a fluoropolymer (FP) coating to render the surfaces superhydrophobic (Figure S1, Supporting Information). Scanning electron microscopy (SEM) confirmed the precise fabrication and reproducibility of pillar geometry (**Figure** **1**a–e; Figure S2, Supporting Information). Figure 1f–m shows that different surface morphologies and coatings produced distinct water contact angles, ranging from hydrophilic to superhydrophobic regimes depending on topography and surface chemistry. Superhydrophobic surfaces exhibited advancing contact angles between 168° and 173° and receding angles between 141° and 170° (Figure 1f–j; Figure S3, Supporting Information). The nanopillared surface exhibited the highest contact angles, emphasizing the role of surface roughness in water repellency (Figure 1k). Lower solid fractions showed higher receding contact angles and consequently decreased contact angle hysteresis (CAH) (Figure 1l). Larger pillars promoted decreased contact angles as shown in Figure 1m and Figure S3 (Supporting Information). It is important to note that not all micropillared surfaces in this study met the classical definition of superhydrophobicity, which typically requires a CAH below 10°. The low solid fraction micropillars exhibited low CAH and advancing contact angles exceeding 170°, consistent with superhydrophobic behavior. In contrast, high solid fraction micropillars showed higher CAH and thus do not qualify as superhydrophobic. Nevertheless, our analysis emphasizes functional performance (air plastron retention and effective cell repellency) rather than relying solely on strict wetting thresholds. Further discussion is in Section S1 (Supporting Information).

### 2.2 Plastron Stability and Protein Repellency

The superhydrophobic samples were submerged in water and instantly trapped a reflective air film (plastron) visible as a mirror‐like underwater sheen (Figure S4, Supporting Information), confirming the Cassie–Baxter state. Bright‐field optical images of the corresponding micropillar arrays reveal well‐defined structures with varying pillar diameters and solid fractions, allowing controlled modulation of wettability and air retention. Since protein adsorption mediates subsequent cell attachment, we studied protein adsorption on the surfaces. Fluorescence imaging of fluorescein isothiocyanate‐conjugated bovine serum albumin (FITC–BSA) revealed that the smooth hydrophobic control (100% solid fraction) became uniformly coated with protein, whereas all four microtextured surfaces showed significantly less adsorption (Figure 1n). The 7.4% solid fraction surface exhibited the least protein coverage, consistent with maximal air fraction and minimal solid contact. Quantitatively, protein coverage on 5 µm‐pillar surfaces decreased by ≈90% when solid fraction was reduced from 100% to 7.4% (Figure 1o). Importantly, protein fouling depended not just on initial Cassie–Baxter state but on its stability. The ultralow 2.5% fraction surface, despite its largest initial air fraction, showed higher protein adsorption than the 7.4% surface. We attribute this to a partial loss of plastron on the 2.5% surface during the 4 h incubation (Figure 1p). Further discussion is in Section S2 (Supporting Information).

### 2.3 Wettability and Roughness Effects on Cell Adhesion

**Figure 2:**
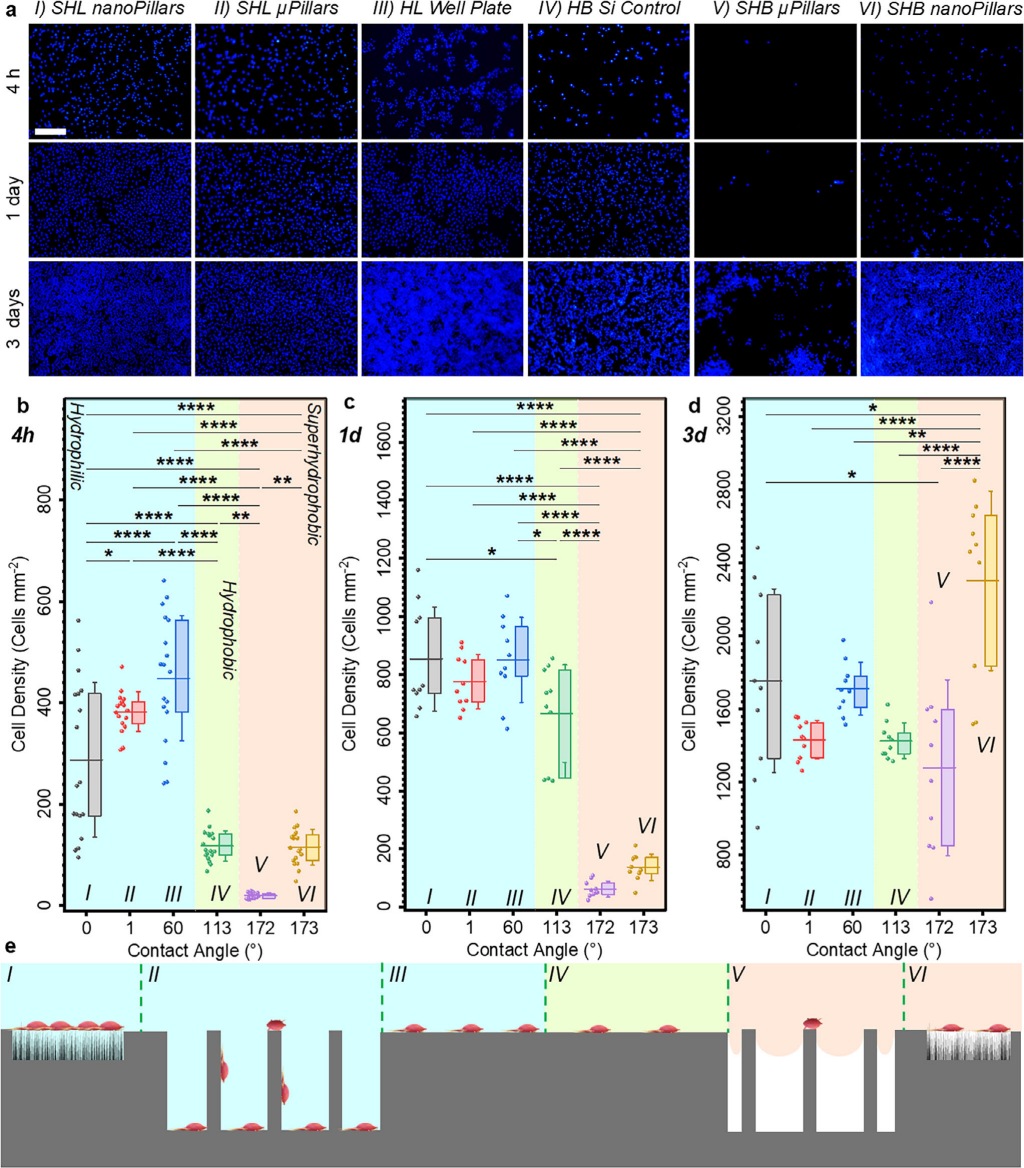
Wettability and roughness effects on cell adhesion over time. a) Low magnification fluorescence microscopy images showing A549 cell adhesion on superhydrophilic, hydrophilic, hydrophobic, and superhydrophobic surfaces after 4 h, 1 day, and 3 days of incubation. Scale bar: 200 µm. b–d) Quantification of cell density as a function of surface advancing contact angle at each timepoint, illustrating how wettability and roughness influence cell adhesion. Data are presented as box plots showing the interquartile range (25^th^–75^th^ percentile), with horizontal lines indicating the mean and whiskers representing standard deviation. Individual data points (shown as dots) correspond to independent imaging fields collected from multiple surface replicates. Statistical significance was assessed using one‐way ANOVA with Tukey's post hoc test (*p* < 0.05). *n* = 6 per group for 4 h and *n* = 3 per group for 1 and 3 days. ^*^ *p* < 0.05, ^**^ *p* < 0.01, ^***^ *p* < 0.001, ^****^ *p* < 0.0001. e) Schematic representation of wetting states across the six surfaces.

We evaluated how the wettability differences, measured by contact angles, translate to mammalian cell adhesion using A549 epithelial cells. Six surface types were examined (**I**: uncoated superhydrophilic silicon nanopillars; **II**: uncoated superhydrophilic silicon micropillars; **III**: hydrophilic tissue‐culture plastic control; **IV**: smooth hydrophobic silicon control; **V**: superhydrophobic silicon micropillars; **VI**: superhydrophobic silicon nanopillars. After 4 h incubation (initial attachment phase), cell density correlated strongly with surface wetting state (**Figure** **2**a,b). Hydrophilic surfaces (surfaces I–III) supported abundant cell attachment (hundreds of cells per mm^2^), reflecting the known preference of cells for moderate‐to‐high surface wettability (due to rapid protein conditioning).^[^ ^28^, ^42^, ^52^, ^53^, ^54^, ^55^ ^]^ In contrast, hydrophobic surfaces (surfaces IV–VI) showed reduced cell adhesion. The superhydrophobic microtextured surface (surface V, θ ≈172°) was profoundly cell‐repellent, where it showed an ≈83% reduction in cell density compared to the smooth hydrophobic control (surface IV, θ ≈113°) and a ≈95% reduction compared to hydrophilic surfaces. This surface consists of 5 µm pillars with 7.4% solid fraction. Quantitatively, only ≈20 ± 5 cells mm^−2^ adhered to the superhydrophobic micropillars at 4 h, versus ≈118 ± 30 cells mm^−2^ on the hydrophobic control and ≈450 ± 124 cells mm^−2^ on the hydrophilic control. This dramatic short‐term repellency is attributed to the plastron in the Cassie–Baxter state, which physically limits direct cell contact with the solid substrate by forming a virtual barrier (Figure 2e).

### 2.4 Microscale Outperforms Nanoscale in Cell‐Repellency

Although the nanopillars (surface VI, θ ≈ 173°) reduced cell adhesion compared to the hydrophilic control, its cell density matched that of the smooth hydrophobic control and was six times higher than that of the micropillars (Figure 2b). This finding challenges the common assumption that nanoscale roughness is superior for anti‐fouling. *Instead, our results provide the first experimental evidence that well‐designed microstructured superhydrophobic surfaces outperform their nanostructured counterparts in cell‐repellency*. We propose two complementary reasons for these findings: 1) Geometric air gaps: The microscale pillars create voids 10–50 µm across (at 7.4%–2.5% solid fractions) that exceed typical cell dimensions, making it physically difficult for a cell (≈10–30 µm) to reach the substrate or bridge between multiple pillars. Cells on the 7.4% surface remained mostly suspended between pillar tops with minimal spreading (Figure 2e), as the wide air gaps prevented integrin engagement on more than one pillar at a time. In contrast, the nanopillar surface presents a forest of tips on the scale of 10–100 nm, which, although minimizing total contact area, offers innumerable small anchoring points within a single cell's footprint. These nanoscale contact points can promote filopodial attachment and integrin clustering at the cell periphery, allowing cells to eventually secure themselves despite the reduced area. 2) Protein conformation: Adsorbed serum proteins on nanostructures experience extreme curvature at the nanoscale, which can preserve their native conformation and bioactivity (exposing more cell‐adhesive motifs).^[^ ^11^, ^124^ ^]^ On microscale textures, adsorbed proteins likely undergo greater denaturation, reducing their bioactivity compared to intact proteins on nanopillars that may better support cell attachment. These factors together explain why the superhydrophobic nanopillar surface, despite its higher contact angles, had more early cell attachment than the superhydrophobic micropillars. Previous findings showed that microscale roughness composed of ≈1–2 µm features showed poorer cell‐repellency than nanostructures when the microscale air gaps were too small (<1 µm) to prevent cell bridging over multiple pillars.^[^ ^59^ ^]^ In our case, the wide micropillar gaps are sufficiently large to hinder cell bridging, making the Cassie–Baxter state micropillars the most cell‐repellent at the early time point (4 h).

### 2.5 Plastron Stability Controls Long‐Term Cell Adhesion

Generally, prolonged incubation resulted in increased cell adhesion due to delayed focal adhesion formation and subsequent cell proliferation. After 1 day, hydrophilic and hydrophobic control surfaces showed increased cell coverage due to continued attachment and proliferation (Figure 2c). In contrast, superhydrophobic micro and nanopillar surfaces, which sustained Cassie–Baxter state throughout the 24 h incubation, continued to exhibit low cell densities comparable to their 4 h values. Micropillars exhibited a tenfold reduction in cell adhesion compared to both control surfaces. The few cells present on Cassie–Baxter state superhydrophobic surfaces at 1 day often formed loosely attached clusters or spheroids rather than spreading into a monolayer. The limited adhesion area caused by the plastron may have restricted cell spreading and proliferation, potentially delaying or arresting cell cycle progression.^[^ ^112^ ^]^ A dramatic increase in biofouling occurred once the plastron collapsed on the superhydrophobic surfaces. By day 3 of immersion, both superhydrophobic micropillared and nanopillared surfaces underwent at least partial wetting transitions to the Wenzel state, as evidenced by loss of the mirror‐like reflection and a surge in cell attachment (Figure 2d). Further discussion on Figure 2 and Figures S6–S21 is in Section S3 (Supporting Information). It is worthy to mention that cell repellency in our study arises from the plastron‐mediated physical barrier at the solid–liquid interface, not cytotoxicity. This is evidenced by preserved cell morphology and proliferation on smooth hydrophobic controls, confirmed by 4′,6‐diamidino‐2‐phenylindole (DAPI)/phalloidin imaging showing intact nuclei and cytoskeleton and normal proliferation rate over 72 h (Figures S16 and S17, Supporting Information). Furthermore, A549 cells adhered and spread extensively on micropillared surface in the Wenzel state after 24 h as shown in the SEM image in Figure S22 (Supporting Information).

### 2.6 Micropillar Solid Fraction Modulates Cell Adhesion Over Time

**Figure 3:**
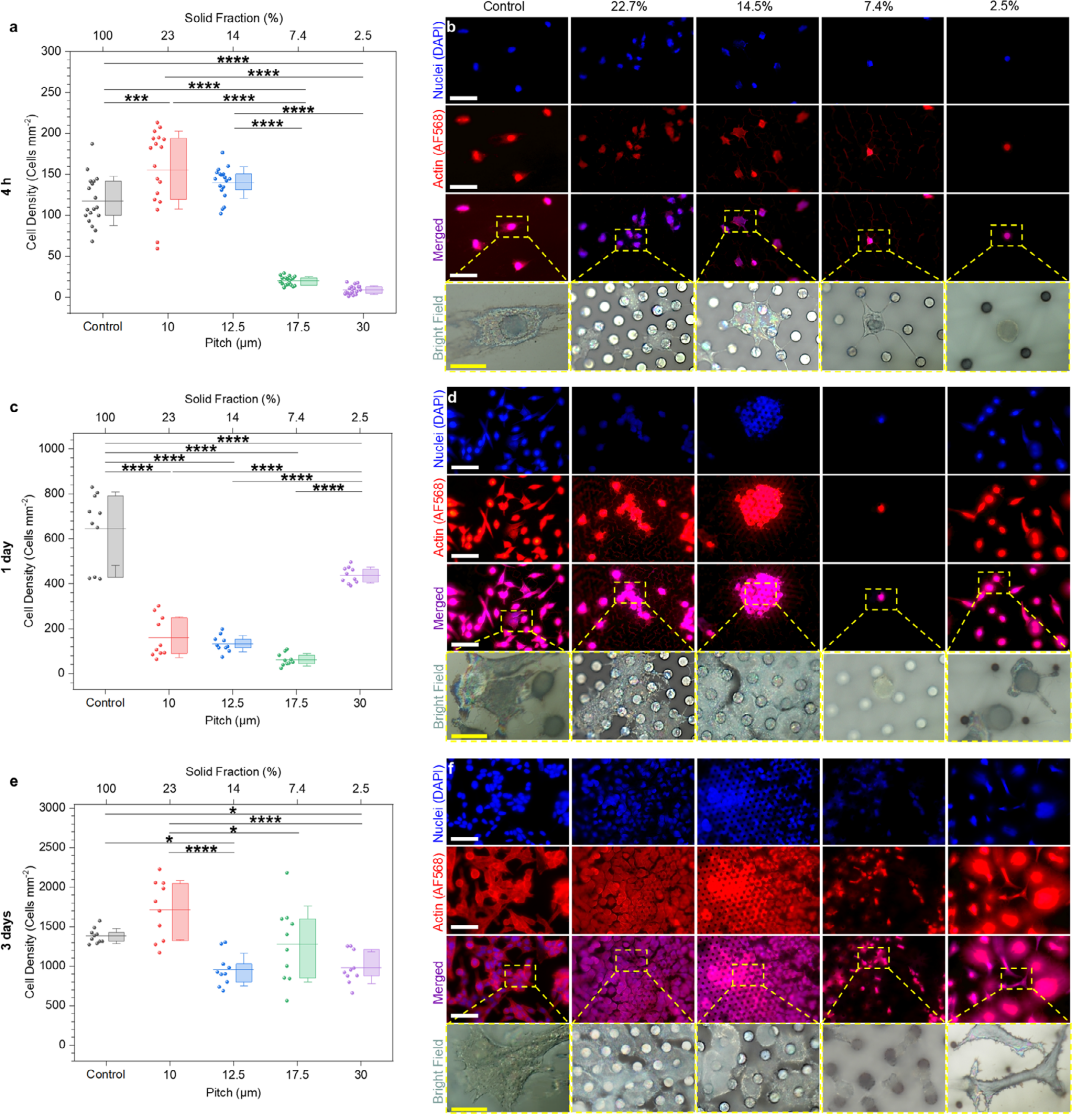
Micropillar solid fraction governs temporal evolution of cell adhesion. a,c,e) Quantification of cell density on 5 µm micropillars with varying solid fractions (22.7%, 14.5%, 7.4%, and 2.5%) after 4 h, 1 day, and 3 days of incubation, respectively. Data are presented as box plots showing the interquartile range (25^th^–75^th^ percentile), with horizontal lines indicating the mean and whiskers representing standard deviation. Individual data points (shown as dots) correspond to independent imaging fields collected from multiple surface replicates. Statistical significance was assessed using one‐way ANOVA with Tukey's post hoc test (*p* < 0.05). *n* = 6 per group for 4 h and *n* = 3 per group for 1 and 3 days. ^*^ *p* < 0.05, ^**^ *p* < 0.01, ^***^ *p* < 0.001, ^****^ *p* < 0.0001. b,d,f) High‐magnification fluorescence and bright‐field microscopy images showing representative cell morphologies on each surface at the corresponding timepoints. Cells were stained for nuclei (DAPI, blue) and actin cytoskeleton (AF568, red). Scale bars: 50 µm (fluorescence), 20 µm (bright field).

To investigate how microstructure geometry influences the time‐dependent cell repellency of the micropillars, we systematically evaluated A549 cell adhesion across micropillar arrays with varying solid fractions (2.5–22.7%) over a 3‐day period. At 4 h incubation, A549 cell attachment was strongly dependent on the micropillar solid fraction. Surfaces with higher solid fractions (14.5% and 22.7%) showed significantly greater cell adhesion than those with lower fractions (7.4% and 2.5%), despite all four surfaces being in the Cassie–Baxter state with an entrapped air plastron. Quantitatively, cell density dropped sharply, by approximately tenfold, as solid fraction decreased from 14.5% to 7.4% (*p* < 0.0001) (**Figure** **3**a). Corresponding fluorescence images offered mechanistic insight: on high‐solid‐fraction surfaces cells were well‐spread with extensive actin networks, whereas on the low‐solid‐fraction surfaces cells remained mostly rounded with minimal cytoskeletal development (Figure 3b). These observations confirm that large air gaps at low solid fraction prevent cell adhesion, while smaller inter‐pillar gaps at higher fractions allow cells to bridge over multiple pillars and adhere more readily. After 1 day, cell coverage on the high‐ and medium‐solid‐fraction micropillars (22.7%, 14.5%, and 7.4%) remained largely unchanged from the 4 h state (Figure 3c). This suggests that the air plastron persisted on these surfaces, continuing to physically hinder firm cell–surface contacts and subsequent cell proliferation. Indeed, cells on these plastron‐retaining surfaces often clustered in spheroid‐like aggregates rather than forming spread monolayers (Figure 3d), consistent with limited focal adhesion points. By contrast, the lowest solid fraction surface (2.5%) (which had shown almost no initial cell adhesion) exhibited a dramatic rise in cell density by day 1, surpassing all other samples. This rapid fouling is attributed to an early loss of the air plastron of this surface (within ≈4 h of immersion, Figure 1p), triggering a Cassie‐to‐Wenzel wetting transition that exposed the total area of the substrate and enabled extensive cell attachment and spreading. SEM images directly capture this transition, showing that cells on the 2.5% pillars could reach the underlying substrate once the air layer collapsed (Figure S22, Supporting Information). By day 3 of incubation, a clear increase in cell adhesion was observed on all the micropillar surfaces as the remaining plastrons eventually dissipated (Figure 3e). Still, important differences emerged in the extent of fouling. The intermediate solid fraction (14.5%) maintained the lowest cell coverage, suggesting it sustained an effective plastron the longest; its relatively large inter‐pillar gaps also limited cell attachment compared to the more closely spaced 22.7% surface. In contrast, the surfaces that were most cell‐repellent initially (7.4% and especially 2.5%) became heavily fouled once their air interface was lost, with cells spreading freely across previously non‐wetted regions (Figure 3f). These findings highlight an inherent design trade‐off between plastron stability and short‐term cell‐repellency. Lower solid fractions maximize initial cell‐repellency through greater air plastron coverage but are more susceptible to early collapse.^[^ ^12^ ^]^ In contrast, higher solid fractions better preserve the plastron over time but increase the early available adhesion area. Although the differences were modest, the 7.4% design showed the strongest short‐term repellency (up to 1 day). Further discussion is in Section S4 (Supporting Information).

### 2.7 Pillar Size Regulates Cell Adhesion

**Figure 4:**
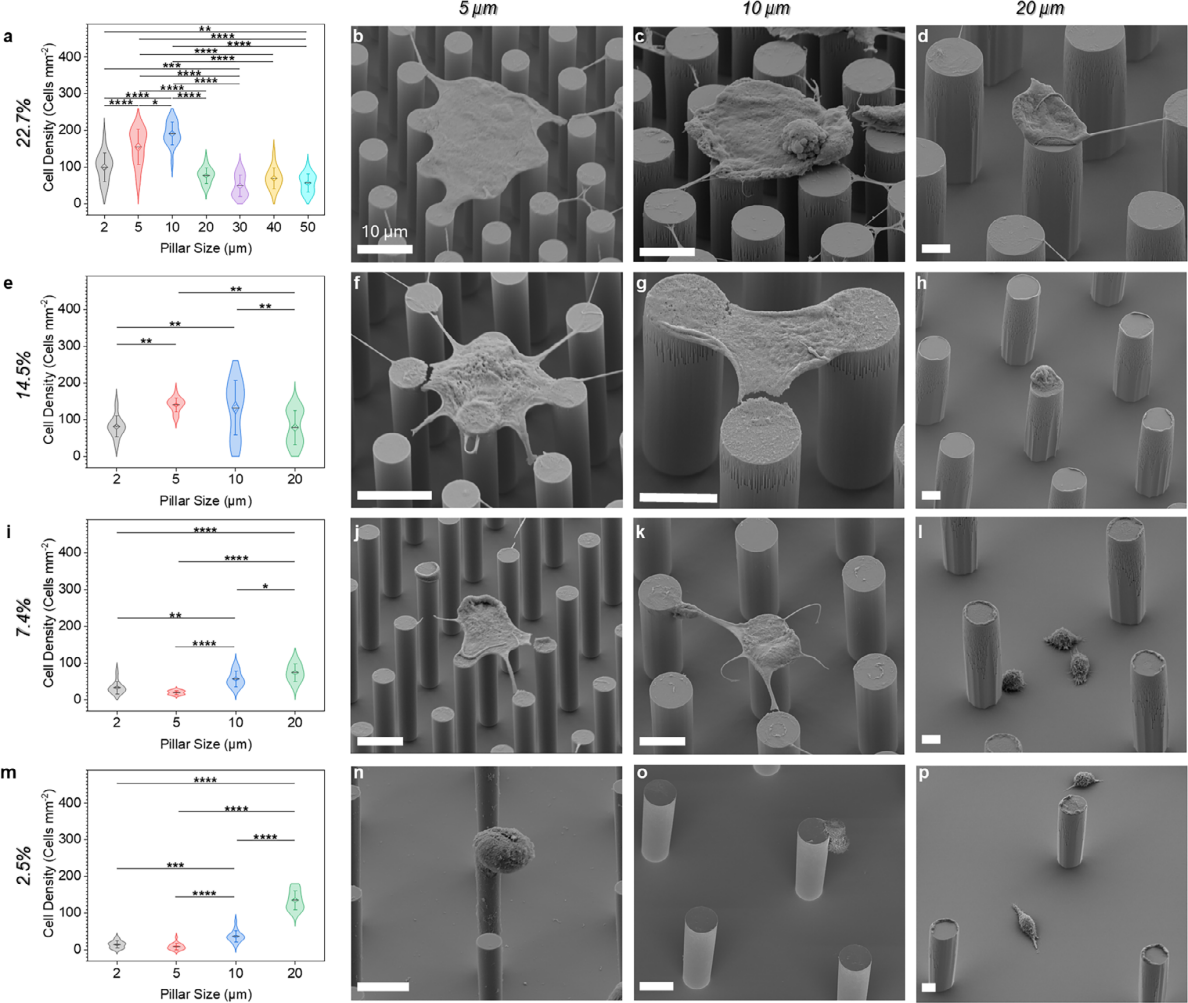
Influence of pillar size on cell‐repellency. A549 cell adhesion was quantified after 4 h of incubation on micropillar arrays with varying pillar diameters (2, 5, 10, and 20 µm) at four solid fractions: a) 22.7%, e) 14.5%, i) 7.4%, and m) 2.5%. Violin plots display the kernel‐smoothed distribution of cell density (cells mm^−2^). Overlaid boxes represent the standard error, with the horizontal line indicating the mean and whiskers denoting standard deviation. Statistical significance was assessed using one‐way ANOVA with Tukey's post hoc test (*p* < 0.05). *n* = 6 per group. ^*^ *p* < 0.05, ^**^ *p* < 0.01, ^***^ *p* < 0.001, ^****^ *p* < 0.0001. b–d, f–h, j–l, n–p) SEM images show representative cell morphologies on the corresponding surfaces organized in a matrix format by solid fraction (rows) and pillar size (columns). Scale bars: 10 µm.

To decouple the influence of pillar size from solid fraction, we examined A549 cell adhesion on micropillars with diameters ranging from 2 to 50 µm across four solid fractions after 4 h of incubation. The results reveal two distinct governing mechanisms: mechanical bridging at high solid fractions, and wetting transitions at low solid fractions. At 22.7% and 14.5% solid fractions, all pillar sizes maintained Cassie–Baxter state. Surfaces with small pillars (2–10 µm) supported significantly higher cell densities than those with larger pillars (**Figure** **4**a–h). Supporting fluorescence images (Figures S23 and S24, Supporting Information) showed dense cell layers on the smallest pillars, while 20–50 µm pillars had visibly fewer adherent cells. SEM images confirm that small pillars facilitated filopodia bridging across adjacent tops (Figure 4b,c,f,g), whereas cells on 20 µm pillars were largely confined to a single pillar's top (Figure 4d,h), as also seen in Figures S25 and S26 (Supporting Information). These trends occurred despite all samples retaining stable plastrons during this time, confirming that interpillar spacing, not wetting, is the main determinant for adhesion for high‐solid‐fraction surfaces.

In contrast, at 7.4% and 2.5% solid fractions, the pillar diameter dictated plastron stability, thereby controlling the wetting state and adhesion outcome. Figure S27 (Supporting Information) shows that 2–10 µm pillars at 7.4% solid fraction maintained the cell‐repellent Cassie–Baxter state (Figure 4i–k), while 20 µm pillars transitioned to the fully wetting Wenzel state and exhibited significantly more cell coverage (Figure 4l; Figure S28, Supporting Information). The same pattern was more pronounced at 2.5% solid fraction: Figures S29 and S30 (Supporting Information) reveal that 20 µm pillars, due to wide 100 µm spacing, failed to retain the air plastron and rapidly fouled, whereas 2–10 µm pillars still repelled cells due to plastron retention (Figure 4m–p). These results indicate that larger pillars at low solid fractions destabilize the plastron, allowing liquid intrusion and cell access to the substrate. Thus, pillar size modulates cell adhesion via cell bridging at high solid fractions and via wetting transitions at low solid fractions. The optimal repellency is achieved on small‐diameter, low‐solid‐fraction micropillars capable of maintaining Cassie–Baxter wetting while presenting air gaps that exceed cell dimensions, thereby preventing both mechanical anchoring and wetting‐mediated attachment. Longer incubation times (1 and 3 days) showed convergence in adhesion across geometries due to wetting transitions and proliferation, as detailed in Section S5 and Figures S31–S54 (Supporting Information). The effect of pillar height on plastron stability is shortly discussed in Section S6 (Supporting Information).

## 3 Conclusion

Our findings establish that appropriately engineered microscale superhydrophobic surfaces can dramatically repel mammalian cells via plastron‐mediated physical repellency mechanism, surpassing the performance of conventional smooth or nanostructured interfaces. We identified that micropillars with 5 µm diameter and 7.4% solid fraction achieved an ≈95% reduction in adhered A549 cell numbers after 4 h compared to the hydrophilic control. These low‐solid‐fraction microscale designs maintained extremely low cell adhesion for at least one day of full immersion, demonstrating that high levels of cell repellency can extend beyond the hour timescale typically reported for anti‐fouling surfaces. This enhanced performance is attributed to the large air gaps in microstructured surfaces, which restrict focal adhesion formation and reduce effective cell‐surface contact. Even a 1–3 day delay in cell fouling could be valuable for short‐term implanted sensors, extracorporeal devices, or single‐cell trapping technologies, suggesting potential applications for these designs. Collectively, these findings challenge the prevailing view that nanoscale texturing is essential for bio‐repellency, demonstrating that well‐engineered microscale architectures, which are easier fabricate and replicate, can offer superior and sustained resistance to cell adhesion. This work establishes a design framework for anti‐fouling biomaterial interfaces, emphasizing the need to balance solid fraction to optimize air‐layer longevity while minimizing cell contact for robust repellency. Although demonstrated here with a lung epithelial cell line, the plastron‐mediated physical repellency of microscale Cassie–Baxter state design is likely applicable to other adherent cell types involved in biofouling, such as fibroblasts and endothelial cells. This is due to the physical mechanism's independence from the biochemical cell–surface interactions (see Section S7, Supporting Information for literature variability and Section S8, Supporting Information for the rationale behind selecting the A549 cell line). Future work could explore the performance of these microtextures under flow or in vivo, and test whether combining microtexturing with molecular antifouling coatings (such as PEG) might extend the non‐fouling duration.

## 4 Experimental Section

### Surface Fabrication

The fabrication of silicon micropillared surfaces was done using a 4″ silicon wafer and began with the application of hexamethyldisilazane (HMDS) as an adhesion promoter using a Vapor Prime Oven (YES‐3). Next, photolithography was carried out by spin‐coating the wafer with AZ 5214E photoresist (MicroChemicals) at 4000 rpm for 30 s. The coated wafer was then soft‐baked at 90 °C for 2 min, exposed for 3 s through various photomasks (Süss MicroTec MA‐6, 365 nm wavelength), and developed in AZ 351B (Merck) for 1 min. The unmasked regions were etched via BOSCH DRIE (Oxford PlasmaPro 100 Estrelas) with suitable cycle counts to obtain the required etching depth (40 µm). The etching parameters for this process included a processing temperature of 20 °C, pressures of 50 and 40 mTorr, ICP power levels of 1800 and 1500 W, and gas flows of 10 and 200 sccm for O_2_, and 300 and 10 sccm for SF_6_, alternating between the two steps (Etching and Passivation) of each cycle. After etching, the remaining photoresist was removed using ultrasonication in acetone and, if needed, oxygen plasma treatment. For superhydrophilic surfaces, no coating was applied. On the other hand, for superhydrophobic pillars, a hydrophobic fluoropolymer layer was deposited onto the wafer via a PECVD process (Oxford Plasmalab 80Plus) under conditions of 20 °C temperature, 250 mTorr pressure, 50 W power, and a CHF_3_ flow rate of 100 sccm. For the fabrication of the black silicon nanopillars, a maskless cryogenic DRIE technique was applied on a 4″ silicon wafer.^[^ ^14^, ^125^ ^]^ The process parameters (Oxford PlasmaPro 100 Estrelas) included a temperature of −125 °C, a pressure of 5 mTorr, ICP power set to 1500 W, forward power at 10 W, and gas flows of 15 sccm for O_2_ and 30 sccm for SF_6_. For the superhydrophobic nanopillars, the wafer was subsequently coated with the same hydrophobic fluoropolymer layer used for the micropillared surfaces. The hydrophobic smooth control was made by direct fluoropolymer coating on a polished silicon wafer, and the hydrophilic control consisted of standard tissue‐culture‐treated 24‐well plates (Corning, polystyrene, flat‐bottom). The surfaces were diced into 1.0 cm × 1.0 cm chips.

### Contact Angle Measurement

The dynamic advancing and receding contact angles of water were measured using the needle‐in sessile drop method (THETA, Biolin Scientific). For the advancing contact angle, the droplet size was increased from 2 to 5 µL, while the receding contact angle was obtained by gradually reducing the droplet size from 5 to 0 µL. Both were done at a flow rate of 0.1 µL s^−1^.

### Protein Adsorption

Chips of 1.0 cm × 1.0 cm were placed in 24 well‐plate and incubated at 37 °C and 5% CO_2_ with 2.0 mg mL^−1^ FITC–BSA for 4 h. The protein solution was then aspirated, and the chips were washed with phosphate‐buffered saline (PBS) three times and then dried. The chips were then imaged using fluorescence microscopy (Excitation wavelength: 443–489 nm, Emission wavelength: 497–551 nm).

### Cell Culture

A549 was a human lung adenocarcinoma epithelial cell line; cells were maintained in RPMI‐1640 medium supplemented with 1% penicillin–streptomycin (Gibco), 1% GlutaMAX (Gibco), and 10% fetal bovine serum (Gibco) at 37 °C, 5% CO_2_ in a humidified incubator.

### Cell Adhesion Study

An epithelial cell model was selected for its sensitivity to surface wettability (Section S8, Supporting Information). To investigate the effect of superhydrophobicity and surface's wettability and structure on cell adhesion, surfaces with different wettability and topography were prepared and incubated with A549 human lung epithelial cells. Chips of 1.0 cm × 1.0 cm, with different pillar diameters and pitches (Table S1 and Section S9, Supporting Information), along with other tested samples, including the nanopillars and controls, were placed into a 24‐well plate. All results were obtained from experiments conducted using the cell line during passages 4–7 at 90% confluency to ensure consistency and minimize the risk of significant variations or mutations. Before cell seeding, all surfaces were sterilized by immersion in 70% ethanol for 5 min and then exposed perpendicularly to UV light in a biosafety cabinet for 30 min. A549 cells were suspended in media at a final seeding density of 2 × 10^5^ cells mL^−1^, and 1 mL of this suspension (1052 cells mm^−2^) was added to each well containing one chip, as well as to chip‐free wells serving as the hydrophilic control. The well plate was gently shaken in a north–south and east–west motion to ensure uniform cell distribution. The plate was incubated at 37 °C and 5% CO_2_ in humidified incubator for 4, 24, or 72 h. Plastron reflectivity was assessed at the three timepoints. After incubation, the cell culture media was aspirated from the wells, and the chips were washed three times with 1 mL of PBS to remove any non‐adherent cells. The adhered cells were fixed by incubating each chip with 1 mL of 4% glutaraldehyde solution for 30 min at room temperature.

### Fluorescence Microscopy Imaging

Fluorescence imaging was performed to assess the adhesion and morphology of A549 cells on the surfaces at the three timepoints. Following cell fixation, the nuclei were stained with DAPI (4.5 µm, excitation: 352–402 nm, emission: 417–477 nm), while the actin cytoskeleton was labeled using Alexa Fluor 568‐phalloidin (1:40 dilution, excitation: 539–585 nm, emission: 601–683 nm). Staining was conducted at 37 °C for DAPI and at room temperature for Alexa Fluor 568‐phalloidin, each followed by three PBS washes. Fluorescence images were acquired using a Nikon ECLIPSE Ni‐∈ microscope at 10 × (low), 20 ×, or 50 × (high) magnification. Bright field images were recorded for structural reference, and merged fluorescence images provided a comprehensive view of both nuclear and cytoskeletal organization.

### SEM Imaging

To examine A549 cell adhesion and morphology closely on the surfaces, samples were prepared for SEM following fixation and critical drying steps. After incubation for 4, 24, or 72 h, the cells were washed and fixed as mentioned before. This was followed by a deionized water rinsing step to remove residual salts and prevent crystallization artifacts during drying. For dehydration, samples underwent a graded ethanol series (30%, 50%, 70%, 90%, and 100%), with each step lasting 10 min to ensure complete removal of water. To achieve optimal preservation of cell morphology, HMDS drying was performed by replacing the final 100% ethanol wash with HMDS for 10 min, followed by air drying in a fume hood. Once fully dried, the samples were mounted on aluminum SEM stubs using carbon tape and sputter‐coated with a 10 nm gold layer to enhance conductivity and minimize charging effects during imaging. SEM imaging was carried out at an appropriate accelerating voltage to visualize cell attachment, morphology, and interactions with the structured surfaces.

### Image Analysis

Fluorescence images of DAPI‐stained nuclei at 10 × magnification were analyzed using ImageJ (Fiji, NIH)^[^ ^126^ ^]^ to quantify A549 cell adhesion on the studied surfaces. Images were organized by surface type and incubation time and batch‐processed using a recorded ImageJ macro to ensure consistency. Each image was converted to 8‐bit grayscale, and the “Scale when converting” option was enabled to maintain accurate dimensions. The “Threshold” function was applied to segment nuclei, followed by background subtraction to reduce noise. To separate closely clustered nuclei, the “Watershed” function was applied before converting images to binary masks. Nuclei were then counted using the “Analyze Particles” function, applying a size minimum threshold to exclude any artifacts (Figure S55, Supporting Information). The macro script was executed in Batch Process Macro mode, automating image analysis across all samples.

### Statistical Analysis

Statistical analyses were performed using OriginPro 2024b. Nuclei counts were normalized to cell density (cells mm^−2^). For the 4 h timepoint, data were collected from *n* = 6 samples, each with 3 randomly selected 1 mm × 1 mm fields. At 24 and 72 h, *n* = 3 samples. Data are reported as mean ± SD. Box plots show the interquartile range (25–75th percentile), with horizontal lines indicating the mean and whiskers representing SD. Violin plots display the kernel‐smoothed distribution of cell density (cells mm^−2^). Overlaid boxes represent the standard error, with the horizontal line indicating the mean and whiskers denoting SD. One‐way ANOVA followed by Tukey's post hoc test was used to compare groups with significance defined at *p* < 0.05.

## Conflict of Interest

The authors declare no conflict of interest.

## Author Contributions

M.A. designed and fabricated all surface structures, performed wettability and plastron stability measurements, and conducted protein adsorption study, cell culture, cell adhesion experiments, fluorescence and SEM imaging, and data analysis. M.A. and V.J. jointly conceived the research plan. M.A. wrote the manuscript, and V.J. acquired funding, provided supervision, critical feedback, and manuscript revisions.

## Supporting information



Supporting information

## Data Availability

The data that support the findings of this study are available from the corresponding author upon reasonable request.
