# Supplementary material for: Superhydrophobic Cell‐Repellent Microstructures: Plastron‐Mediated Inhibition of A549 Epithelial Cell Adhesion
Source: Small. 2025 Aug 19;21(40):e06022. doi: 10.1002/smll.202506022 (PMC12508712; doi:10.1002/smll.202506022)
Supplement: Supplementary file 1 — Supporting information [file SMLL-21-e06022-s001.pdf]

NANO · MICRO
small

Supporting Information

for *Small*, DOI 10.1002/smll.202506022

Superhydrophobic Cell-Repellent Microstructures: Plastron-Mediated Inhibition of A549 Epithelial Cell Adhesion

Mohammad Awashra and Ville Jokinen**

Supporting Information

Superhydrophobic Cell-Repellent Microstructures: Plastron-Mediated Inhibition of A549 Epithelial Cell Adhesion

Mohammad Awashra and *Ville Jokinen*

School of Chemical Engineering, Department of Chemistry and Materials Science, Aalto University, Tietotie 3 Espoo 02150, Finland

mohammad.awashra@aalto.fi, *ville.p.jokinen@aalto.fi*

Keywords: superhydrophobic, microstructures, biointerface, physical repellency, cell adhesion, air plastron

Contents

S0. Why Microstructured Surfaces Remain Understudied 3
S1. Geometry-Dependent Wetting Regimes 5
S2. Plastron Stability and Protein Repellency 9
S3 Effect of Wettability and Roughness on Cell Adhesion 12
S4 Micropillar Solid Fraction Effect on Cell Adhesion 31
S5. Long-Term Influence of Pillar Size on Cell Adhesion 34
S6. Effect of Pillar Height on Plastron Stability 64
S7. Why Reported Cell Adhesion Results on Superhydrophobic Surfaces Vary 65
S8. Epithelial vs. Fibroblasts as a Cell Model – Rationale for Selecting A549 Cells 69
S9. Experimental Section 71
Supporting References 73

S0. Why Microstructured Surfaces Remain Understudied

Most of the existing literature focuses on cell Repellency on nanostructured surfaces rather than microstructured ones, leaving the effects of microscale superhydrophobic surfaces largely unexplored. Nanostructures impact cell adhesion by altering protein adsorption and integrin clustering, which govern focal adhesion formation and attachment strength. For instance, Wu et al.[1] studied A549 epithelial cells spreading on nanopatterned surfaces and found that lamellipodia dominated on Si nanoholes arrays, while filopodia were more prominent on Si nanopillars arrays, indicating varied adhesion forces depending on the nanostructure type. Moreover, a study by Flamourakis et al.[2] demonstrated that neuronal cells responded to nanopillar arrays with tunable mechanical properties by aligning their neurites according to the nanostructure topology, highlighting the role of topographical and mechanical cues in guiding cellular interactions. Microstructures, on the other hand, primarily influence cell alignment, orientation, and spreading by providing physical cues.[3] For example, Bourkoula et al.[4] showed that cells achieved optimal alignment on micropatterned silicon stripes, with stripe widths of 20–22.5 μm yielding over 95% alignment fidelity. Vu et al.[5] demonstrated that PDMS micropillars, despite lacking superhydrophobic properties or a plastron layer, significantly influence cancer cell dynamics, with pillar diameter and surface energy playing critical roles in guiding cell adhesion, spreading, and clustering. While microscale structures have been extensively studied for their role in cell guidance, alignment, and adhesion, investigations into microscale superhydrophobic surfaces remain limited. This is partly due to the challenges in achieving superhydrophobicity at the microscale alone, without incorporating hierarchical or nanoscale features. Consequently, there is a notable gap in the literature regarding the influence of microscale-only superhydrophobic surfaces on cell Repellency, as most studies focus on nano or hierarchical structures that combine both scales. A study by Hejazi et al.[6] observed that

nanostructured superhydrophobic surfaces significantly reduced adhesion and proliferation of 4T1 mouse mammary tumor cells compared to 2 µm spheres. Moreover, Ranella et al.[7] demonstrated the influence of surface nano/micro roughness and wettability on cell behavior. Using hierarchical micro- and nanostructured silicon pillars (≈1 µm tip size), they found that low-roughness hydrophobic surfaces facilitated cell adhesion and spreading, while high-roughness superhydrophobic surfaces inhibited adhesion and promoted cell clustering. Wang et al.[8] reported that cell adhesion was guided by heterogeneously wetted patterned 30, 50, and 100 µm SU-8 pillars, where cells selectively adhered to hydrophilic pillar tops only due to the air plastron that is retained through the "Petal effect". Their findings reinforce the notion that wetting heterogeneity, rather than just topographical scale, plays a critical role in directing cell attachment.

Our study directly addresses this research gap in microscale-only superhydrophobic cell-repellent surfaces by employing precisely defined micropillar arrays with systematically varied pillar pitch, diameter, and solid fraction, while maintaining a fixed pillar height (40 µm) shown previously to enhance plastron stability. We explore a controlled design space, quantifying the critical conditions for plastron collapse and their correlation with cell adhesion behavior. By examining wetting transitions, plastron lifetime, and biofouling over time under realistic biofluid conditions, our work demonstrates for the first time that microscale-only structures can achieve superior cell Repellency *via* physical air entrapment alone. This overcomes previous limitations by providing a scalable, chemically passive strategy for anti-adhesion surfaces.

S1. Geometry-Dependent Wetting Regimes

Contact angle measurements revealed a transition from hydrophilic behavior on uncoated smooth silicon to superhydrophobic Cassie–Baxter states on textured, coated surfaces. Water droplets on the micropillar surfaces (5 μm diameter pillars) and nanopillars exhibited extremely high advancing (θ_{Adv}) and receding (θ_{Rec}) contact angles, confirming the presence of a stable air plastron that resists wetting. Notably, the nanopillar surface achieved the highest contact angles among all samples, surpassing the microstructured surfaces. In contrast, superhydrophilic non-coated structured silicon displayed complete wetting (~0°), while the smooth non-coated silicon had a hydrophilic nature (~33°), and the hydrophobic coated smooth control had only moderate contact angles $\theta_{Adv}/\theta_{Rec}$ (~113°/85°).

Systematic variation of pillar fraction and size confirmed their influence on wettability. Reducing the solid fraction of 5 μm pillars from 22.7% to 2.5% produced a marked increase in receding contact angle (from ~141° to ~170°), consistent with the Cassie–Baxter model as lower solid–liquid contact area has decreased droplet adhesion forces. Conversely, increasing the pillar diameter (at fixed solid fraction) tended to reduce the apparent contact angles, indicating easier liquid impalement for larger features.[9] For example, at 7.4% solid fraction, surfaces with larger pillars showed lower θ_{Rec} than those with smaller pillars (**Figure 1m** and **Figure S3**), implying that finer texture aids plastron formation. Overall, our surfaces span distinct wetting regimes from superhydrophilic (uncoated rough silicon) to superhydrophobic (coated micro/nanopillars), providing an ideal platform to examine how surface morphology and wetting state govern bio-interfacial interactions.

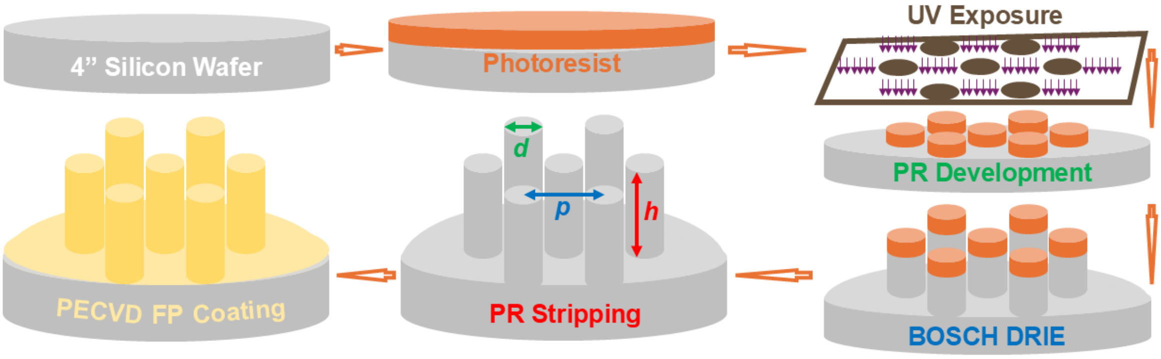

Figure S1. Schematic of the fabrication process of superhydrophobic micropillars.

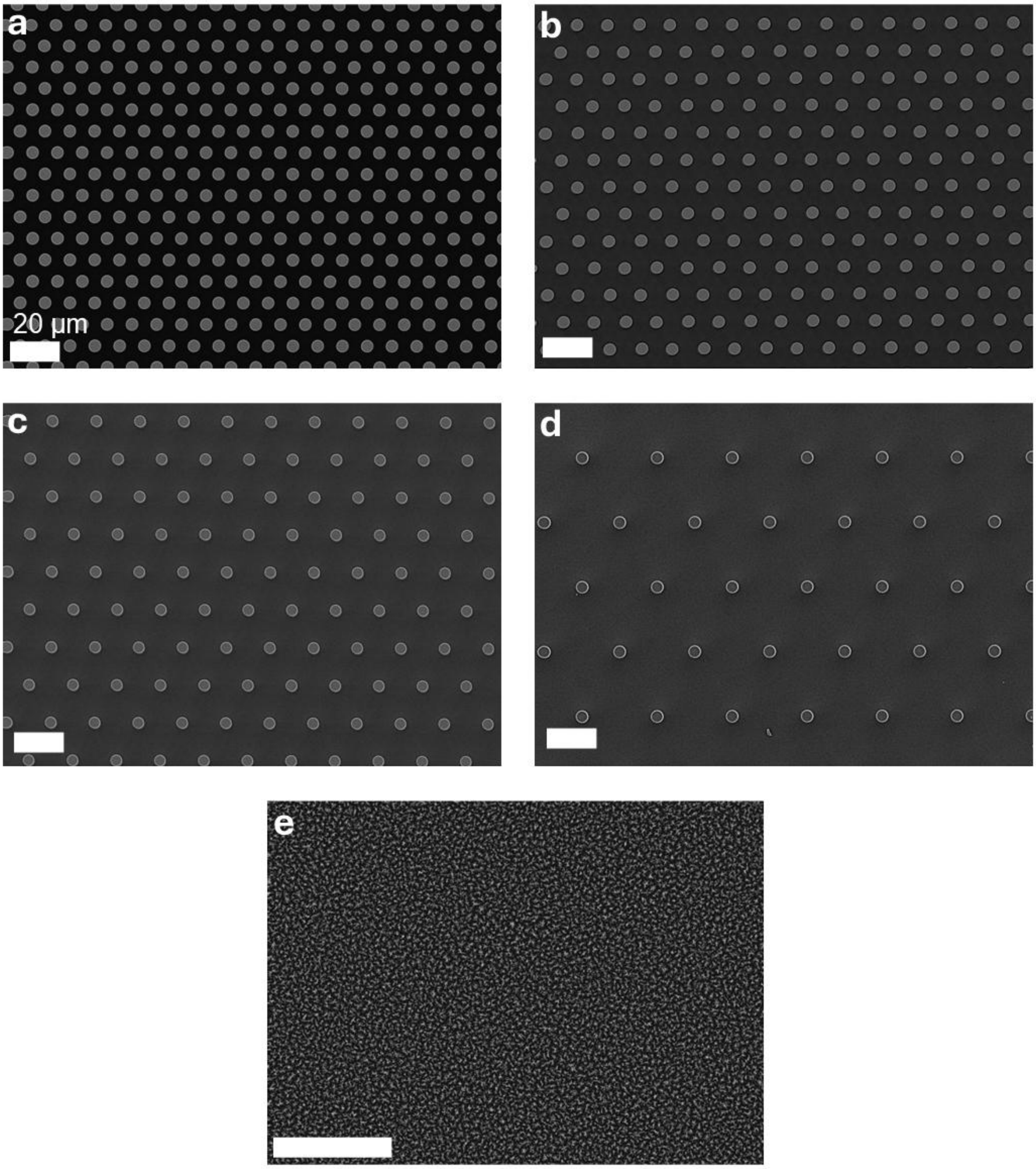

Figure S2. (**a–d**) SEM images showing top view of 5 µm silicon pillars with varying solid fractions (22.7%, 14.5%, 7.4%, and 2.5%, respectively). (**e**) Si nanopillars.

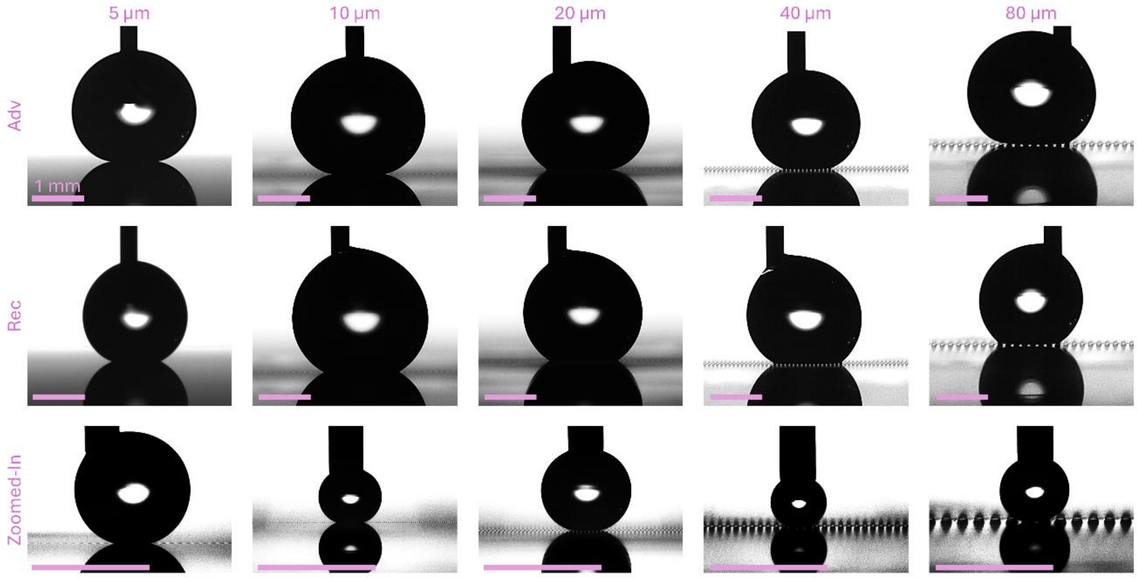

Figure S3. Images of advancing (Adv), receding (Rec), and zoomed-in view of water contact angles of on different pillars size (solid fraction: 7.4%). Scale bars: 1 mm.

S2. Plastron Stability and Protein Repellency

In fact, protein coverage on the 2.5% surface was comparable to the much higher 22.7% fraction surface, indicating that an unstable plastron can undermine anti-fouling performance. These observations align with prior reports that superhydrophobic nanostructures drastically curtail protein adsorption by minimizing contact area and intermolecular interactions. Zhang et al.[10] further confirm that protein adsorption decreases on nanostructured superhydrophobic surfaces due to minimized solid–liquid contact and weakened van der Waals and hydrogen-bond interactions. Koc et al.[11] found that microscale roughness can lead to increased protein fouling compared to nanoscale roughness when the air plastron is compromised. This aligns with the observed higher protein adsorption on the lowest solid fraction (2.5%), where certain regions transitioned to the Wenzel state. We found that plastron longevity increased with solid fraction: by tracking the solid–liquid interface area over time (**Figure 1p**), we observed that surfaces with 22.7% and 14.5% solid retained their plastron for up to 72 h, whereas the 7.4% plastron collapsed after ~24 h, and the 2.5% plastron after less than 4 h . Thus, higher pillar density (and smaller air gaps) confers greater plastron stability over days, at the cost of higher initial solid–liquid contact. In contrast, sparse pillars create larger initial air gaps that superbly resist protein adsorption short-term, but their plastron is more vulnerable to decay, which aligns with our previous findings.[9] Together, these results demonstrate that maintaining a stable air film is critical for sustained protein-repellency: when intact, the plastron effectively blocks protein adsorption, but once it collapses, the exposed textured surface may even foul more than a smoother one. Protein adsorption on 5 µm pillars, quantified by fluorescence intensity (**Figure 1o**), follows the expected trend, with significantly lower protein coverage observed on surfaces with reduced solid fractions. For nanopillars, increasing curvature (decreasing size) has been shown to reduce the formation of protein aggregates on the surface.[11,12] In some cases

protein adsorption may be enhanced on superhydrophobic surfaces. For example, Zhao et al.[13] observed that protein adsorption was slightly enhanced on superhydrophobic nanowires relative to smooth Ti. However, proteins bind in an unfavourable manner due to the high curvature of nanostructured surfaces, yielding few integrin engagement points or a disrupted adhesion organization. **Figure S5** shows the shorter plastron stability of superhydrophobic micropillared surfaces after incubation with cell for 4 h indicating that protein adsorption changes the surface chemistry of the surface and renders it less hydrophobic.

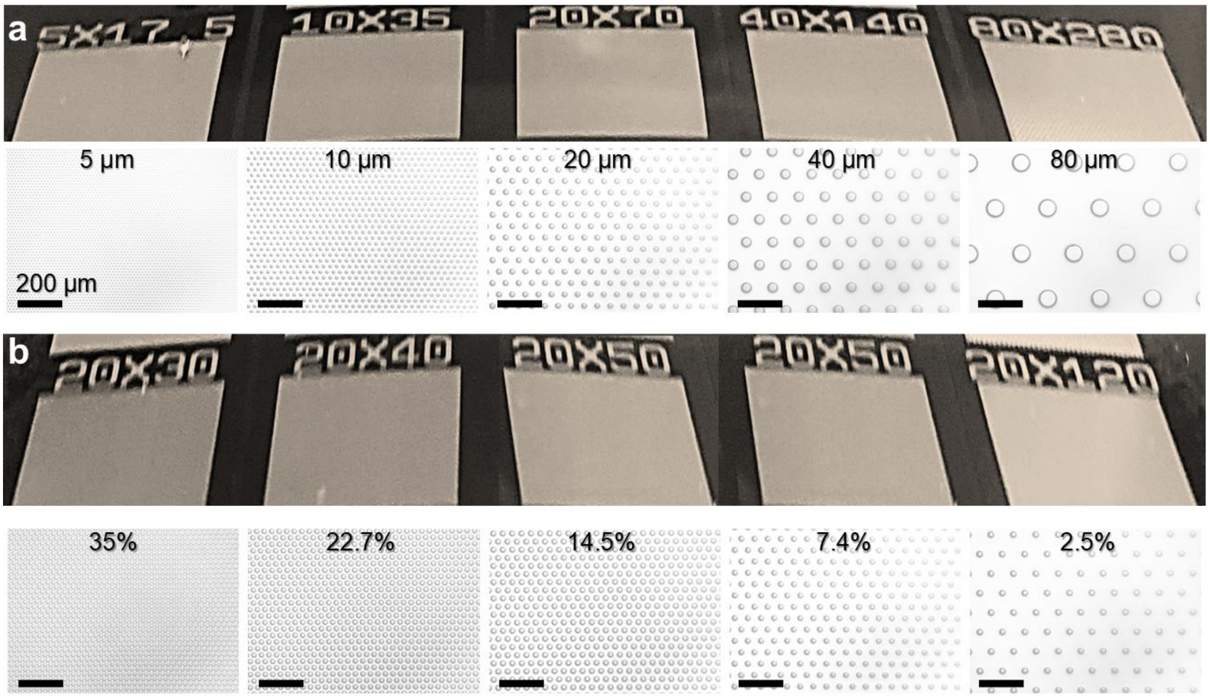

Figure S4. Photograph of plastron layer showing mirror-like reflection underwater (top) and a bright field optical image of the corresponding pillars (bottom) with different (**a**) diameters and (**b**) solid fractions.

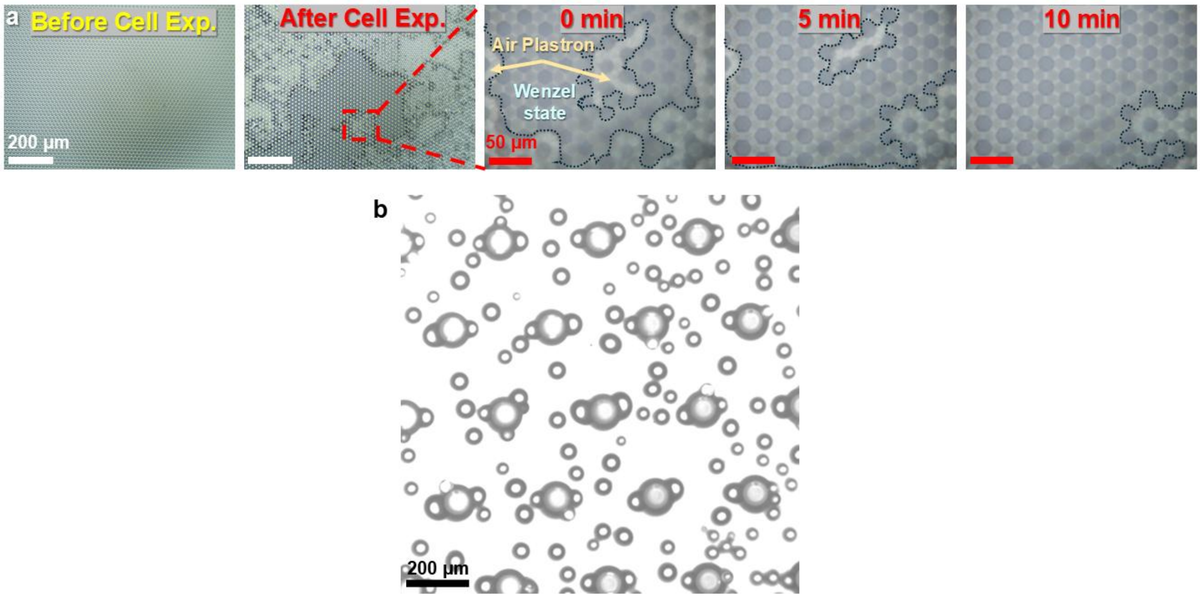

Figure S5. (**a**) Bright-field images showing the superhydrophobic micropillared surface before and after cell experiment. The plastron layer is disrupted after cell experiment, transitioning from a Cassie–Baxter to a Wenzel state, as seen in the time-lapse images (0 min, 5 min, and 10 min). The water–air interface retreat is outlined (dotted line). Scale bars: 200 µm (left), 50 µm (right). (**b**) Optical microscopy image displaying the formation of small and separate air bubbles in the Wenzel state. Scale bar: 200 µm.

S3 Effect of Wettability and Roughness on Cell Adhesion

Compared to smooth hydrophilic silicon control (contact angle ~33°) (**Figure S6**), superhydrophilic nano and micropillars (I, II) exhibited significantly higher cell densities. Meng et al.[14] reported similar findings when comparing superhydrophilic nanostructured surfaces to hydrophilic controls. This is because of 1) the lack of topography on smooth surfaces which weakens the cell adhesion 2) the increased cell contact area on nano and microstructured surfaces (**Figure 2e**). This also explain the cell repellent properties of the smooth hydrophobic silicon (IV, contact angle ~113°).

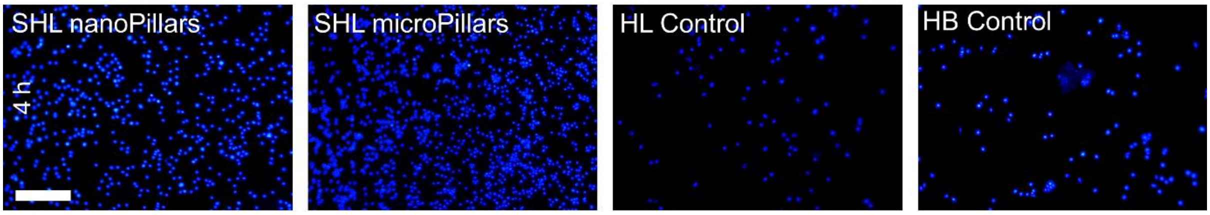

Figure S6. Low magnification fluorescence microscopy images showing adhered cells on superhydrophilic (SHL) nano and micropillars, as well as hydrophilic (HL) and hydrophobic (HB) control after 4 h of incubation. Scale bar: 200 μm.

superhydrophilic nanopillars promote strong cell adhesion and rapid proliferation, leading to full confluency after 3 days (**Figure S7**). The isotropic cell spreading in all directions is attributed to the high and uniform surface roughness of the nanopillars. As observed in the SEM images (**Figure S8**), well-developed filopodia interact with hundreds of nanotips, while widespread lamellipodia extend progressively over time, suggesting active cytoskeletal remodelling and strong substrate interaction. Marcon et al.[15] observed similar cell spreading on superhydrophilic diamond nanowires, while the superhydrophobic ones effectively inhibited cell adhesion. Kontziampasis et al.[16] observed that oxygen plasma nanotexturing of PMMA surfaces significantly influenced 3T3 fibroblast adhesion and proliferation. While nanotextured surfaces initially enhanced cell attachment compared to flat PMMA, prolonged culture (3 days) resulted in reduced cell proliferation, particularly on highly nanostructured surfaces, suggesting that excessive nanoscale roughness may hinder long-term cell viability. Piret et al.[17] concluded that the penetration of cell cytoplasmic projections into the superhydrophilic silicon nanowire layer promotes strong adhesion by enabling intimate surface contact, aligning with our observation.

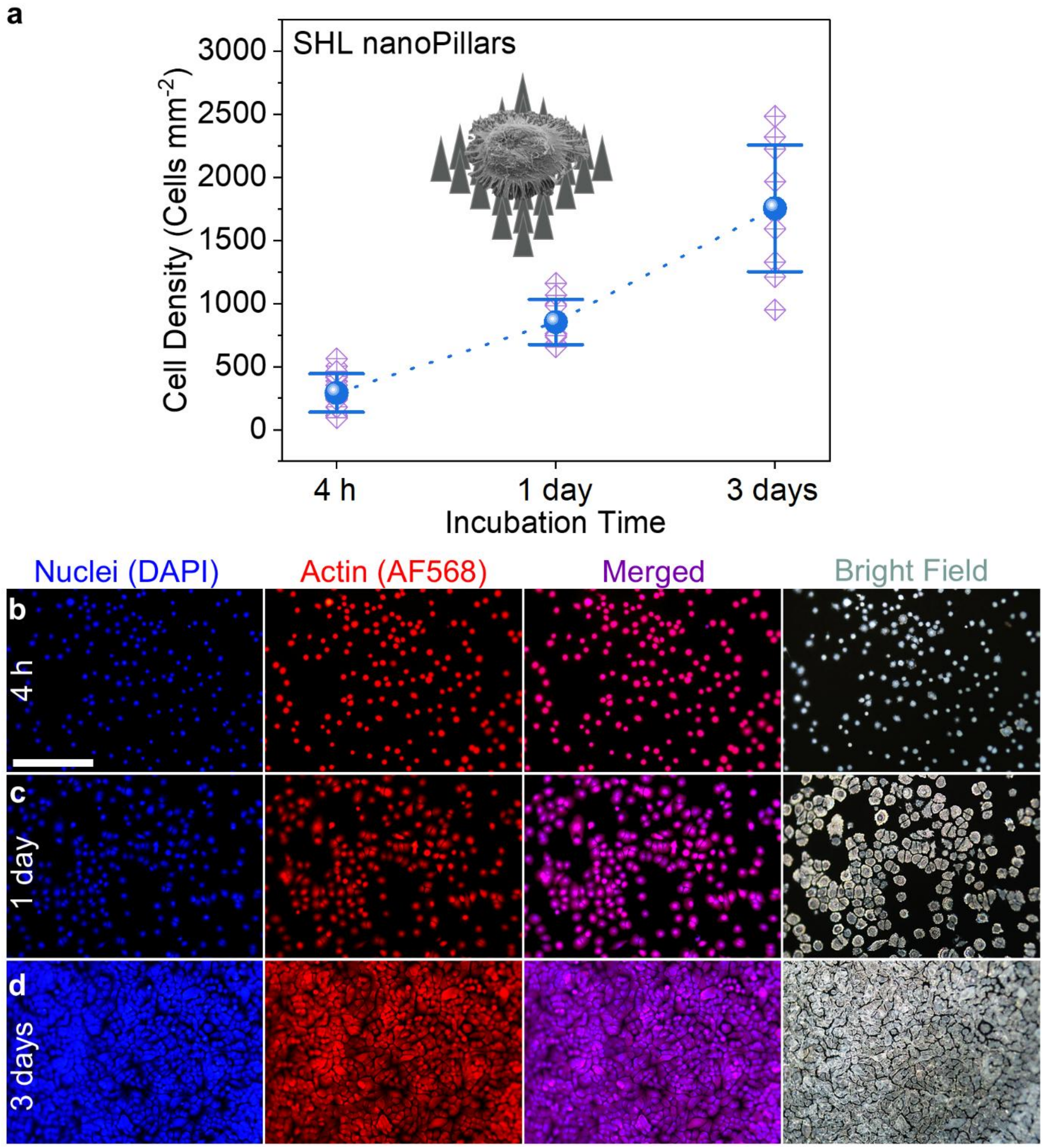

Figure S7. **Superhydrophilic nanopillars**. Temporal evolution of cell adhesion on superhydrophilic nanopillar surface. **a**) Cell density over time (4 h, 1 day, 3 days) on superhydrophilic nanopillars, showing a progressive increase in adhesion and proliferation. The inset illustrates a schematic representation of cell interaction with nanopillars. **b–d**) Fluorescence and bright-field microscopy images of adhered cells at different time points, showing nuclei (DAPI, blue), actin cytoskeleton (AF568, red), and merged images. The bright-field images provide structural context. Scale bar: 200 µm.

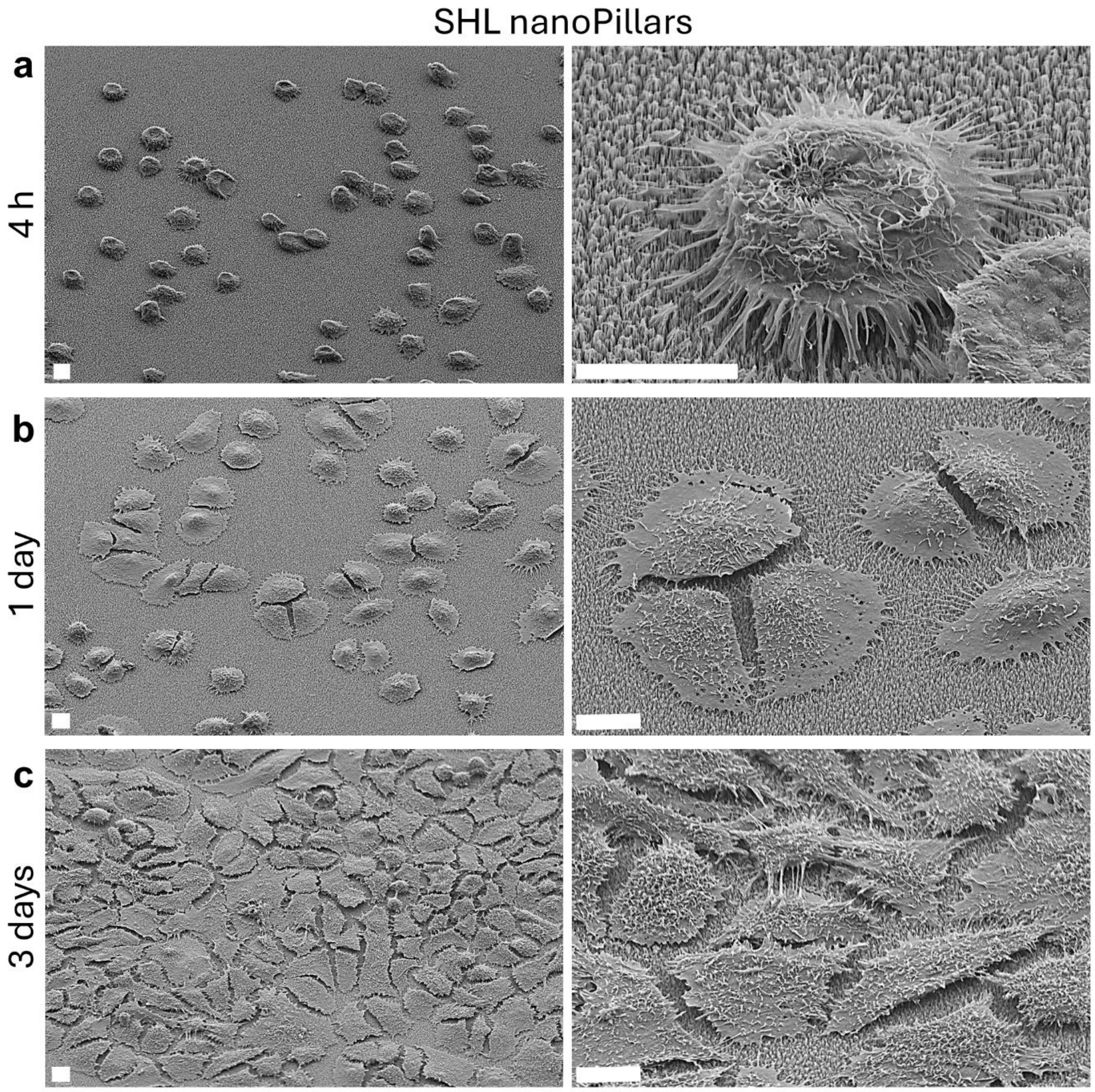

Figure S8. **Superhydrophilic nanopillars**. Scanning electron microscopy (SEM) images of A549 cells cultured on a superhydrophilic nanopillars. Scale bars: 10 µm.

Cell adhesion on superhydrophilic micropillars is significantly influenced by surface roughness, as evidenced by fluorescence microscopy (**Figure S9**) and SEM images (**Figure S10**). **Figure S9a** demonstrates a clear trend of increasing cell density with higher Wenzel roughness, attributed to an increased surface area. This trend persists across different incubation times (**Figure S11–14**), indicating that rougher micropillar surfaces promote stable adhesion and proliferation over time. This aligns with other reported studies.[7,18] Cell spreading is enhanced at lower roughness values due to greater spacing between the pillars and flatter areas (**Figure S12d**). After 3 days of incubation, all surfaces reached full confluency, with cell density showing a slight increase as roughness increased.

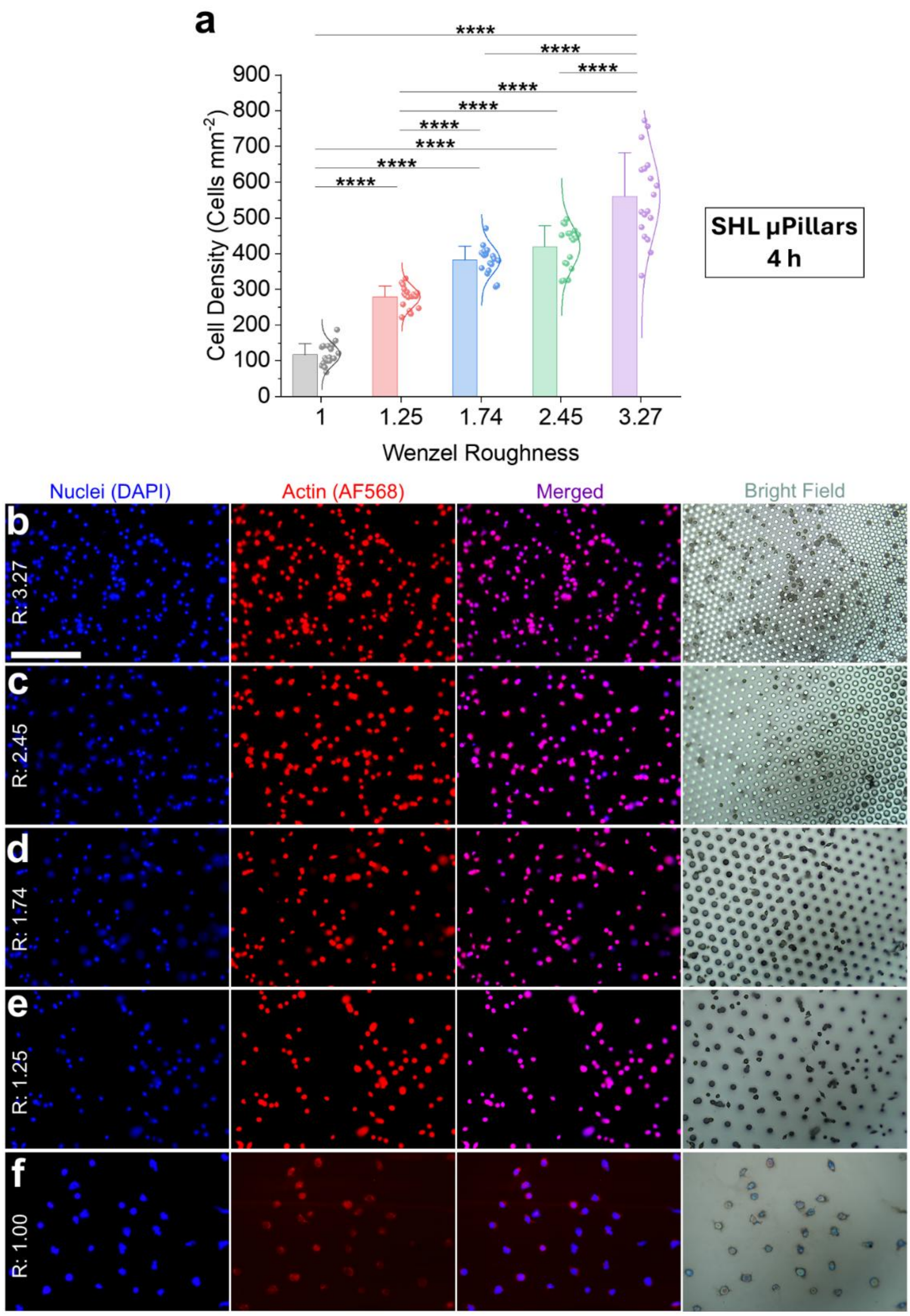

Figure S9. Superhydrophilic micropillars – 4 h Cell adhesion on superhydrophilic 10 µm pillar surfaces with varying Wenzel roughness after 4 h of incubation. **a**) Cell density as a function of Wenzel roughness, showing a significant increase in adhesion with increasing roughness. Statistical significance: $^*p < 0.05$, $^{**}p < 0.01$, $^{***}p < 0.001$, $^{****}p < 0.0001$. **b–f**) Fluorescence microscopy images of adhered cells on surfaces with different roughness values, showing nuclei (DAPI, blue), actin cytoskeleton (AF568, red), and merged channels. Bright-field images illustrate micropillar morphology. Scale bar: 200 µm.

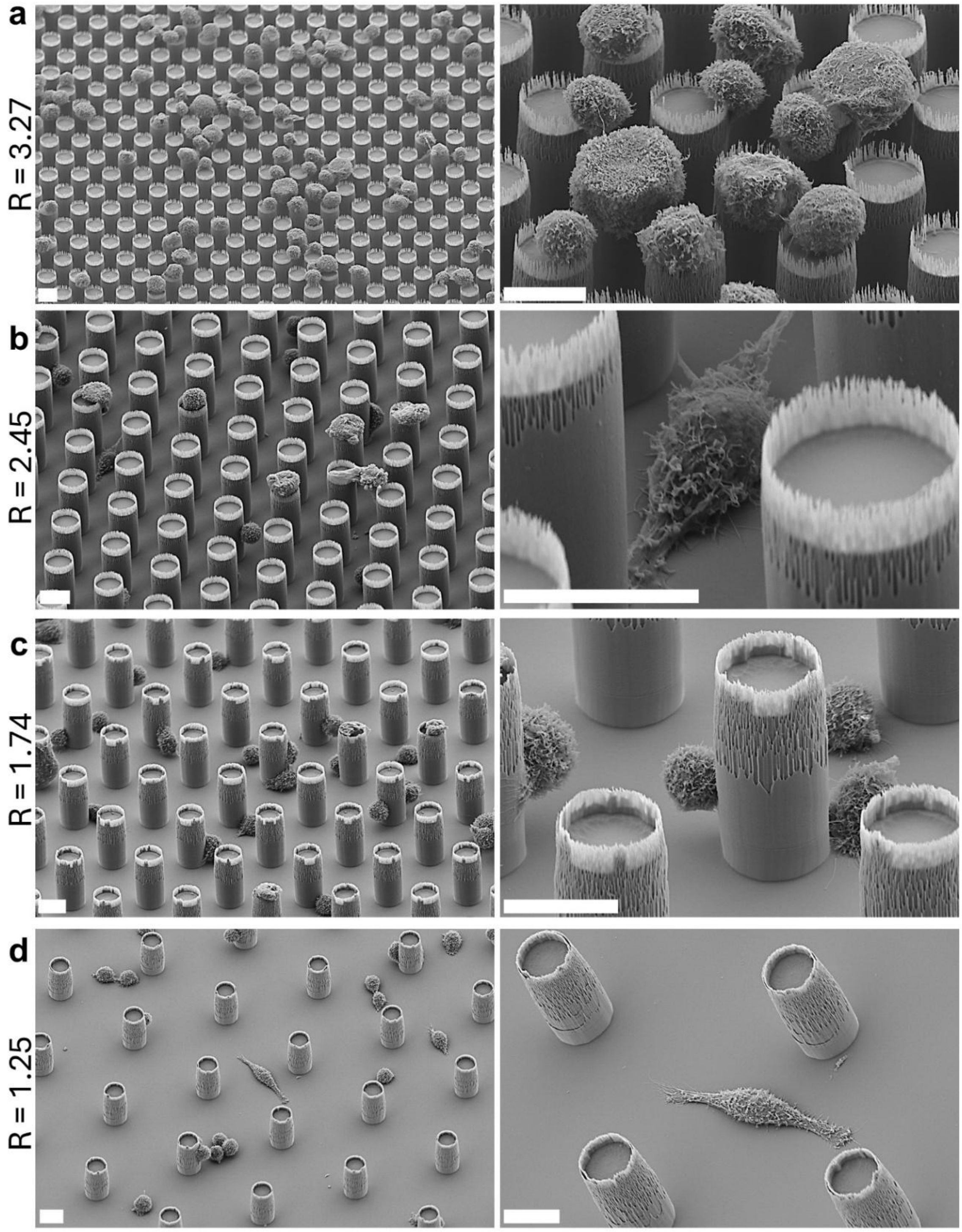

Figure S10. **Superhydrophilic micropillars – 4 h**. SEM images of superhydrophilic 10 μm pillar surfaces after 4 h of cell incubation, showing the effect of Wenzel roughness (R) on cell adhesion. **a–d**) Low (left) and high (right) magnification SEM images of micropillars with R = 3.27, 2.45, 1.74, and 1.25, respectively. Scale bars: 10 μm.

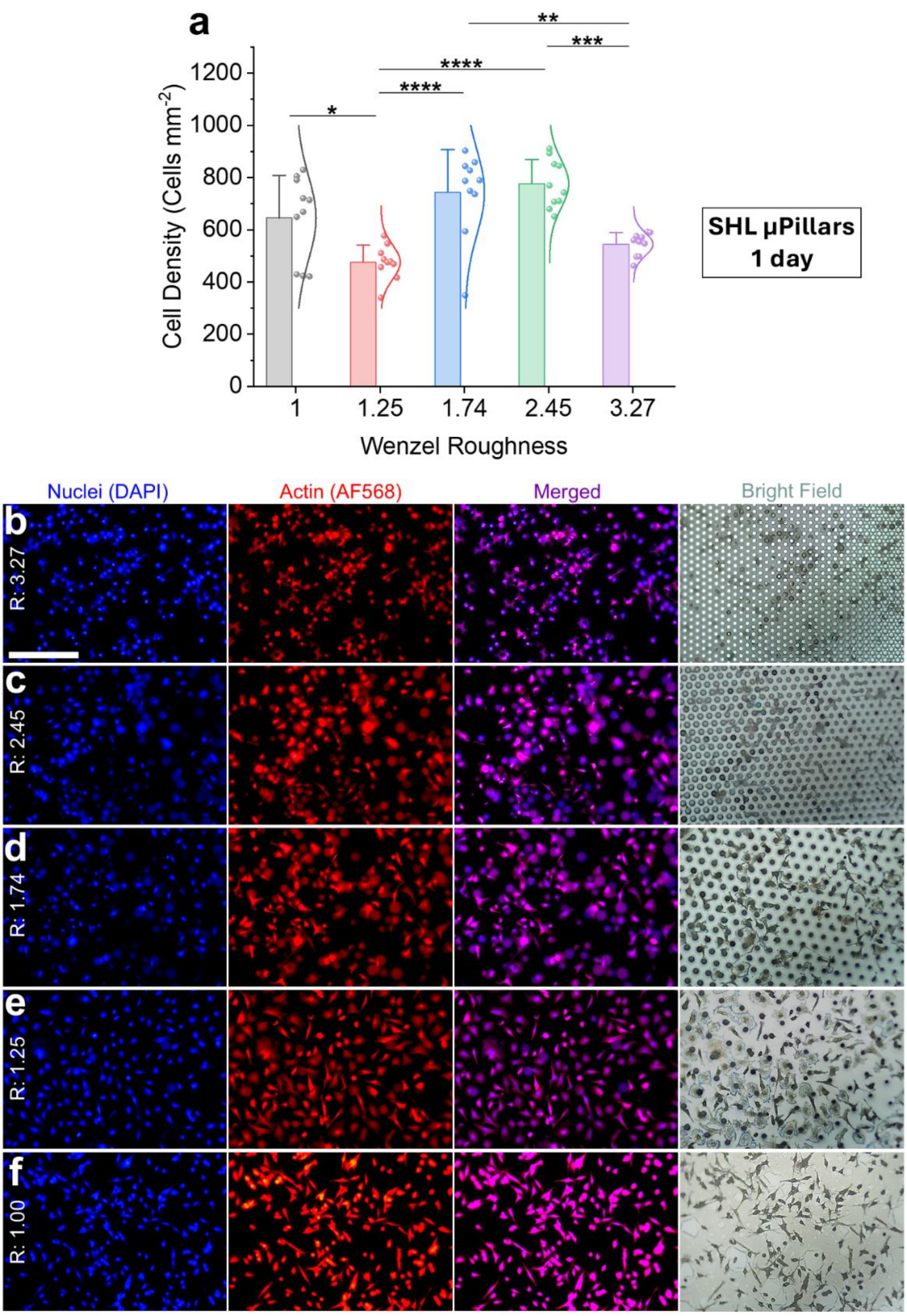

Figure S11. Superhydrophilic micropillars – 1 day. Cell adhesion on superhydrophilic 10 μm pillar surfaces with varying Wenzel roughness after 1 day of incubation. **a**) Cell density as a function of Wenzel roughness. Statistical significance: *$p < 0.05$, **$p < 0.01$, ***$p < 0.001$, ****$p < 0.0001$. **b–f**) Fluorescence microscopy images of adhered cells on surfaces with different roughness values, showing nuclei (DAPI, blue), actin cytoskeleton (AF568, red), and merged channels. Bright-field images illustrate micropillar morphology. Scale bar: 200 μm.

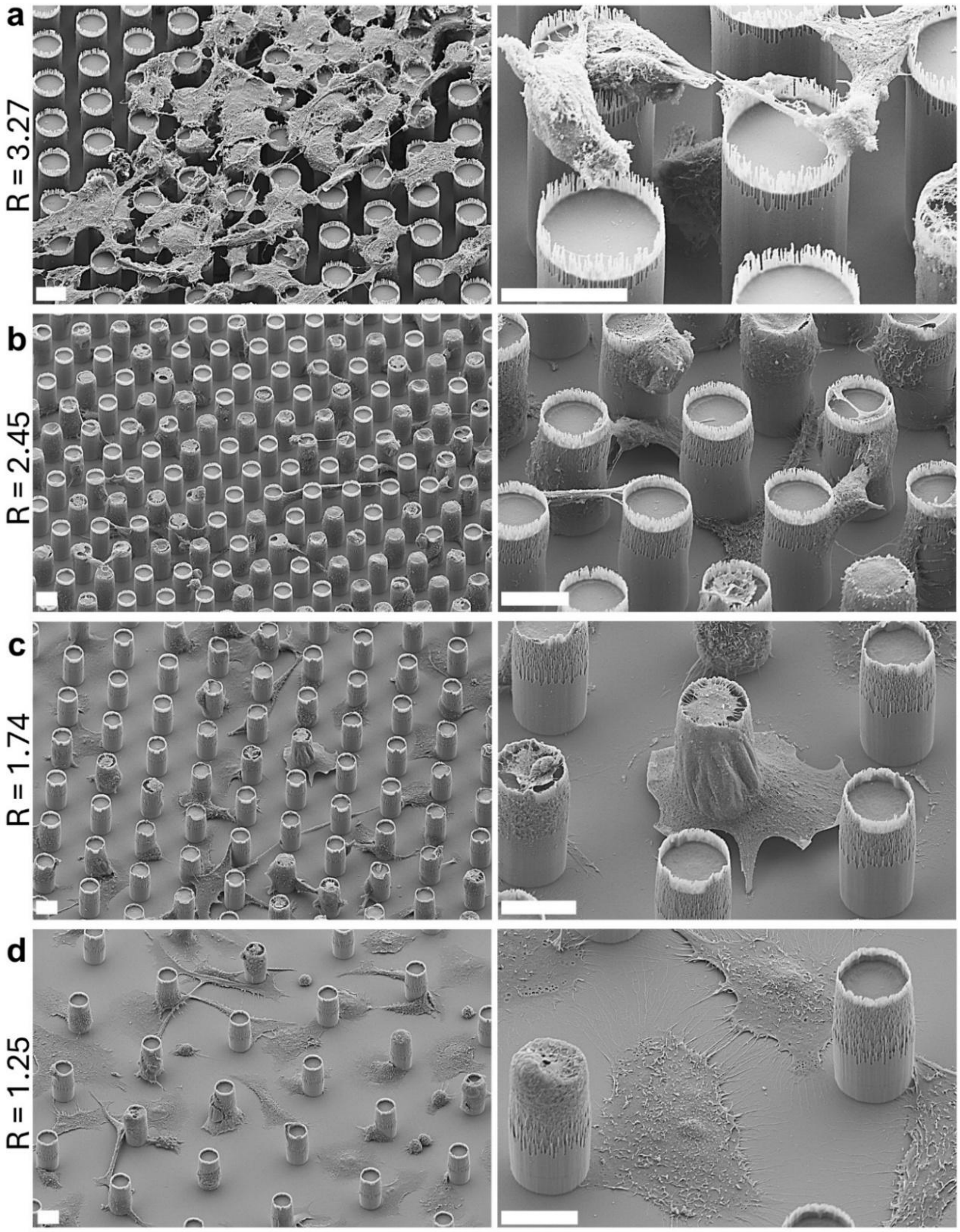

Figure S12. **Superhydrophilic micropillars – 1 day.** SEM images of superhydrophilic 10 µm pillar surfaces after 1 day of cell incubation, showing the effect of Wenzel roughness (R) on cell adhesion. **a–d**) Low (left) and high (right) magnification SEM images of micropillars with R = 3.27, 2.45, 1.74, and 1.25, respectively. Scale bars: 10 µm.

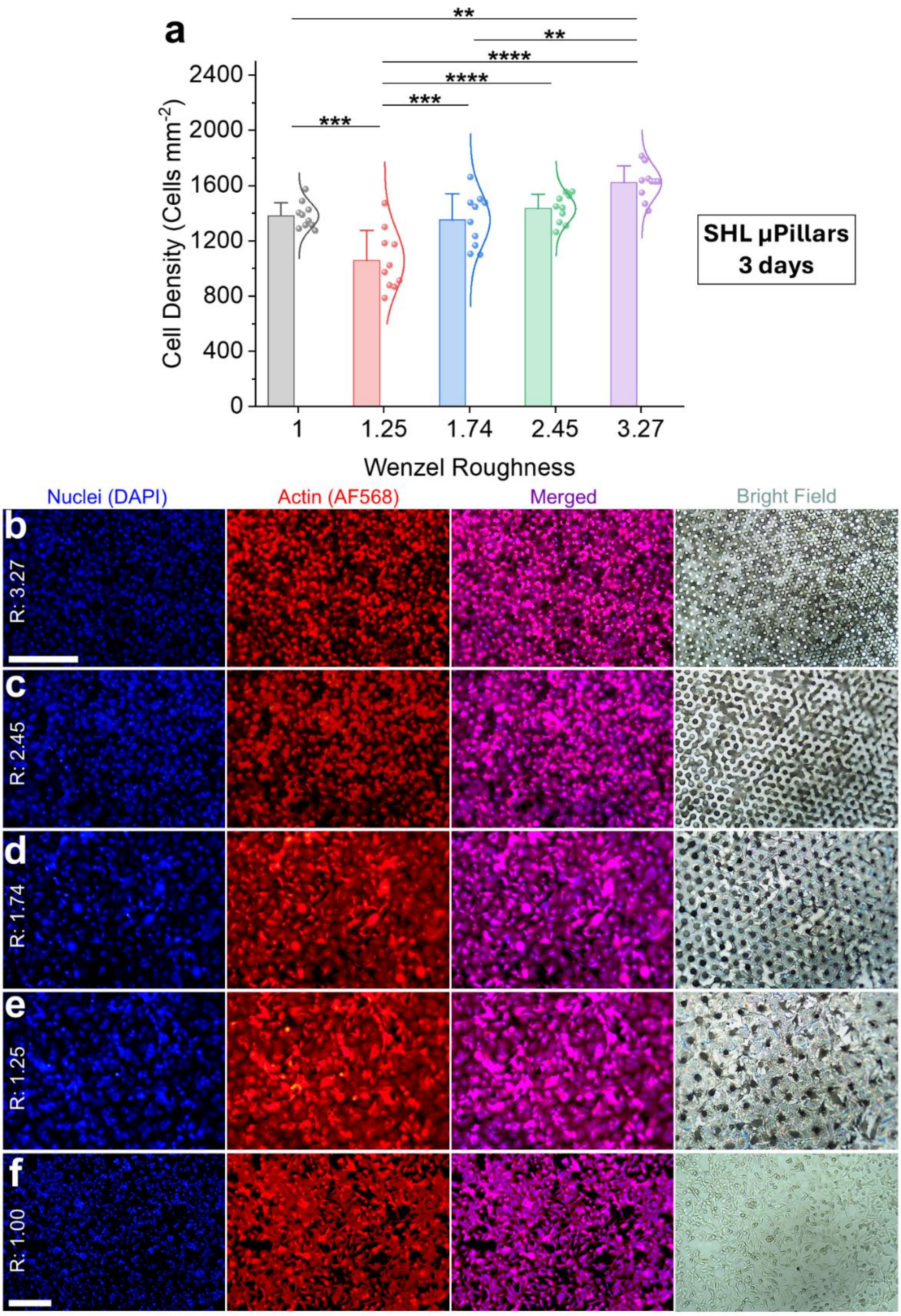

Figure S13. Superhydrophilic micropillars – 3 day. Cell adhesion on superhydrophilic 10 μm pillar surfaces with varying Wenzel roughness after 3 days of incubation. **a**) Cell density as a function of Wenzel roughness. Statistical significance: *$p < 0.05$, **$p < 0.01$, ***$p < 0.001$, ****$p < 0.0001$. **b–f**) Fluorescence microscopy images of adhered cells on surfaces with different roughness values, showing nuclei (DAPI, blue), actin cytoskeleton (AF568, red), and merged channels. Bright-field images illustrate micropillar morphology. Scale bar: 200 μm.

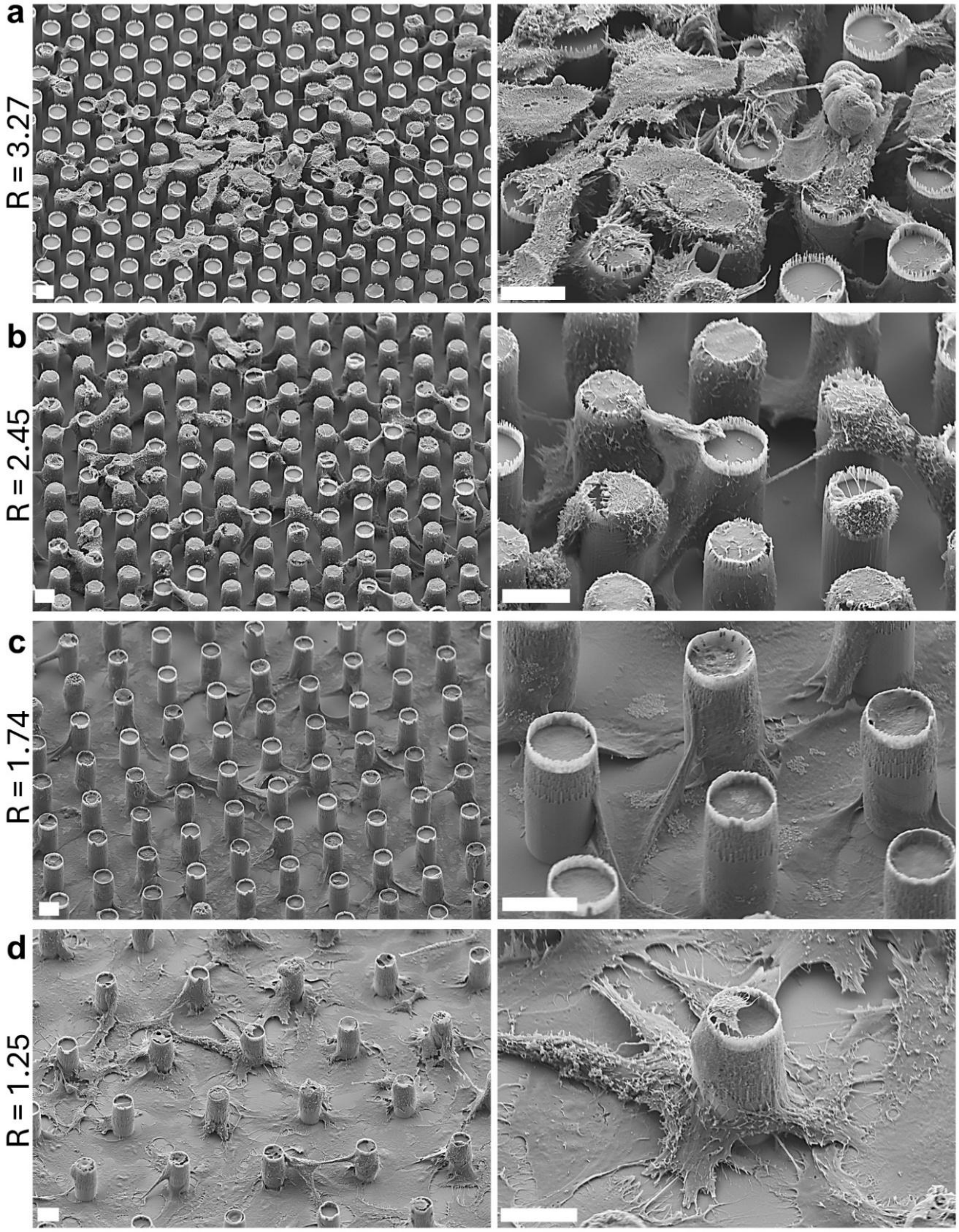

Figure S14. **Superhydrophilic micropillars – 3 day.** SEM images of superhydrophilic 10 µm pillar surfaces after 3 days of cell incubation, showing the effect of Wenzel roughness (R) on cell adhesion. **a–d**) Low (left) and high (right) magnification SEM images of micropillars with R = 3.27, 2.45, 1.74, and 1.25, respectively. Scale bars: 10 µm.

A549 cells exhibited a steady increase in adhesion and proliferation on the hydrophilic well plate, reaching full confluency by 3 days (**Figure S15**). The initial attachment at 4 h was followed by significant growth at 1 day, with a dense monolayer forming at 3 days. This proliferation trend aligns with the expected doubling time of A549 cells (22–30 h), indicating that the hydrophilic surface effectively supports adhesion, spreading, and division without inhibitory effects.

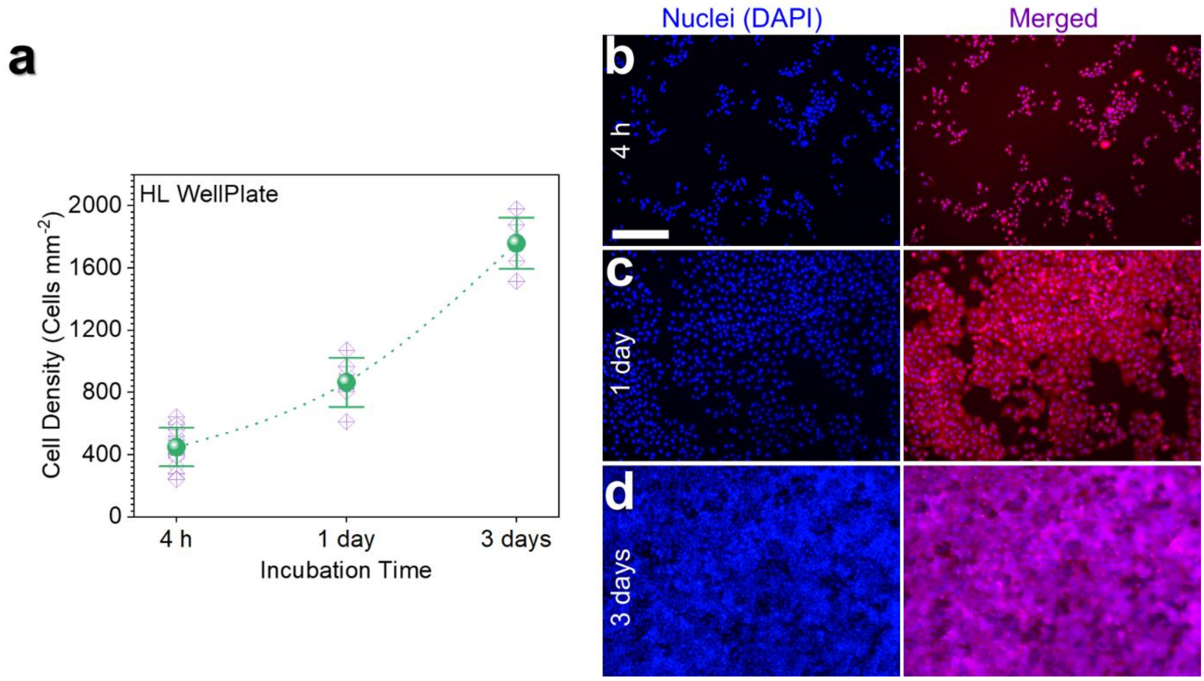

Figure S15. HL control. Proliferation of A549 cells on standard hydrophilic (HL) well plate over time. (**a**) Quantification of cell density at 4 h, 1 day, and 3 days of incubation, showing a steady increase in proliferation. (**b–d**) Fluorescence microscopy images of adhered cells stained for nuclei (DAPI, blue) and actin cytoskeleton (AF568, red) at different time points. Scale bar: 200 μm.

Cell adhesion and proliferation on the HB control surface increased over time, as demonstrated by both overall cell density (**Figure S16**) and high-magnification fluorescence images (**Figure S17**). At 4 h, only a few cells adhered, displaying a rounded morphology as shown in the SEM images (**Figure S18**). Ballester-Beltrán et al.[19] observed similar cell morphology on a porous superhydrophobic polystyrene that showed minimal binding, where cells were spherical with very few filopodia. By 1 day, cells exhibited enhanced spreading with prominent filopodia and lamellipodia extensions, facilitating stronger adhesion. At 3 days, cells reached near-confluency, forming an interconnected network with extensive actin cytoskeleton organization. This trend aligns with the expected behavior of A549 cells on hydrophobic substrates, where initial attachment is slower, but stable focal adhesions promote proliferation over time.

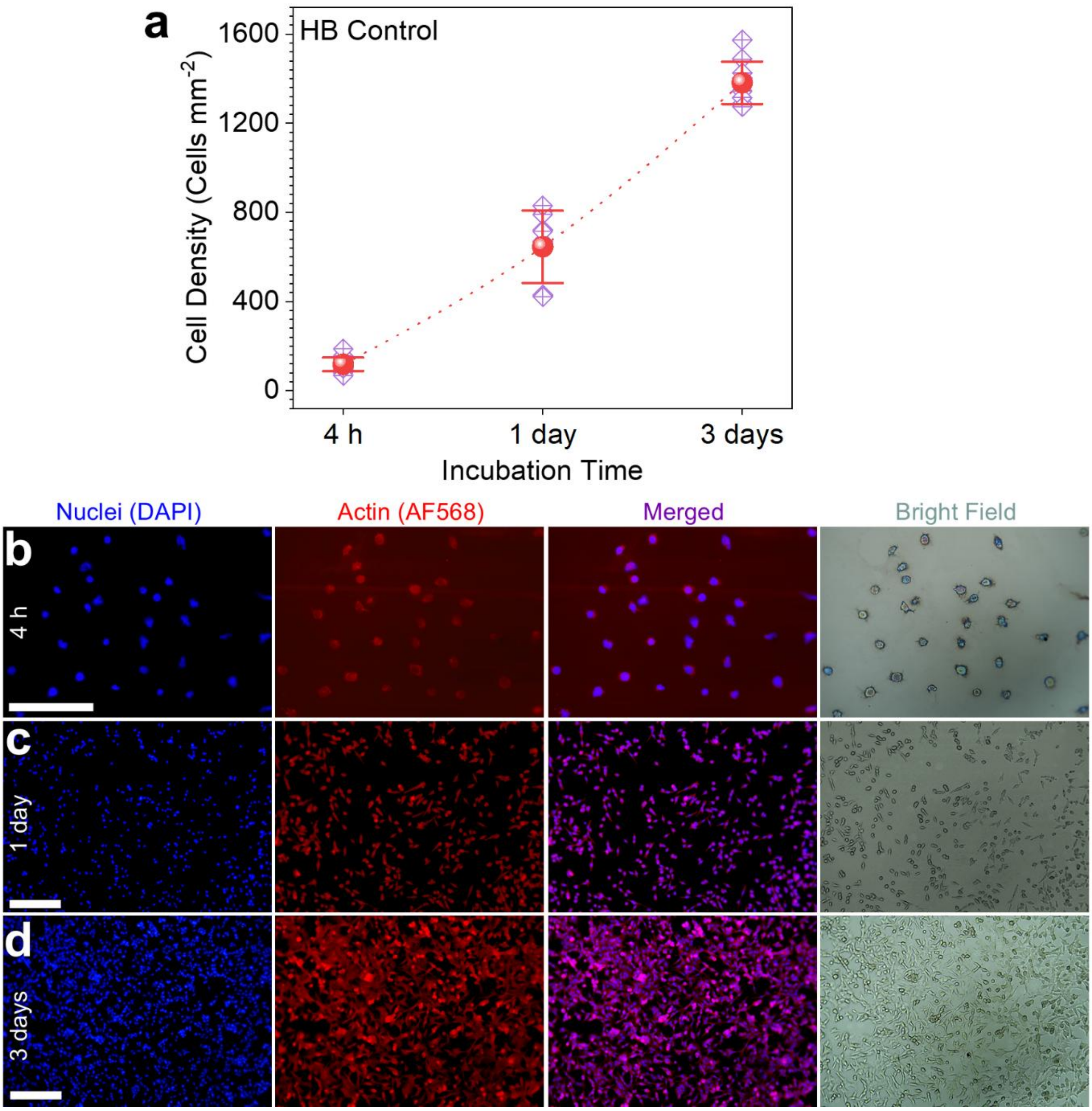

Figure S16. HB control. Cell adhesion and proliferation on the hydrophobic (HB) control surface over 3 days. (a) Cell density as a function of incubation time, showing a steady increase in proliferation. (b–d) Fluorescence microscopy images of nuclei (DAPI, blue) and actin cytoskeleton (AF568, red), with merged and bright-field images, at 4 h, 1 day, and 3 days. Scale bar: 200 µm.

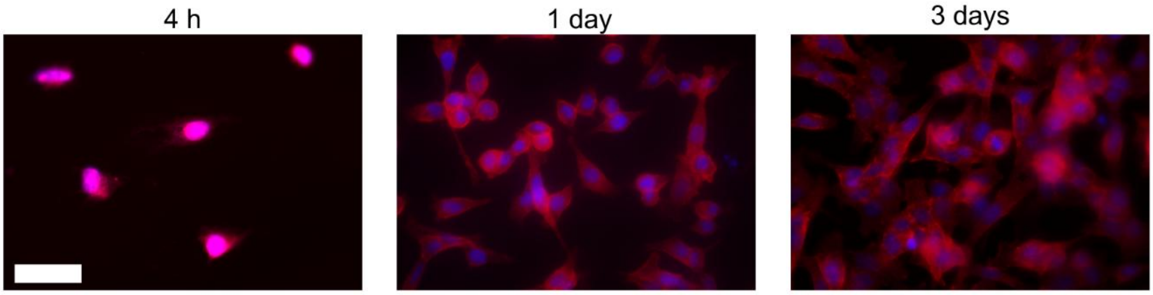

Figure S17. High magnification merged fluorescent images showing cell adhesion and spreading on HB control over time. Scale bar: 50 μm.

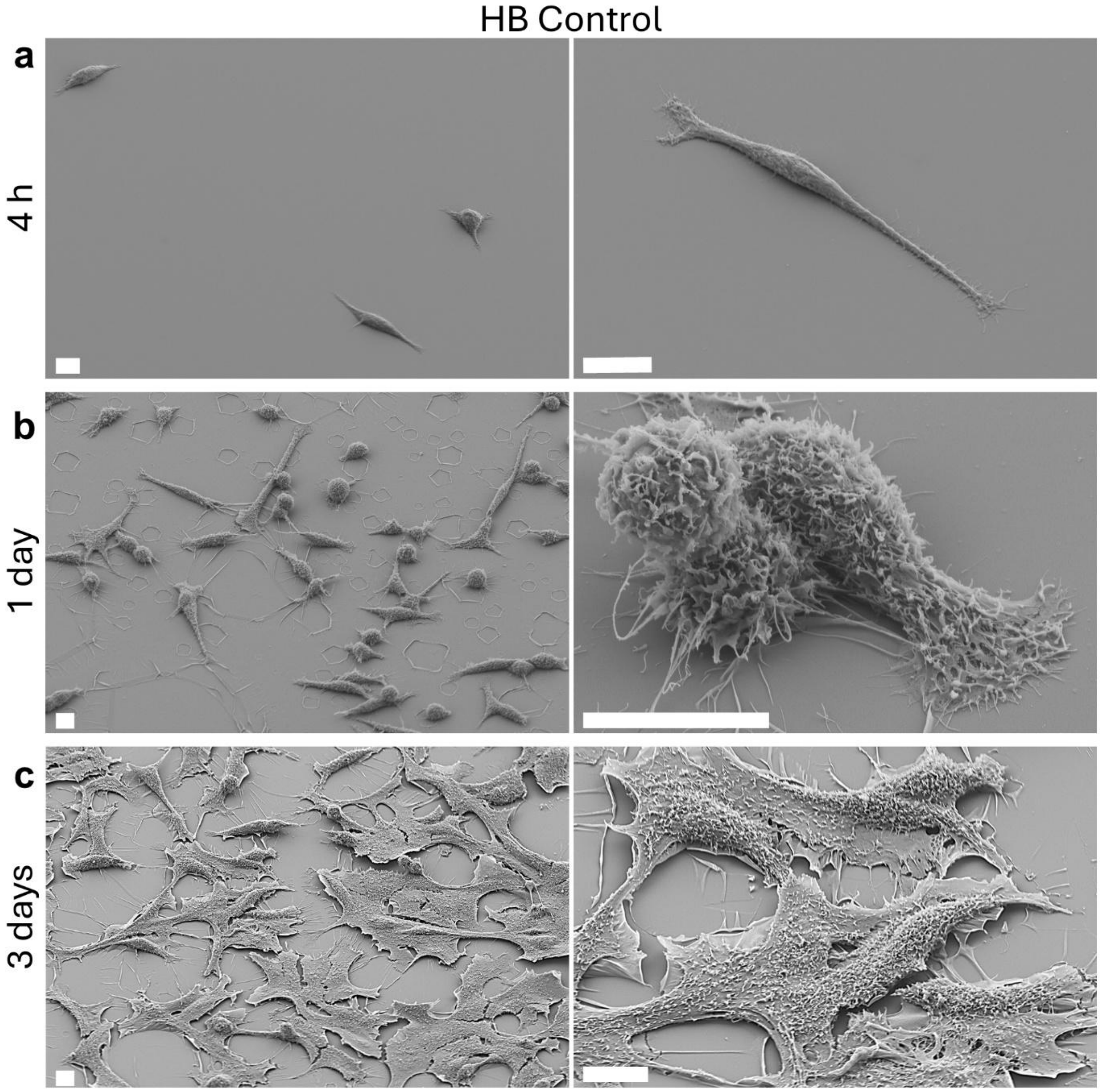

Figure S18. HB control. SEM images of A549 cells cultured on a HB control (IV). Scale bars: 10 μm.

Figure S19 illustrates the temporal progression of A549 cell adhesion and proliferation on superhydrophobic nanopillars over a 3-day incubation period. At 4 hours, minimal adhesion is observed, consistent with the initial low attachment efficiency on superhydrophobic surfaces due to the presence of an air plastron. By 1 day, a slight increase in cell density is detected, indicating the early stages of focal adhesion formation. However, a pronounced transition is observed at 3 days, where a significant increase in cell density occurs, coinciding with a shift from the Cassie–Baxter to Wenzel wetting state. This suggests that as the plastron collapses, cells establish stronger focal adhesion, facilitating rapid proliferation. To further examine this transition, **Figure S20** presents low- and high-magnification fluorescence images of the cells on the superhydrophobic nanopillars at 3 days. The images reveal full confluency across the surface, demonstrating that once the wetting transition occurs, the cells can spread and proliferate efficiently. The bright-field image confirms extensive coverage, with cells forming a continuous monolayer. **Figure S21** provides SEM images capturing the morphological evolution of A549 cells on the superhydrophobic nanopillars at the three time points. At 4 hours, only a few cells exhibit filopodia extensions, indicating weak initial adhesion. By 1 day, more pronounced filopodia and lamellipodia structures are visible, suggesting that cells are actively seeking focal adhesion points. At 3 days, cells appear fully spread with extensive cytoskeletal networks, correlating with the observed proliferation in **Figure S19**. As proteins from the culture medium deposit onto a superhydrophobic nanotexture, they can gradually displace air pockets; the proteins create a more hydrophilic interface and wick liquid into the roughness, destroying the Cassie–Baxter state and increasing cell–solid contact area maximizing cell adhesion and exceeding the control. The transition from minimal adhesion to widespread proliferation highlights the critical role of the air plastron in modulating cell behavior on superhydrophobic surfaces. Roach et al.[20] demonstrated that superhydrophobic porous silica exhibited significantly reduced fibrinogen and albumin adsorption compared to

its hydrophilic counterpart during short exposure times. However, upon the loss of its superhydrophobicity (i.e., plastron dissipation), the surface ultimately exhibited higher overall protein adsorption, suggesting that the presence of a stable air layer is critical in limiting protein interaction with the substrate. Bauer et al.[21] showed that superhydrophobic TiO_2 nanotubes (15–100 nm) initially reduced cell adhesion, but protein adsorption altered surface chemistry, facilitating a wetting transition and enhanced focal adhesion, similar to our Cassie-to-Wenzel transition. Smaller nanotubes promoted adhesion via integrin clustering, while larger ones limited adhesion due to reduced focal contact formation. Ko et al.[22] investigated the adhesion behavior of mouse liver cancer cells on nanostructured superhydrophobic surfaces, revealing that high aspect ratio structures significantly restricted adhesion and proliferation. The cells maintained a spherical morphology with limited focal adhesion sites for up to three days, demonstrating that surface topography plays a crucial role in suppressing cell attachment. Marcon et al.[15] observed, when studying cell adhesion on diamond nanowires, that despite the nanoscale roughness of the nanowires, cells did not spread across the superhydrophobic regions and preferred the superhydrophilic one even after 72 h of incubation, likely due to the persistent air layer acting as a physical barrier, preventing direct cell–surface interactions. Lim et al.[23] observed that nanoisland topography influenced human fetal osteoblastic cell adhesion and morphology, with 11 nm high islands promoting enhanced cell spreading, focal adhesion formation, and proliferation compared to 38 nm and 85 nm islands. Piret et al.[17] observed that superhydrophobic silicon nanowire surfaces exhibited a strong nonfouling effect, preventing CHO cell adhesion due to a stable Cassie–Baxter state that minimized contact with the culture mediumCulture of mammalian. This behavior aligns with the high cell repellency observed on superhydrophobic nanopillars in our study, where the maintenance of an air plastron similarly inhibited adhesion by limiting direct contact between the cells and the substrate and cells filopodia remained on the tips of the nanowires and did not penetrate into the gaps.

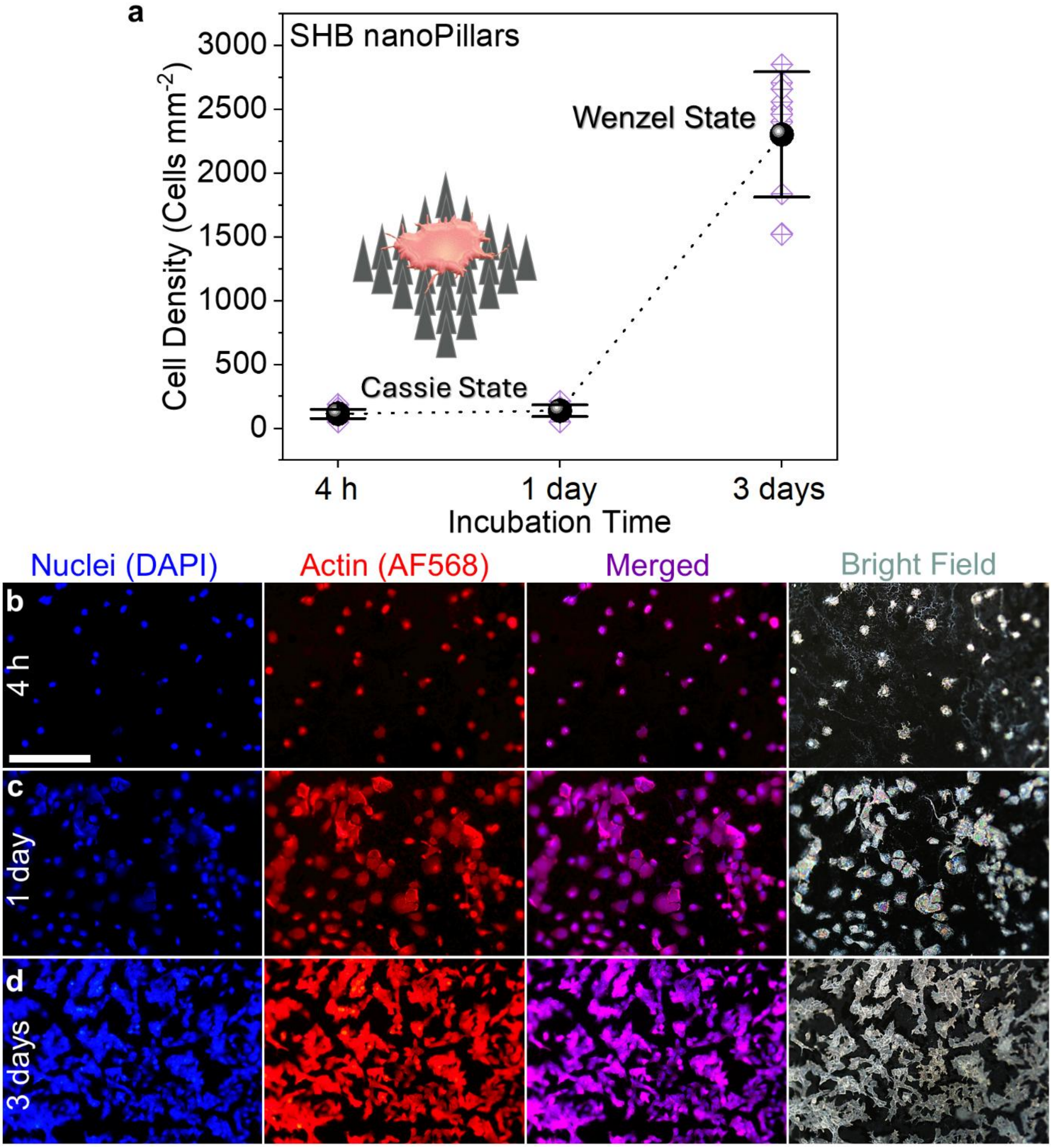

Figure S19. Superhydrophobic (SHB) nanopillars. Cell adhesion and proliferation on the superhydrophobic nanopillared surface over 3 days. a) Cell density as a function of incubation time. b–d) Fluorescence microscopy images of nuclei (DAPI, blue) and actin cytoskeleton (AF568, red), with merged and bright-field images, at 4 h, 1 day, and 3 days. Scale bar: 200 µm.

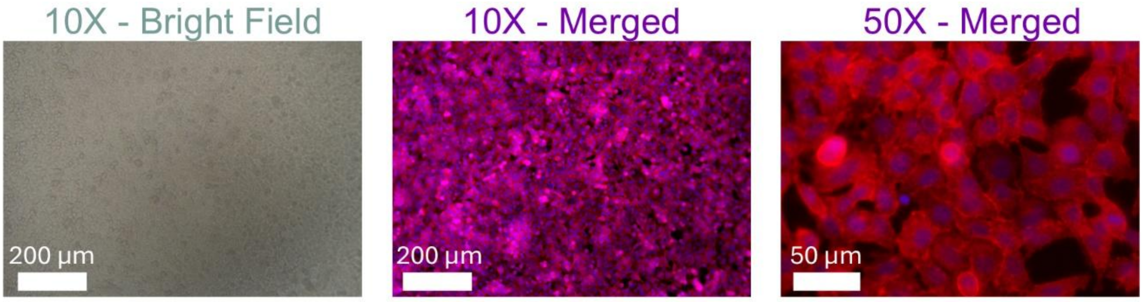

Figure S20. Low and high magnification merged fluorescent images showing full confluency on superhydrophobic nanopillars after 3 days of incubation. Scale bars: 200 µm and 50 µm.

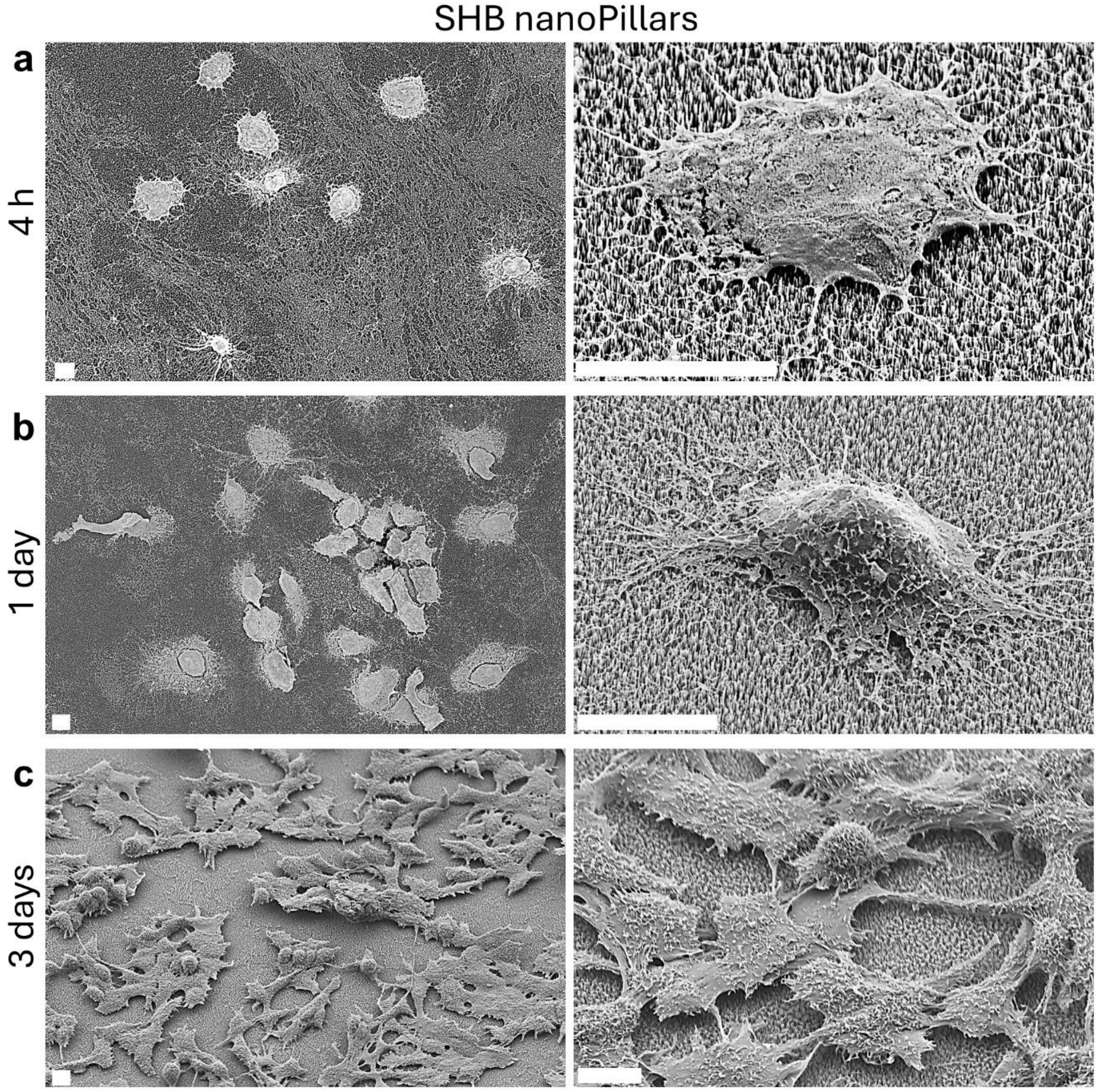

Figure S21. Superhydrophobic nanopillars. SEM images of A549 cells cultured on superhydrophobic nanopillars (V). Scale bars: 10 µm.

S4 Micropillar Solid Fraction Effect on Cell Adhesion

The 22.7% solid fraction exhibited higher cell adhesion than the smooth control, likely because the absence of surface topography on the control weakened adhesion by inducing protein conformational changes that reduced biological activity.[14] Additionally, the smooth surface lacked mechanical anchoring sites, further limiting cell attachment after 4 h of incubation.

At the Wenzel state, cell spreading is enhanced at low solid fraction due to less pillars that form obstacles for cell focal adhesion. Our group has studied the stability of micropillars air plastron upon immersion in biofluids.[9] It was revealed that higher solid fractions and smaller pillar sizes lead to a stable biofluid repellency, while lower solid fraction led to a greater but short-lived biofluid repellency. There are a few studies that studied cell adhesion on microstructured surfaces. Bian et al.[24] investigated the role of micropillar arrays in modulating cell adhesion, demonstrating that patterned surfaces with 20 µm-high square pillars significantly influenced cellular interactions. The study revealed that smaller pillar spacings (5 µm) enhanced pseudopod extension and cell attachment, whereas larger spacings (10–20 µm) created cell-trapping effects due to the interplay between surface roughness and wettability. Zhang et al.[25] fabricated a micropatterned superhydrophobic surface on titanium using ultraviolet laser processing, ultrasonic acid treatment, and chemical modification. Their structure consisted of a regular microgrid with tunable dimensions, optimizing air entrapment and reducing blood contact, which effectively minimized thrombosis formation in vivo. De Vitis et al.[26] fabricated silicon micropillars with an aspect ratio greater than 2, designed to investigate MCF-7 cell adhesion in confined microenvironments. Their study used surface enhanced Raman spectroscopy and revealed that cells primarily adhered to the pillar tops and extended protrusions to span across adjacent pillars aligning with our observations. Accardo et al.[27] fabricated biomimetic polymeric surfaces consisting of SU-8 micropillars and nanofibrillar

polymethylmethacrylate (PMMA) to investigate colon cancer cell adhesion. Their study revealed that HCT116 cells formed stable interconnections with the microstructured surfaces, with filopodia exerting mechanical forces on the SU-8 micropillars, leading to their partial bending. Flamourakis et al.[2] fabricated nanopillar arrays using two-photon polymerization (2PP) with a tunable effective shear modulus to guide neuronal network directionality. The nanopillars, with diameters of 500 nm and heights ranging from 0.6 to 5 μm, influenced neuronal differentiation and neurite alignment, mimicking extracellular matrix cues. Ghalandarzadeh et al.[28] demonstrated that microtextured zirconia surfaces fabricated via laser ablation exhibited significantly reduced Staphylococcus aureus adhesion compared to non-textured surfaces, with a hydrophobicity-driven antibacterial effect. While both micro-grooved and micro-channelled surfaces hindered bacterial colonization, the latter achieved a slightly higher antibacterial rate, emphasizing the role of microstructure geometry in modulating bacterial interactions.

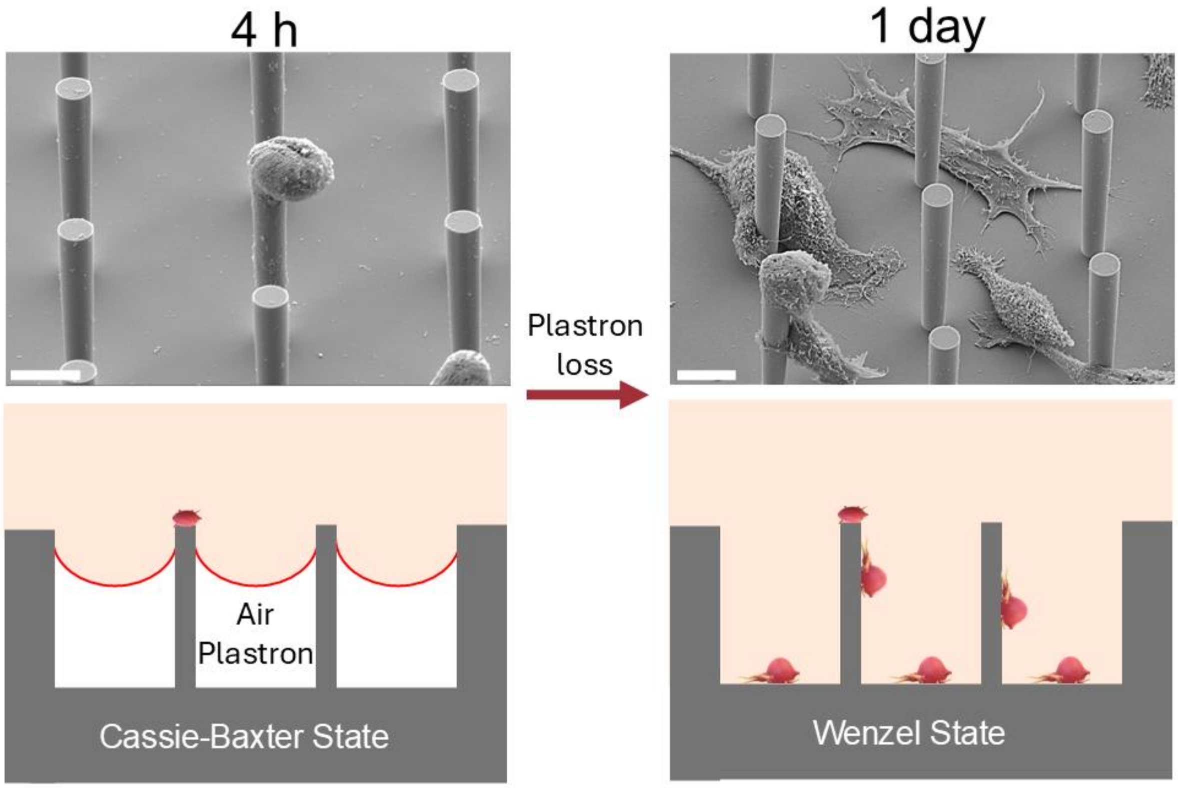

Figure S22. SEM images and schematic representation of the wetting transition from the Cassie–Baxter to Wenzel state on superhydrophobic 5 µm pillars with 2.5% solid fraction. Scale bars: 10 µm.

S5. Long-Term Influence of Pillar Size on Cell Adhesion

Figures S23–S30 show the pillar size effect at early incubation timepoint (4 h). At extended incubation times (1 and 3 days), the role of micropillar diameter in modulating cell adhesion became increasingly governed by wetting state transitions and cell proliferation dynamics. At 22.7% and 14.5% solid fractions, surfaces with 5–10 μm pillars maintained stable plastrons over 24 h, resulting in relatively uniform but moderate cell adhesion across pillar sizes. Notably, 20 μm pillars supported more evenly distributed cells after 1 day (**Figures S31–S33**, **S34–S36**), suggesting that larger top surfaces and accessible spacing facilitated later-stage adhesion once plastron effects diminished. By 3 days (**Figures S43–S45**, **S46–S48**), all high-solid fraction samples approached confluency, but cells on smaller pillars exhibited more interconnected spreading, while those on larger pillars remained slightly sparser.

At 7.4% and 2.5% solid fractions, pillar size had a stronger effect due to varying degrees of plastron collapse. After 1 day, 20 μm pillars at both fractions had transitioned to the Wenzel state, allowing significant cell attachment (**Figures S37–S39**, **S40–S42**). In contrast, 5 and 10 μm pillars partially retained their air plastrons, particularly at 7.4%, and thus limited adhesion. By 3 days, all surfaces, regardless of diameter, showed extensive cell coverage (**Figures S49–S54**), reflecting full plastron loss and the dominant effect of cell proliferation. SEM revealed that cells bridged across even the widest pillar gaps on 2.5%/20 μm surfaces, forming cohesive networks.

Overall, while small-diameter pillars suppress early adhesion by preserving the Cassie–Baxter state and limiting cell bridging, their long-term advantage depends on sustaining the air layer. Once wetting transitions occur, large-diameter, low-density structures offer broad access to the substrate, facilitating rapid colonization. Over time, cell proliferation increases adhesion on all surfaces, but topographical and wetting effects remain dominant. Overall, cell adhesion on all superhydrophobic micropillared surfaces was significantly lower than on superhydrophobic nanopillars, and hydrophobic and hydrophilic controls (**Figure 3**). These results emphasize that pillar diameter must be tuned not only for initial repellency but also for long-term stability against wetting and fouling.

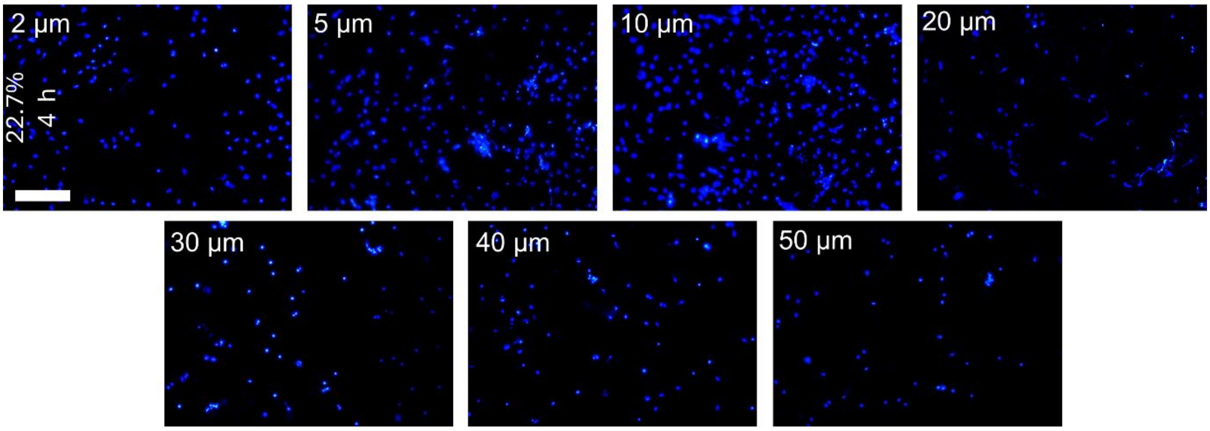

Figure S23. Low-magnification fluorescence microscopy images of cell adhesion on micropillar surfaces with varying pillar sizes (2–50 μm) with 22.7% solid fraction after 4 h of incubation. Scale bar: 200 μm.

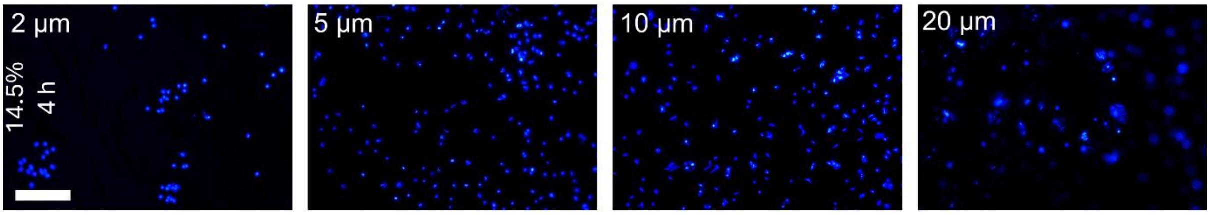

Figure S24. Low-magnification fluorescence microscopy images of cell adhesion on micropillar surfaces with varying pillar sizes (2–20 μm) with 14.5% solid fraction after 4 h of incubation. Scale bar: 200 μm.

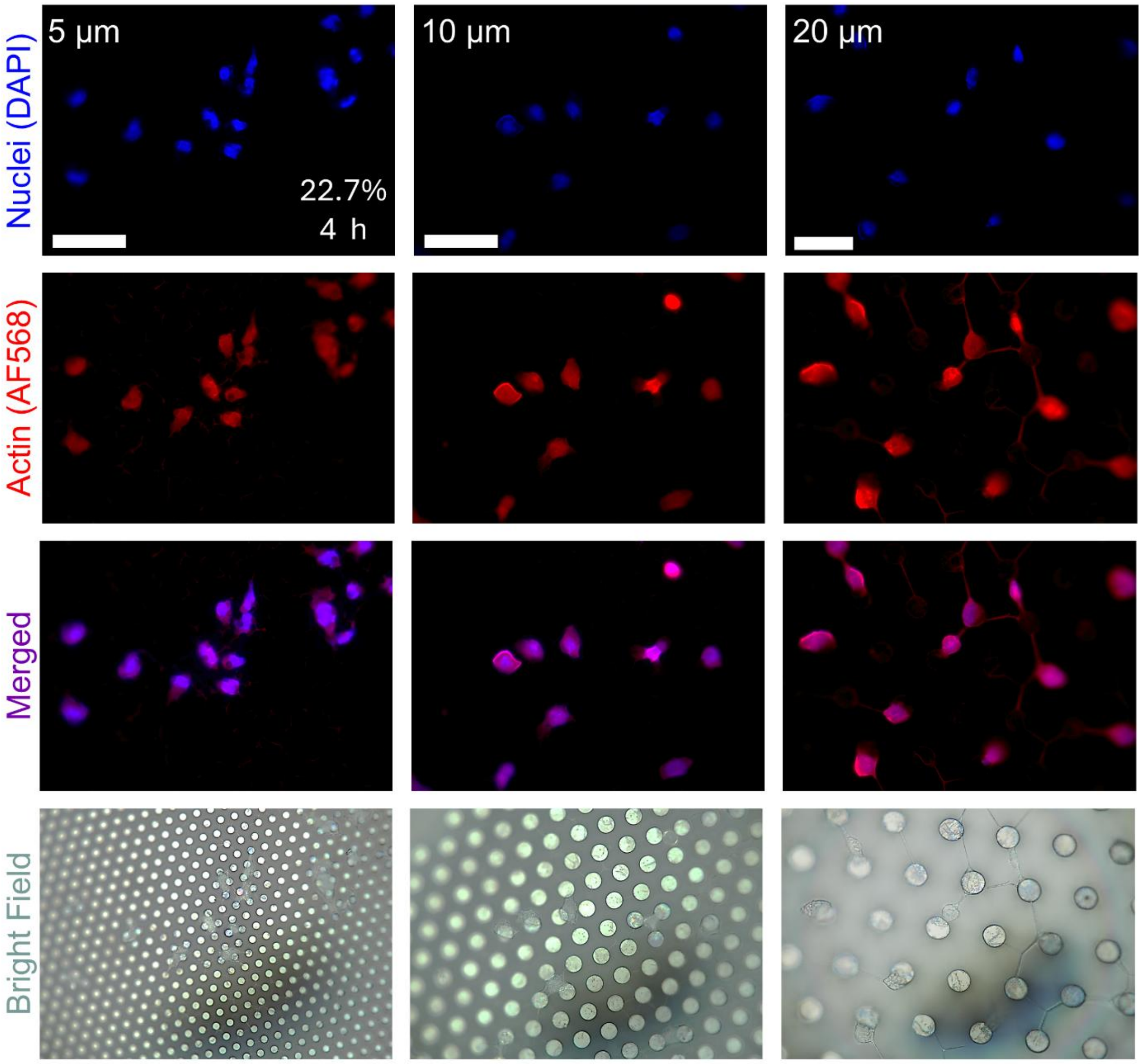

Figure S25. High-magnification fluorescence and bright field images displaying adhered cells on micropillar surfaces with varying pillar sizes (5, 10, and 20 µm) at 22.7% after 4 h of incubation showing nuclei (DAPI, blue), actin cytoskeleton (AF568, red), and merged channels. Scale bars: 50 µm.

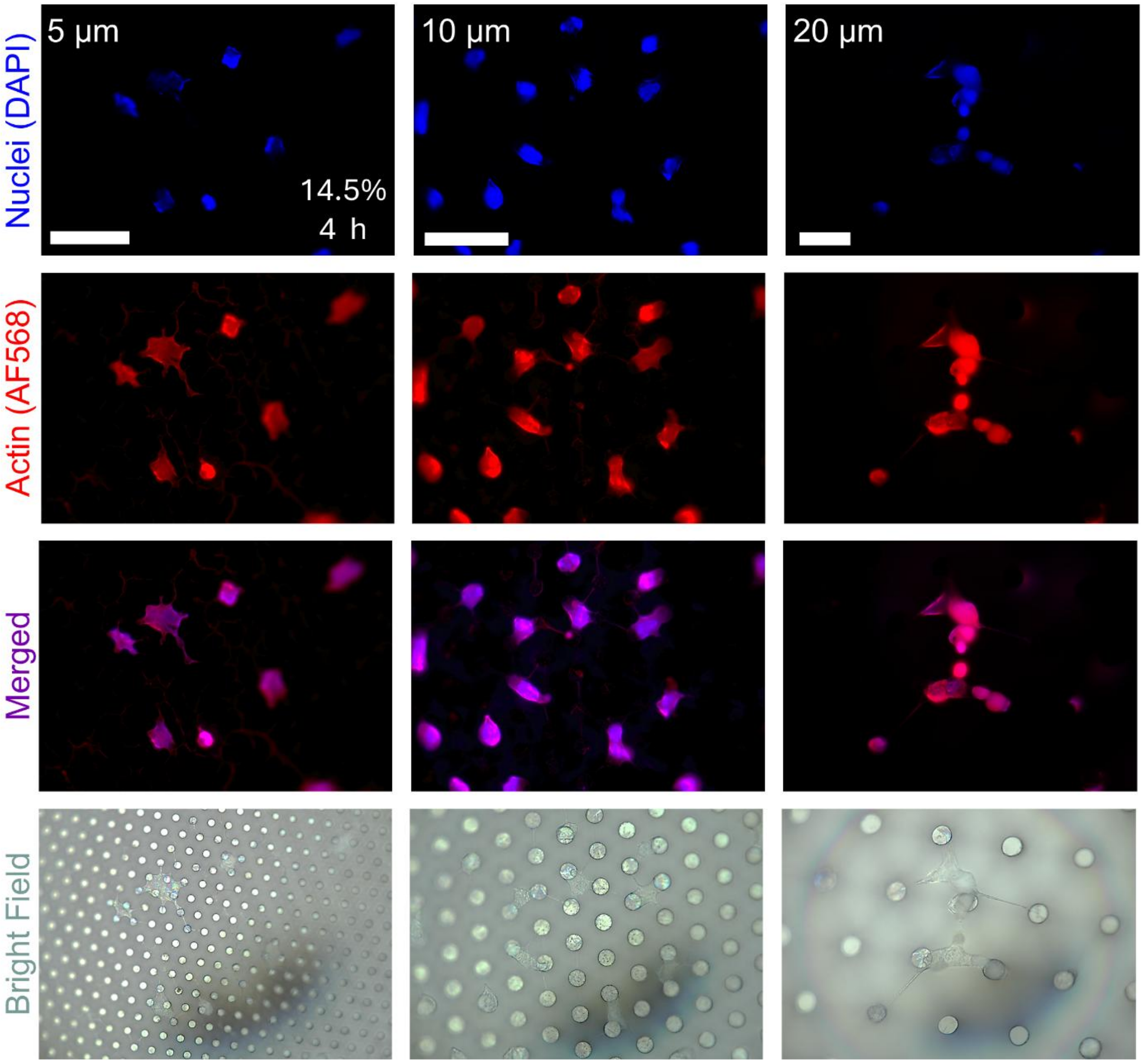

Figure S26. High-magnification fluorescence and bright field images displaying adhered cells on micropillar surfaces with varying pillar sizes (5, 10, and 20 µm) at 14.5% after 4 h of incubation showing nuclei (DAPI, blue), actin cytoskeleton (AF568, red), and merged channels. Scale bars: 50 µm.

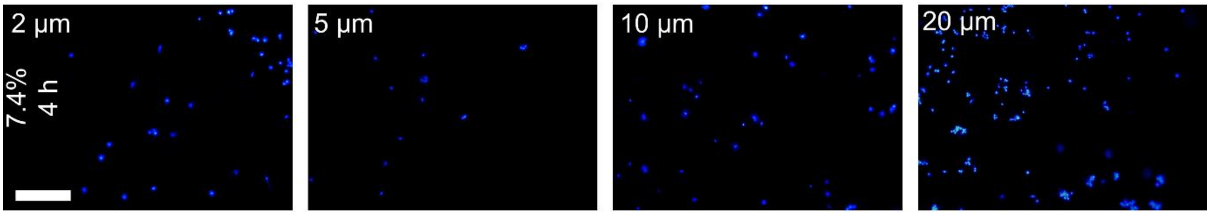

Figure S27. Low-magnification fluorescence images of cell adhesion on micropillar surfaces with varying pillar sizes (2–20 µm) with 7.4% solid fraction after 4 h of incubation. Scale bar: 200 µm.

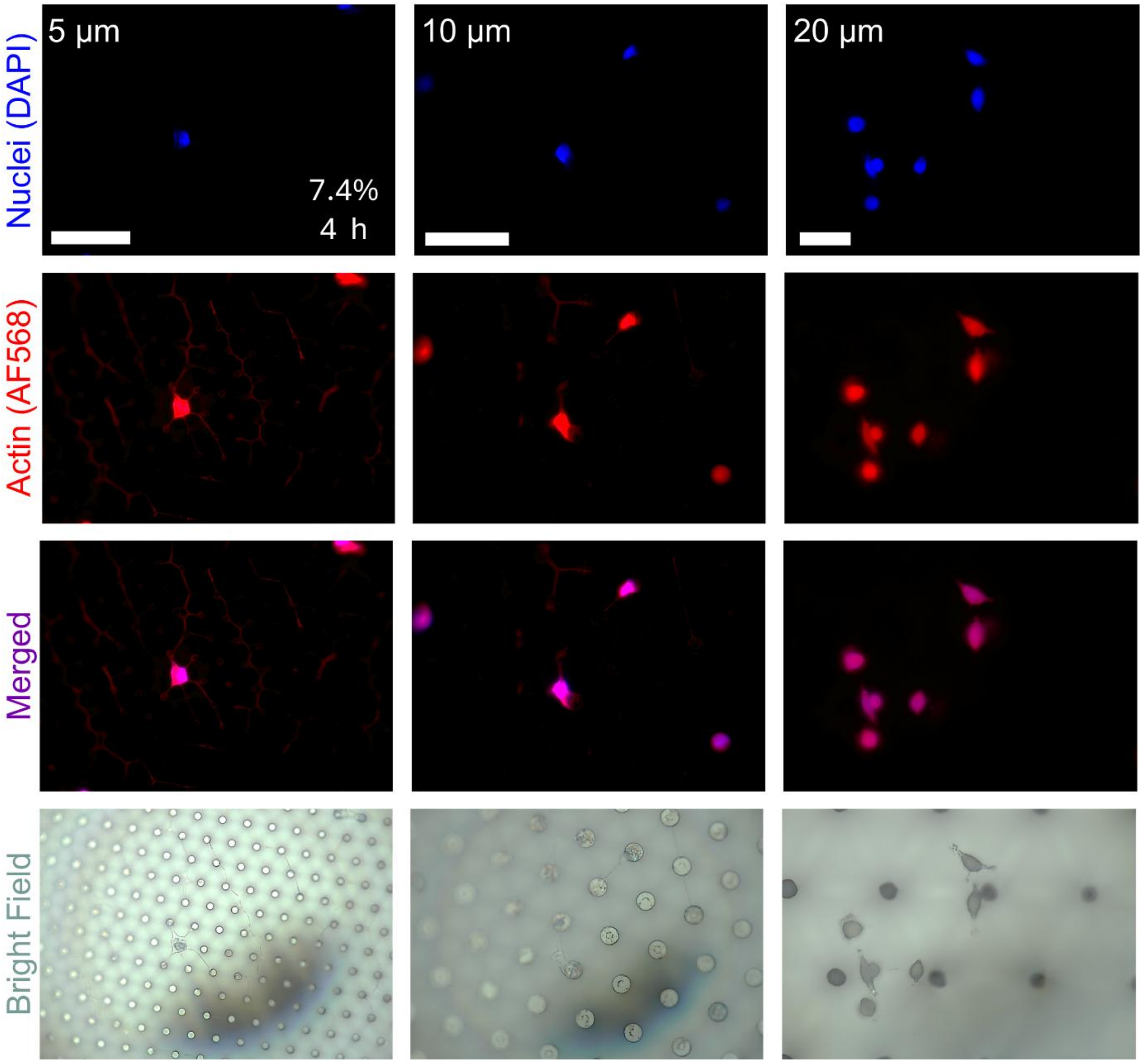

Figure S28. High-magnification fluorescence and bright field images displaying adhered cells on micropillar surfaces with varying pillar sizes (5, 10, and 20 µm) at 7.4% after 4 h of incubation showing nuclei (DAPI, blue), actin cytoskeleton (AF568, red), and merged channels. Scale bars: 50 µm.

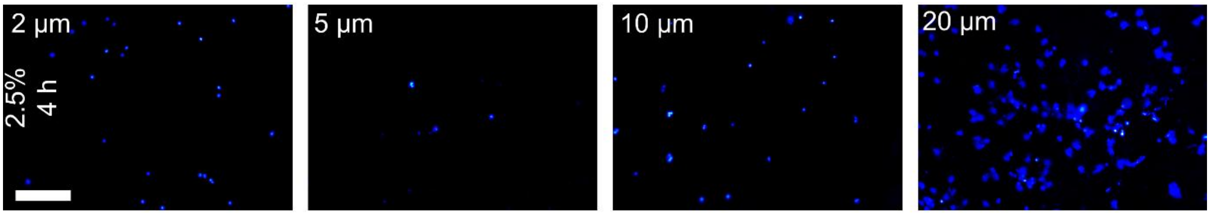

Figure S29. Low-magnification fluorescence microscopy images of cell adhesion on micropillar surfaces with varying pillar sizes (2–20 µm) with 2.5% solid fraction after 4 h of incubation. Scale bar: 200 µm.

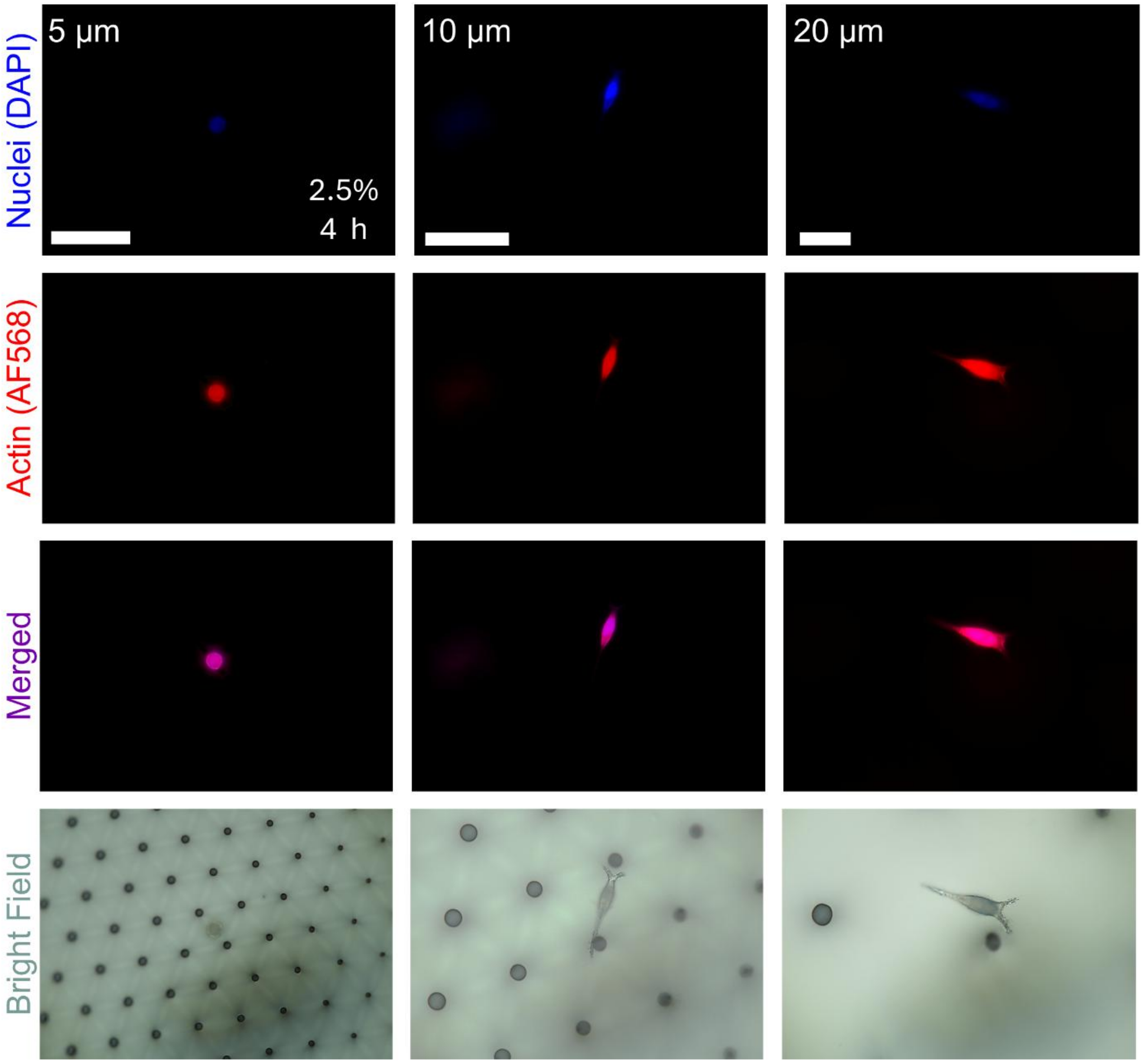

Figure S30. High-magnification fluorescence and bright field images displaying adhered cells on micropillar surfaces with varying pillar sizes (5, 10, and 20 µm) at 2.5% after 4 h of incubation showing nuclei (DAPI, blue), actin cytoskeleton (AF568, red), and merged channels. Scale bars: 50 µm.

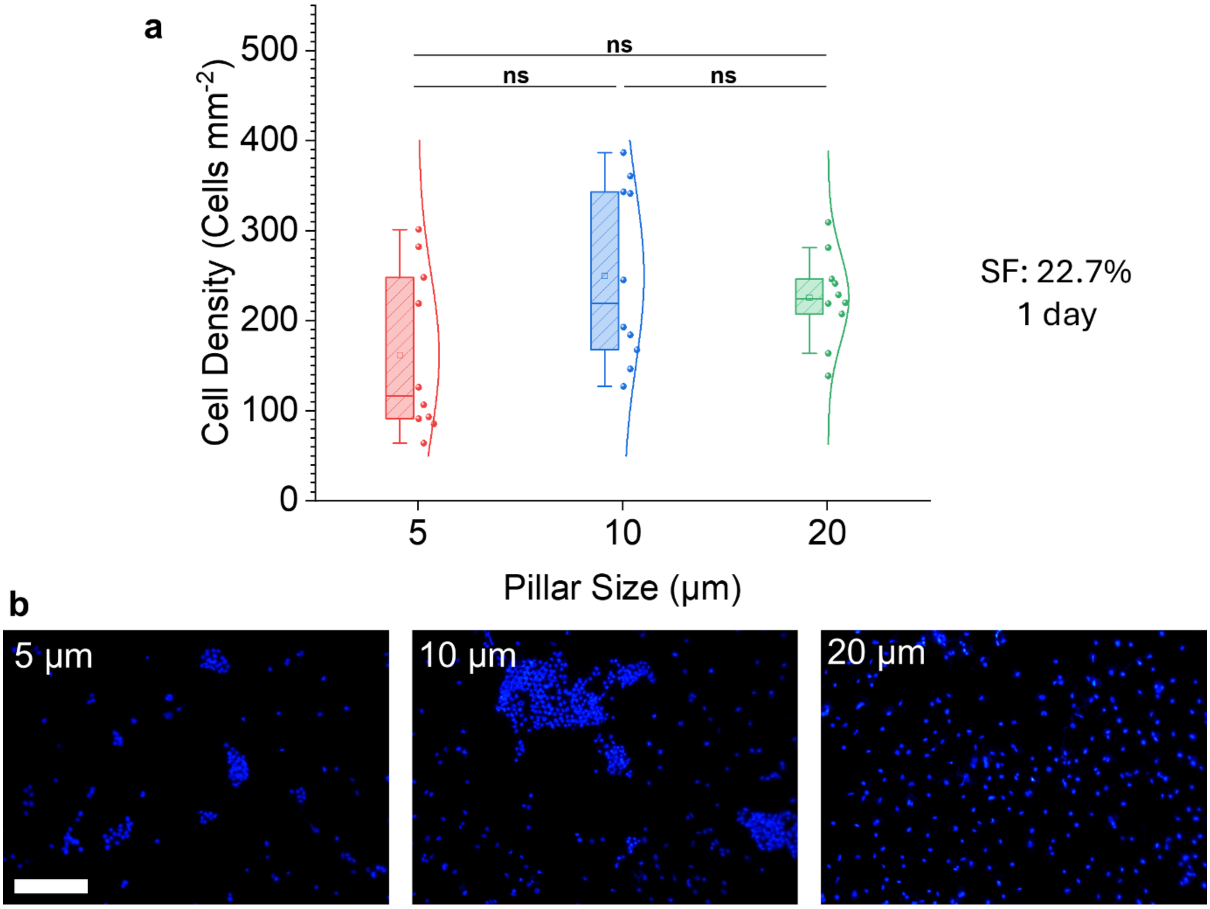

Figure S31. Influence of pillar size at 22.7% solid fraction on cell adhesion and morphology after 1 day of incubation. **a**) Cell density as a function of pillar size for surfaces. **b**) Low-magnification fluorescence microscopy images of cell adhesion on the micropillared surfaces. Scale bar: 200 μm.

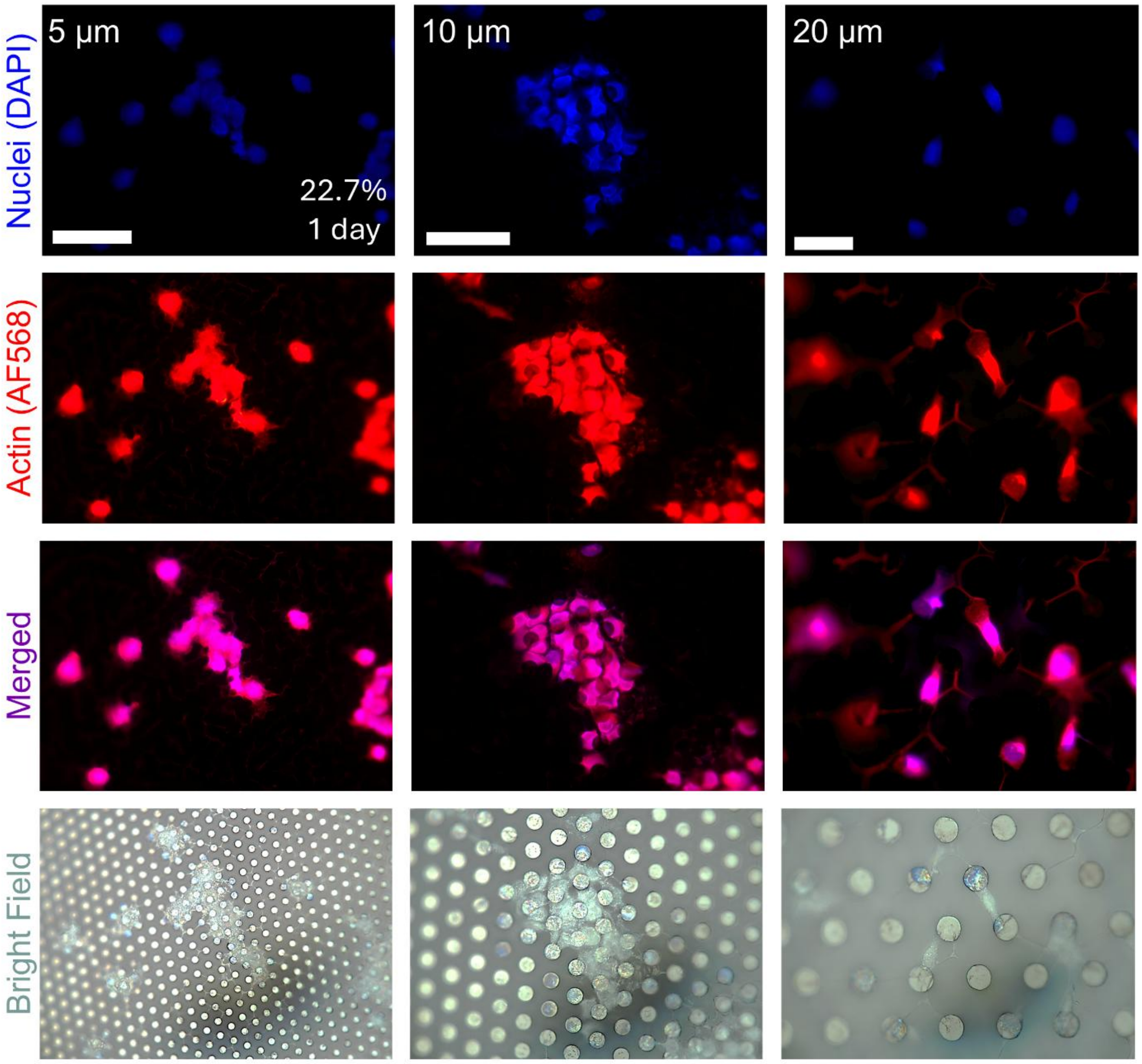

Figure S32. High-magnification fluorescence and bright field images displaying adhered cells on micropillar surfaces with varying pillar sizes (5, 10, and 20 µm) with 22.7% solid fraction after 1 day of incubation showing nuclei (DAPI, blue), actin cytoskeleton (AF568, red), and merged channels. Scale bars: 50 µm.

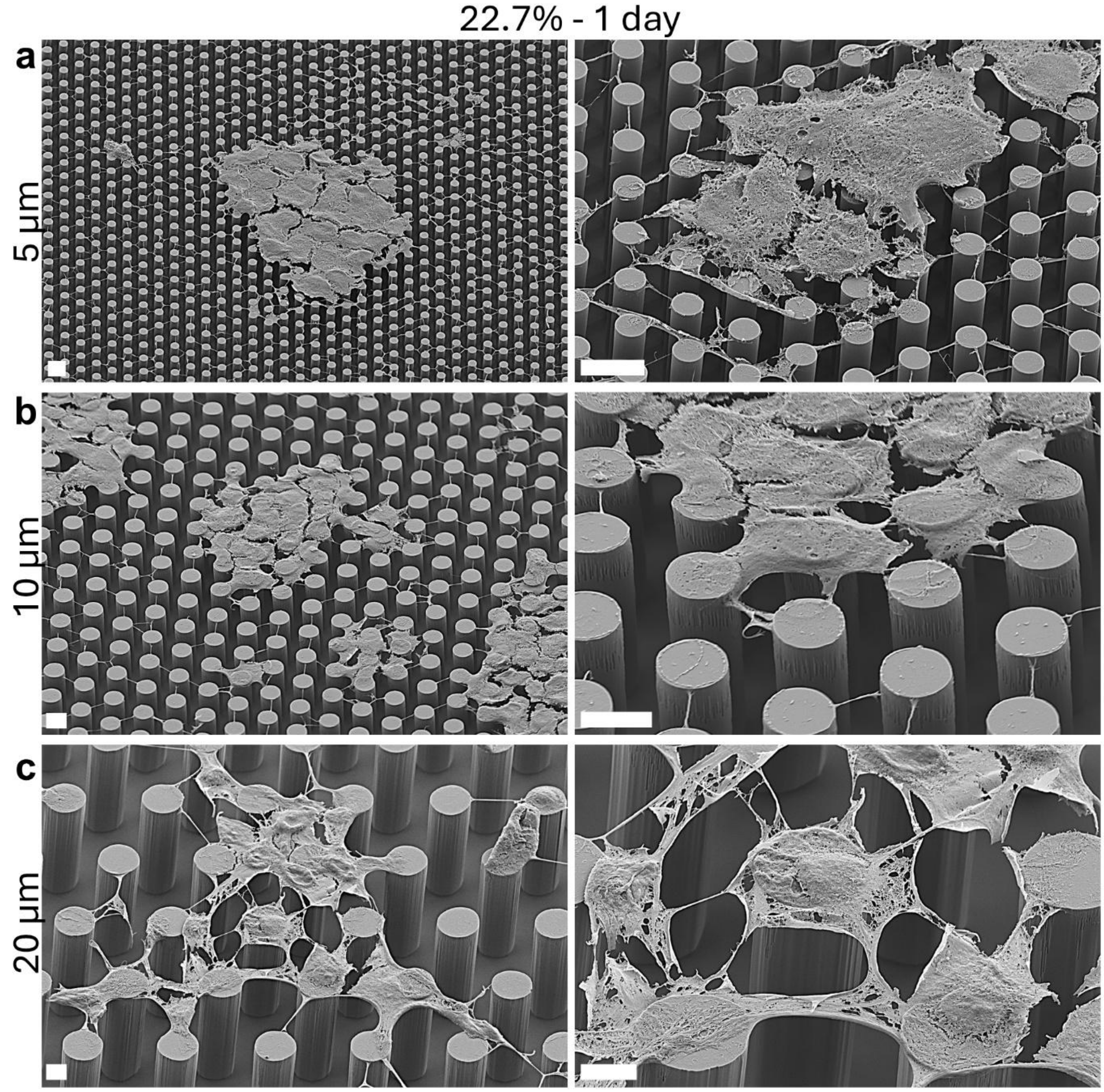

Figure S33. SEM images of superhydrophobic micropillared surfaces with 22.7% solid fraction after 1 day of cell incubation. **a–c**) Low (left) and high (right) magnification SEM images of 5 µm, 10 µm, and 20 µm pillar sizes, respectively. Scale bars: 10 µm.

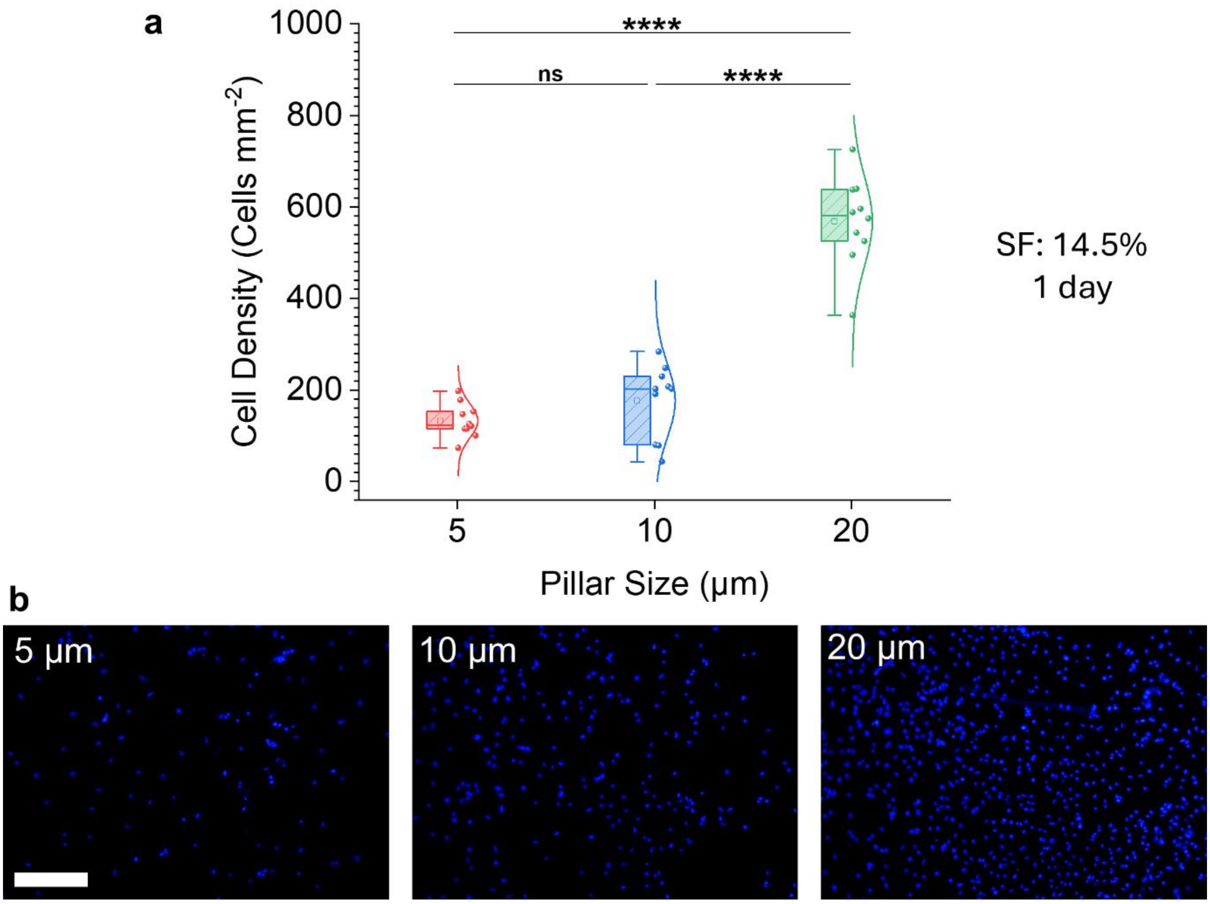

Figure S34. Influence of pillar size at 14.5% solid fraction on cell adhesion and morphology after 1 day of incubation. **a**) Cell density as a function of pillar size for surfaces. **b**) Low-magnification fluorescence microscopy images of cell adhesion on the micropillared surfaces. Scale bar: 200 µm.

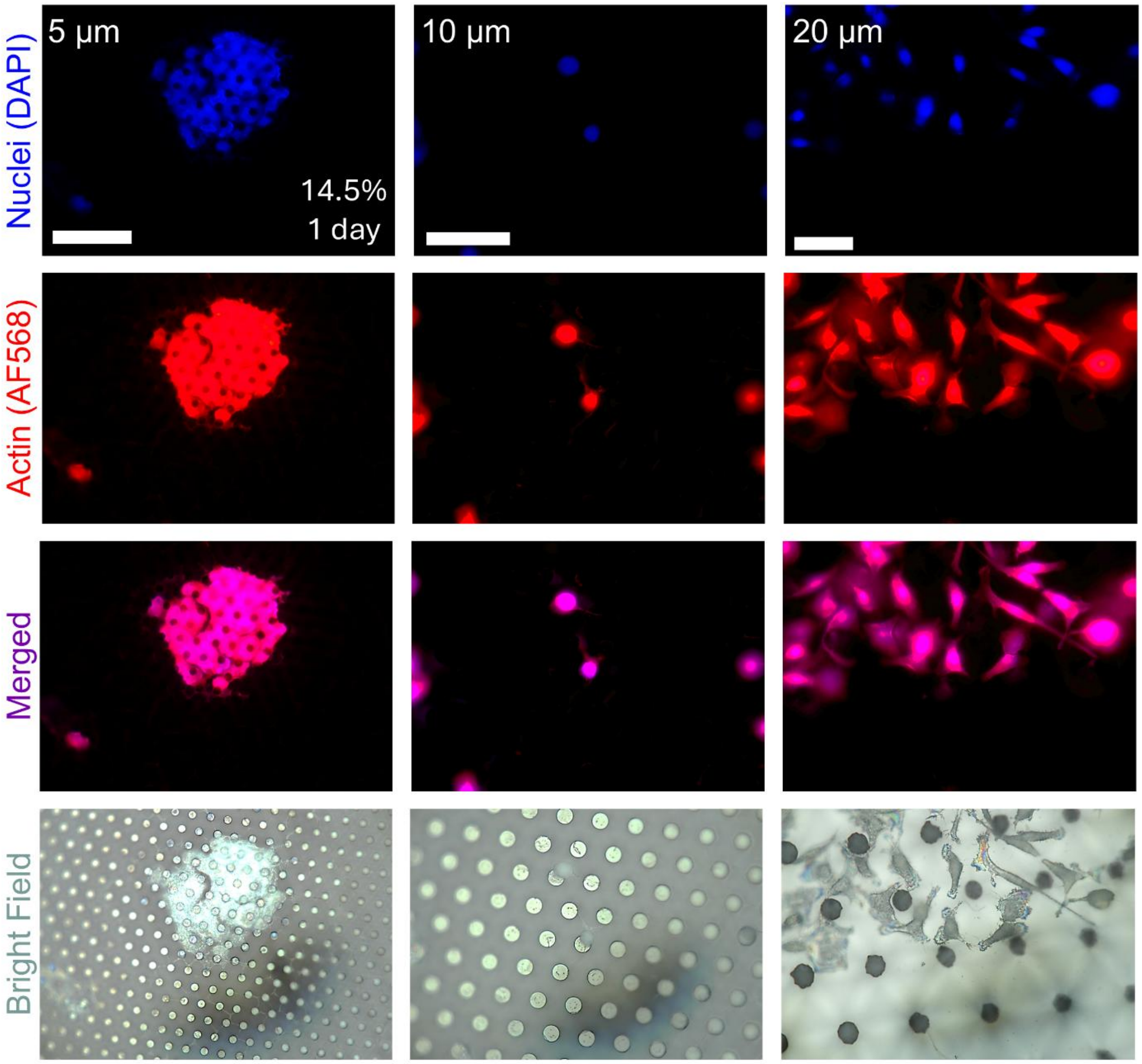

Figure S35. High-magnification fluorescence and bright field images displaying adhered cells on micropillar surfaces with varying pillar sizes (5, 10, and 20 µm) with 14.5% solid fraction after 1 day of incubation showing nuclei (DAPI, blue), actin cytoskeleton (AF568, red), and merged channels. Scale bars: 50 µm.

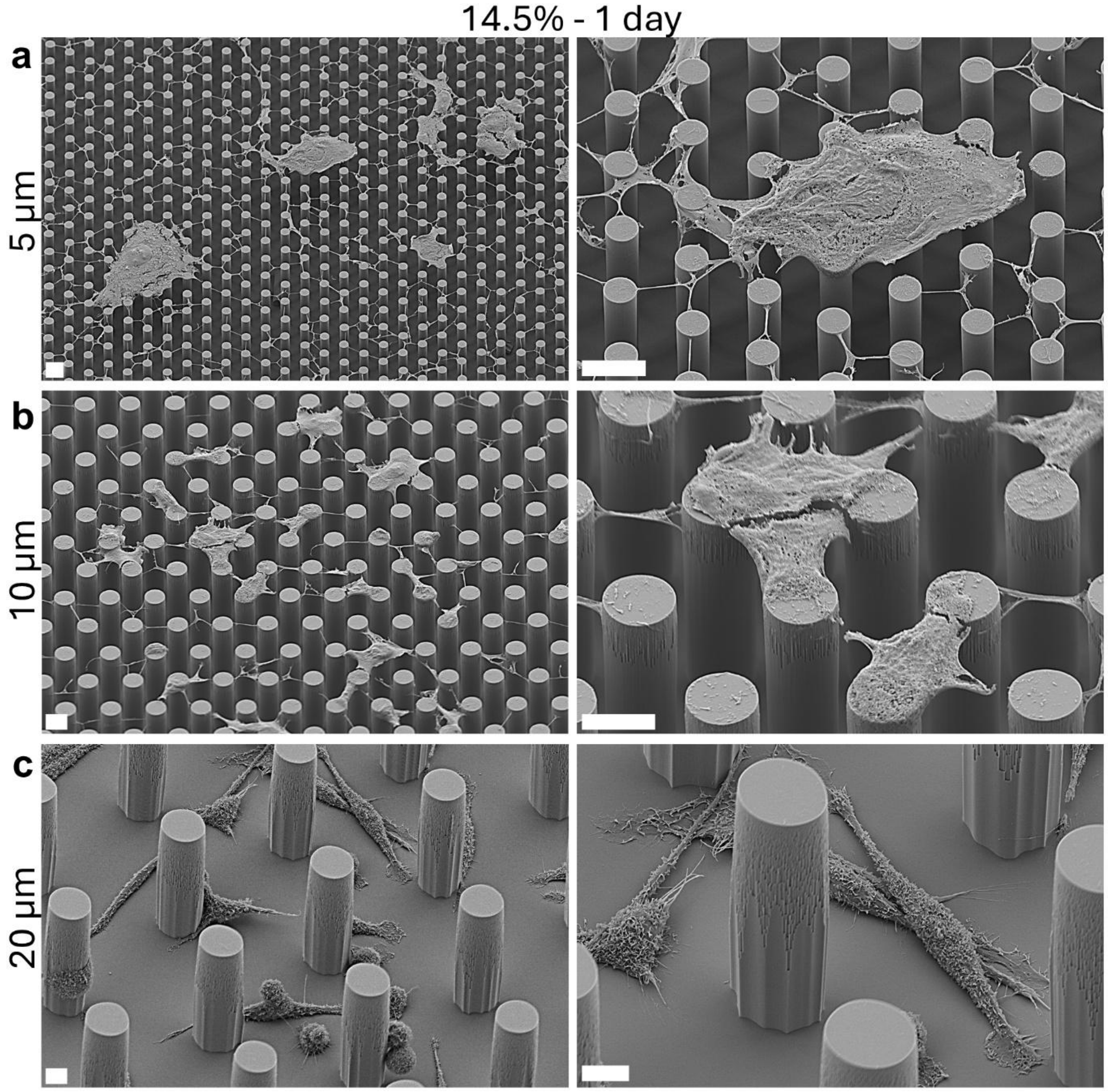

Figure S36. SEM images of superhydrophobic micropillared surfaces with 14.5% solid fraction after 1 day of cell incubation. **a–c**) Low (left) and high (right) magnification SEM images of 5 µm, 10 µm, and 20 µm pillar sizes, respectively. Scale bars: 10 µm.

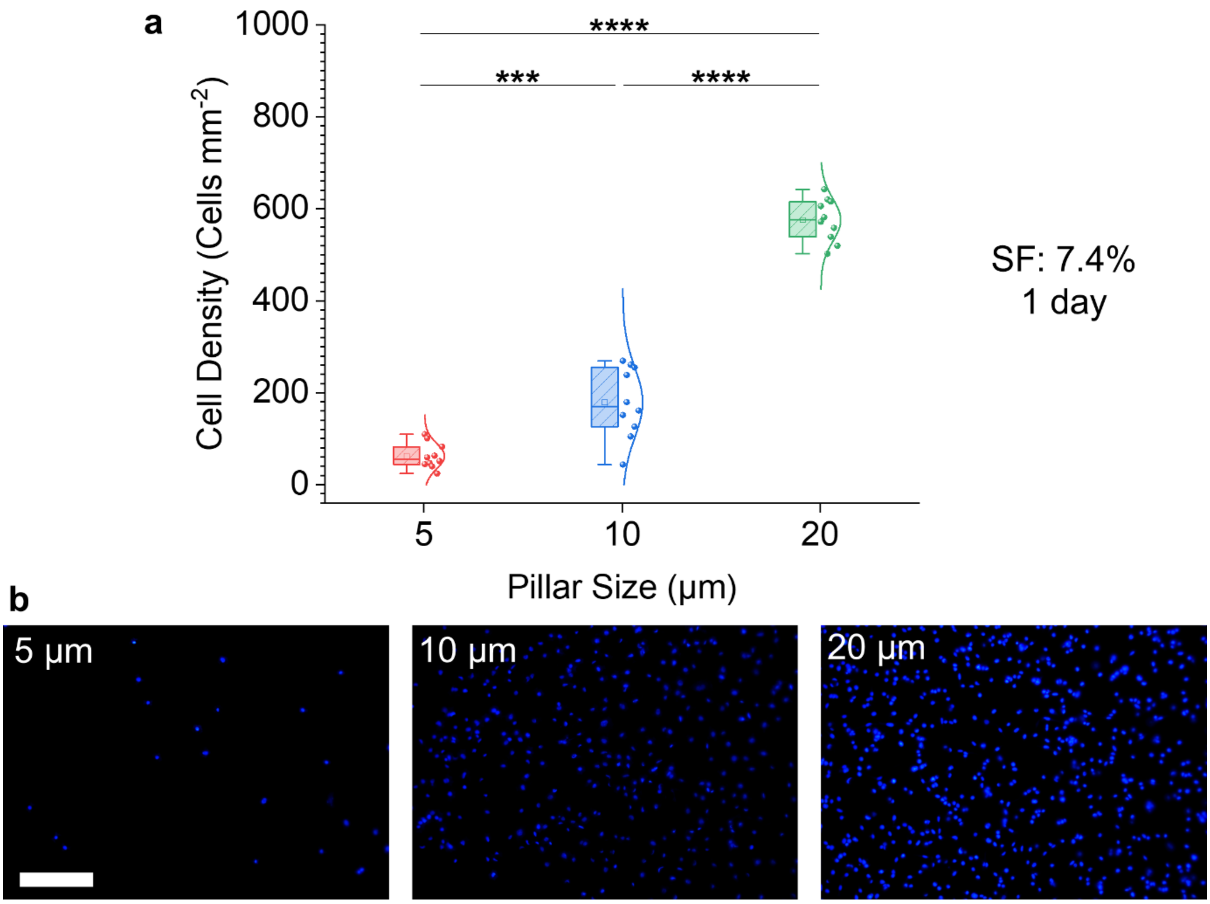

Figure S37. Influence of pillar size at 7.4% solid fraction on cell adhesion and morphology after 1 day of incubation. **a**) Cell density as a function of pillar size for surfaces. **b**) Low-magnification fluorescence microscopy images of cell adhesion on the micropillared surfaces. Scale bar: 200 µm.

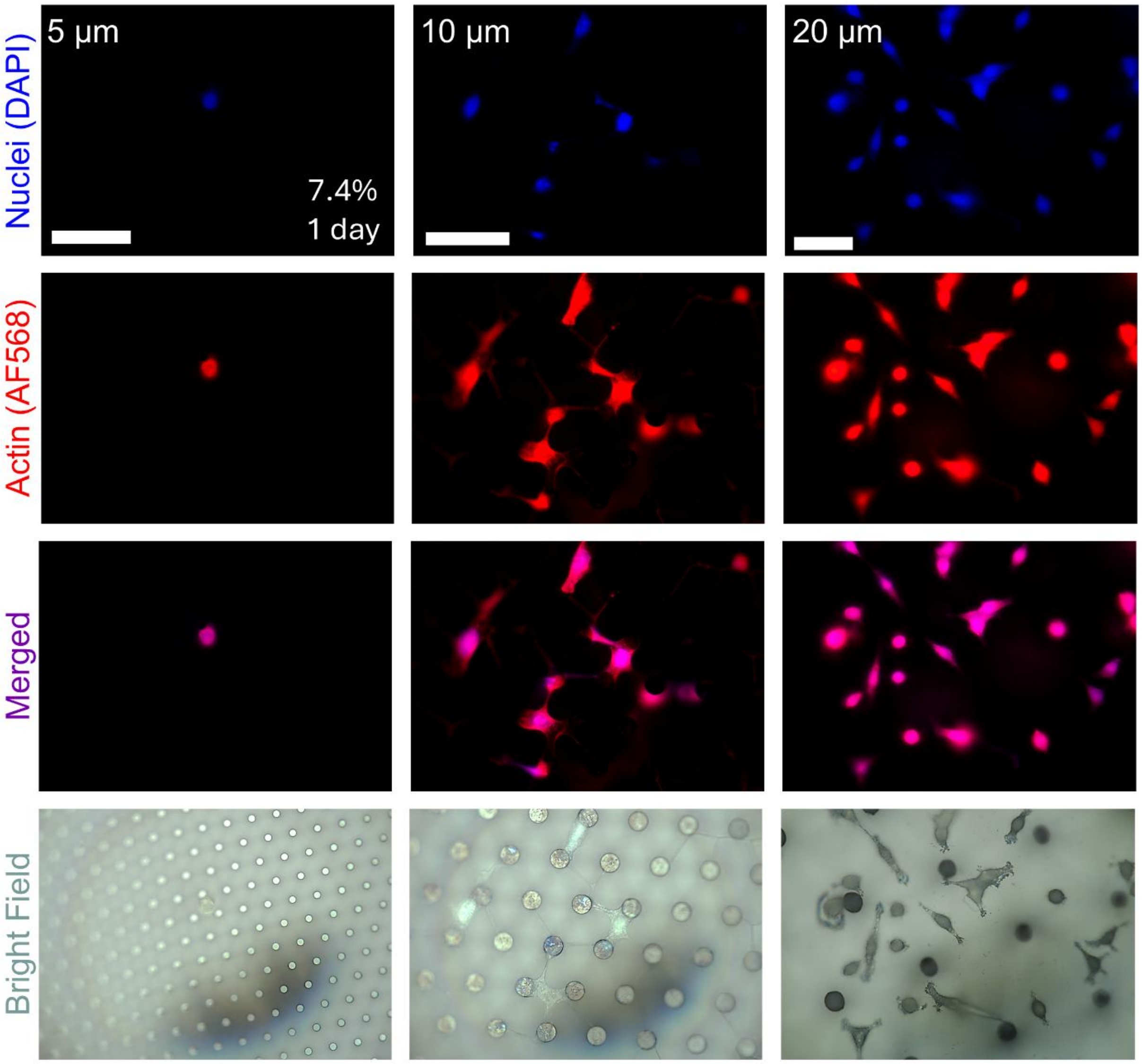

Figure S38. High-magnification fluorescence and bright field images displaying adhered cells on micropillar surfaces with varying pillar sizes (5, 10, and 20 µm) with 7.4% solid fraction after 1 day of incubation showing nuclei (DAPI, blue), actin cytoskeleton (AF568, red), and merged channels. Scale bars: 50 µm.

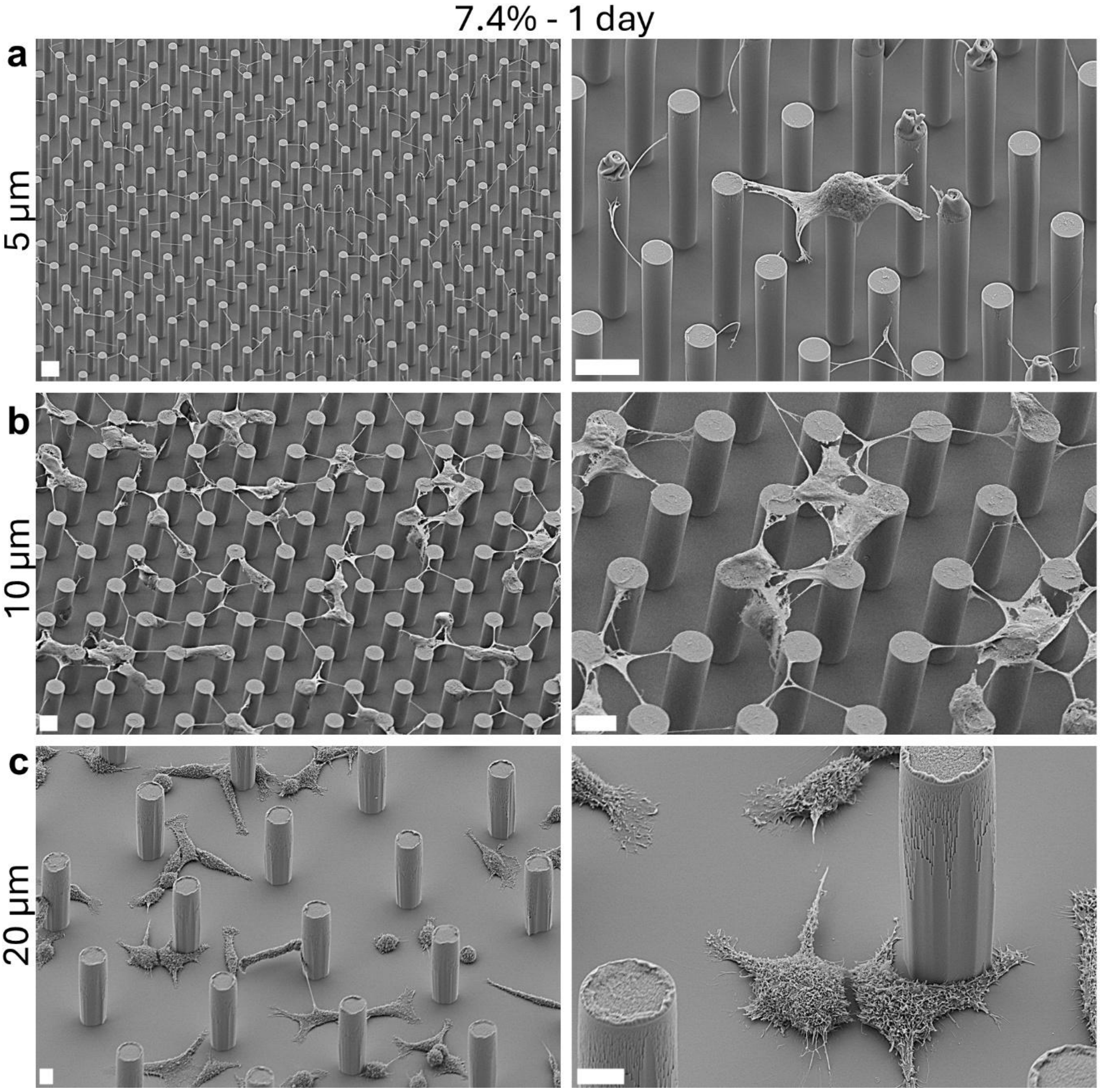

Figure S39. SEM images of superhydrophobic micropillared surfaces with 7.4% solid fraction after 1 day of cell incubation. **a–c**) Low (left) and high (right) magnification SEM images of 5 µm, 10 µm, and 20 µm pillar sizes, respectively. Scale bars: 10 µm.

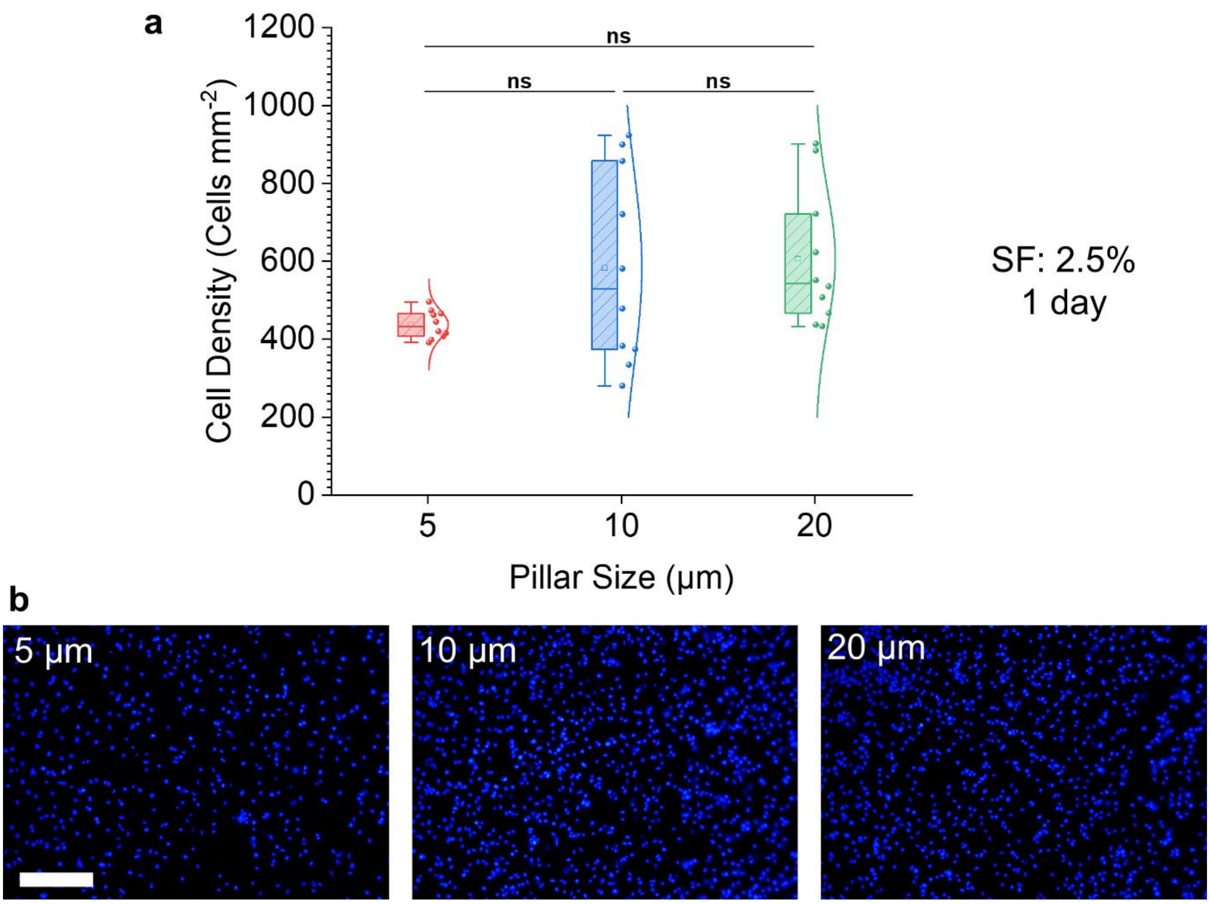

Figure S40. Influence of pillar size at 2.5% solid fraction on cell adhesion and morphology after 1 day of incubation. **a**) Cell density as a function of pillar size for surfaces. **b**) Low-magnification fluorescence microscopy images of cell adhesion on the micropillared surfaces. Scale bar: 200 µm.

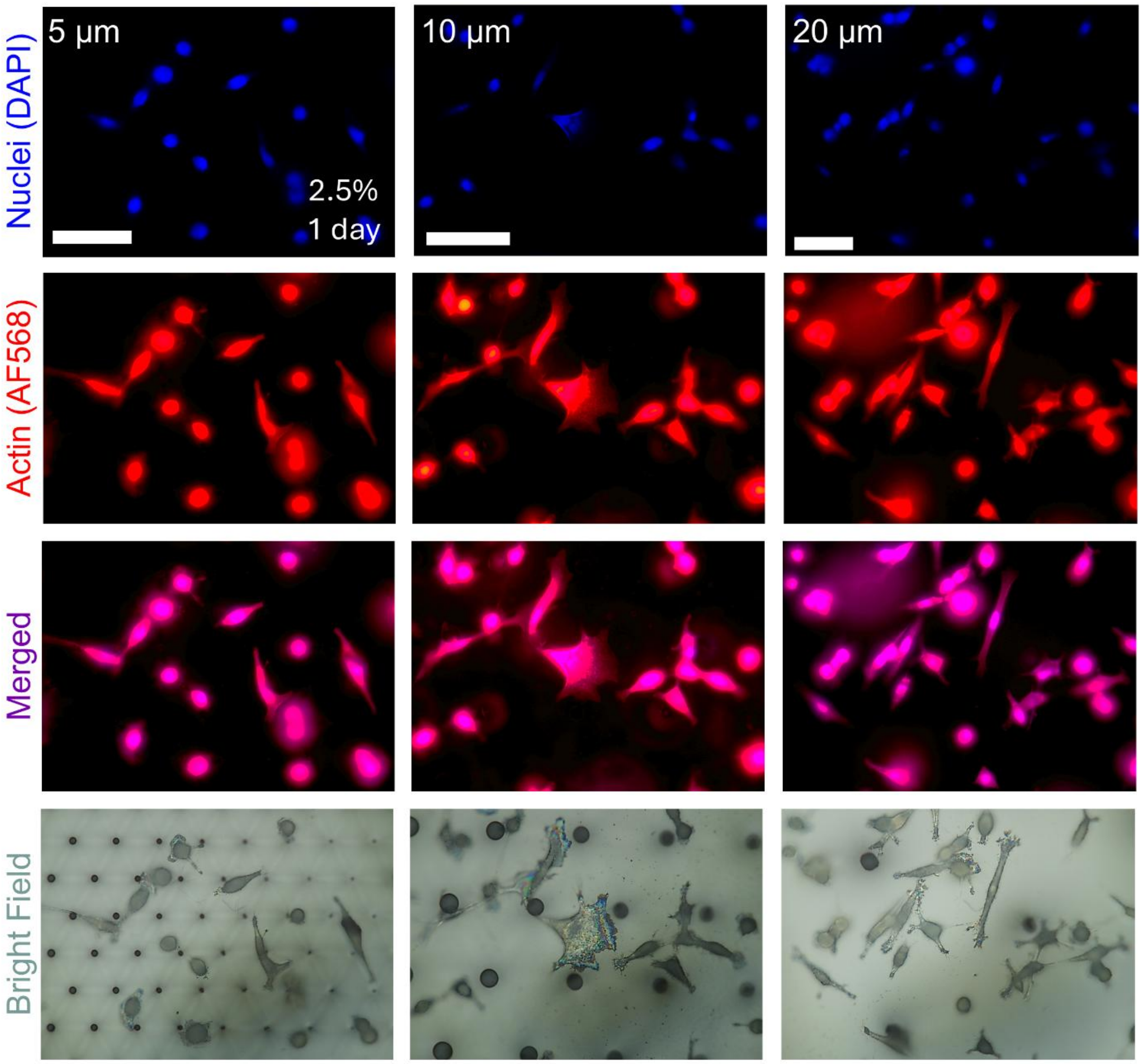

Figure S41. High-magnification fluorescence and bright field images displaying adhered cells on micropillar surfaces with varying pillar sizes (5, 10, and 20 µm) with 2.5% solid fraction after 1 day of incubation showing nuclei (DAPI, blue), actin cytoskeleton (AF568, red), and merged channels. Scale bars: 50 µm.

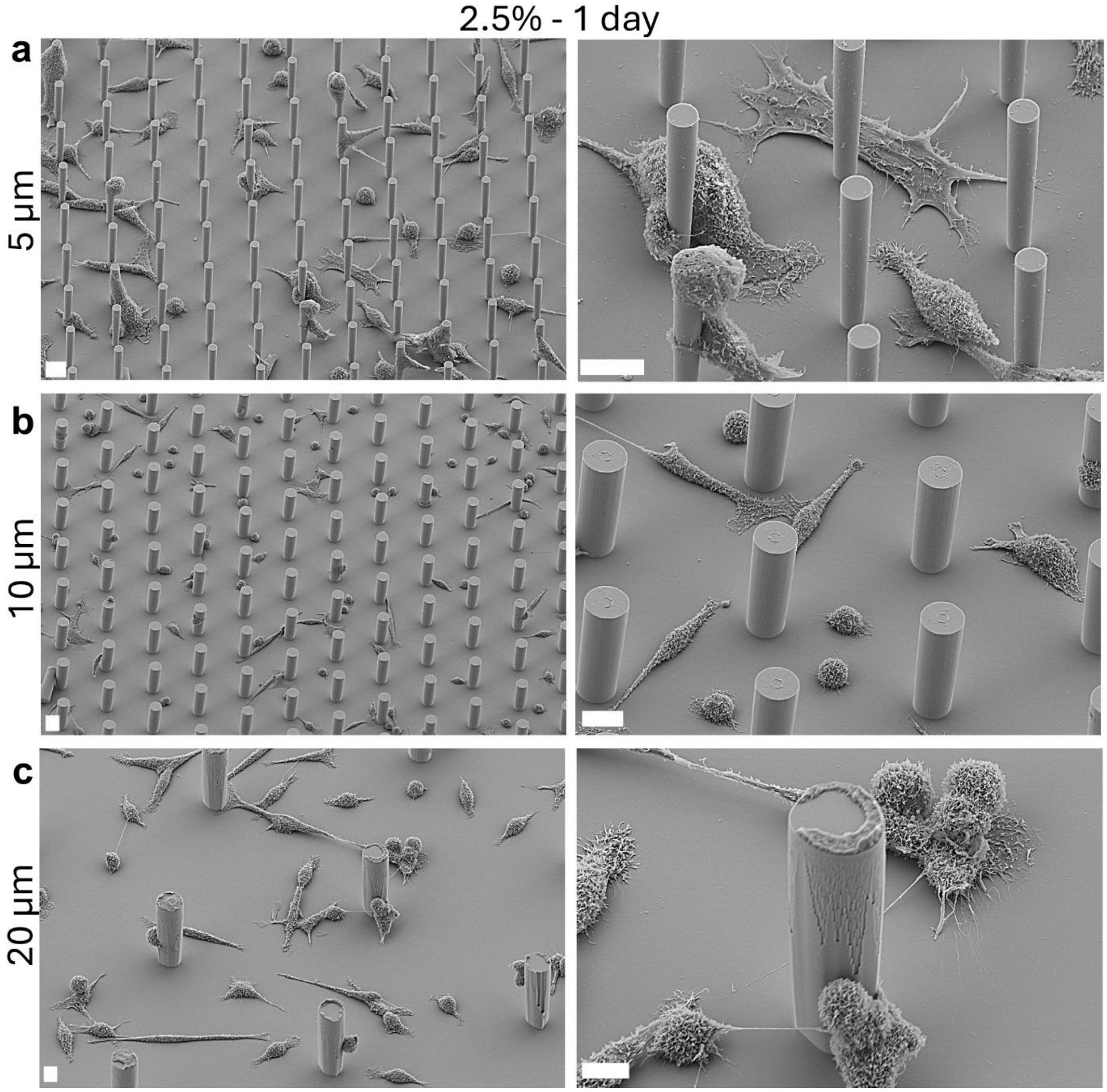

Figure S42. SEM images of superhydrophobic micropillared surfaces with 2.5% solid fraction after 1 day of cell incubation. **a–c**) Low (left) and high (right) magnification SEM images of 5 µm, 10 µm, and 20 µm pillar sizes, respectively. Scale bars: 10 µm.

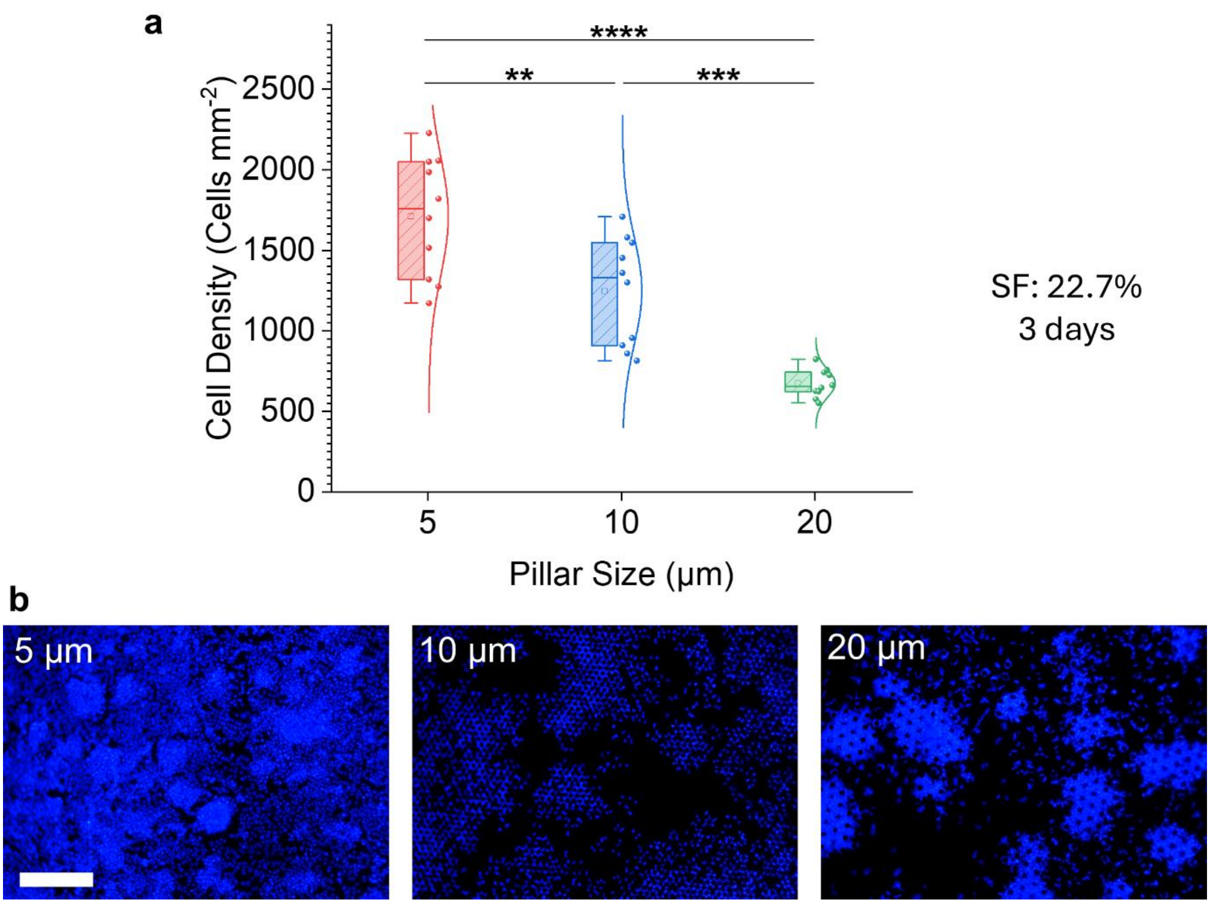

Figure S43. Influence of pillar size at 22.7% solid fraction on cell adhesion and morphology after 3 days of incubation. **a**) Cell density as a function of pillar size for surfaces. **b**) Low-magnification fluorescence microscopy images of cell adhesion on the micropillared surfaces. Scale bar: 200 μm.

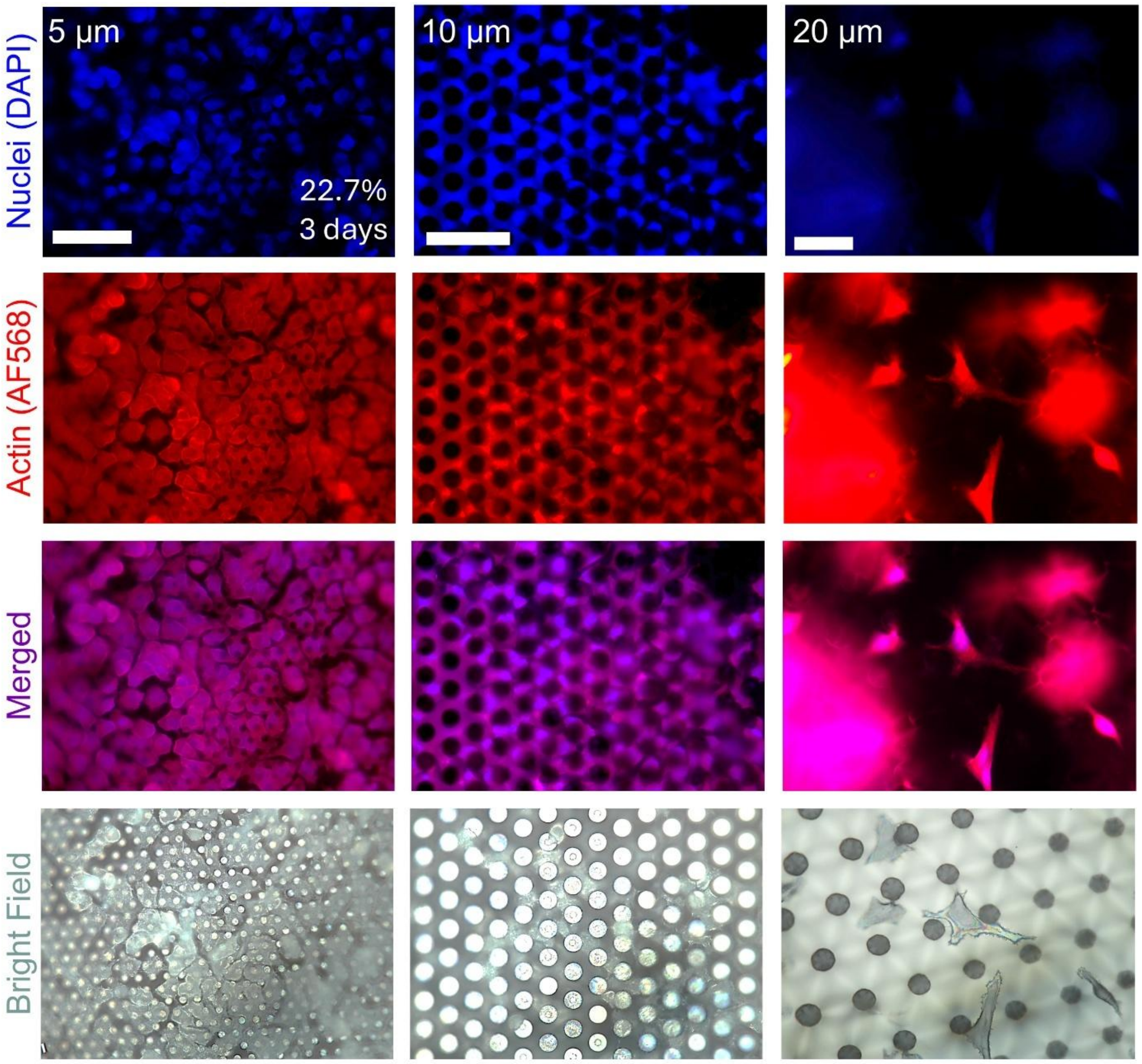

Figure S44. High-magnification fluorescence and bright field images displaying adhered cells on micropillar surfaces with varying pillar sizes (5, 10, and 20 μm) with 22.7% solid fraction after 3 days of incubation showing nuclei (DAPI, blue), actin cytoskeleton (AF568, red), and merged channels. Scale bars: 50 μm.

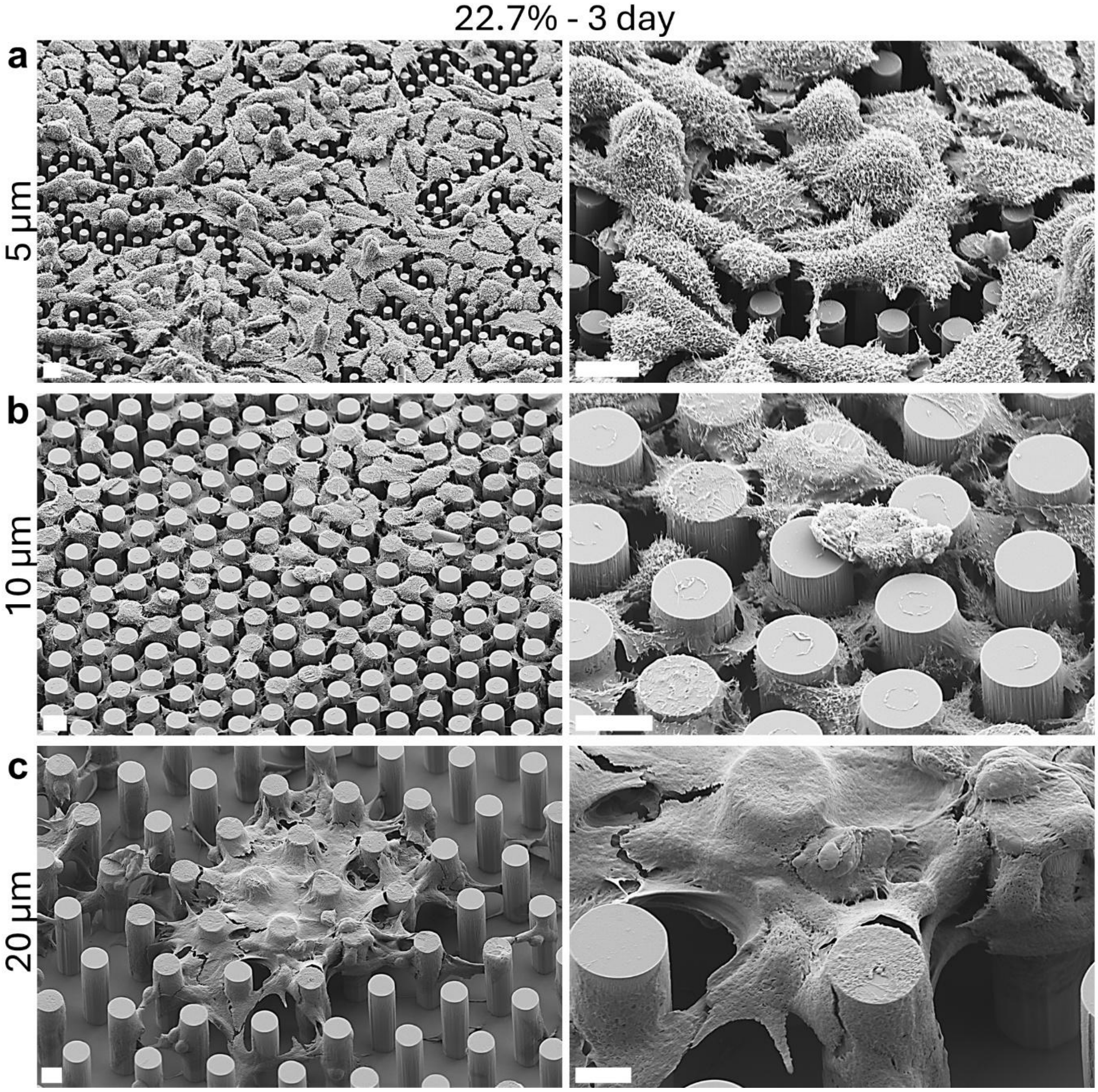

Figure S45. SEM images of hydrophobic micropillared surfaces with 22.7% solid fraction after 3 days of cell incubation. **a–c**) Low (left) and high (right) magnification SEM images of 5 µm, 10 µm, and 20 µm pillar sizes, respectively. Scale bars: 10 µm.

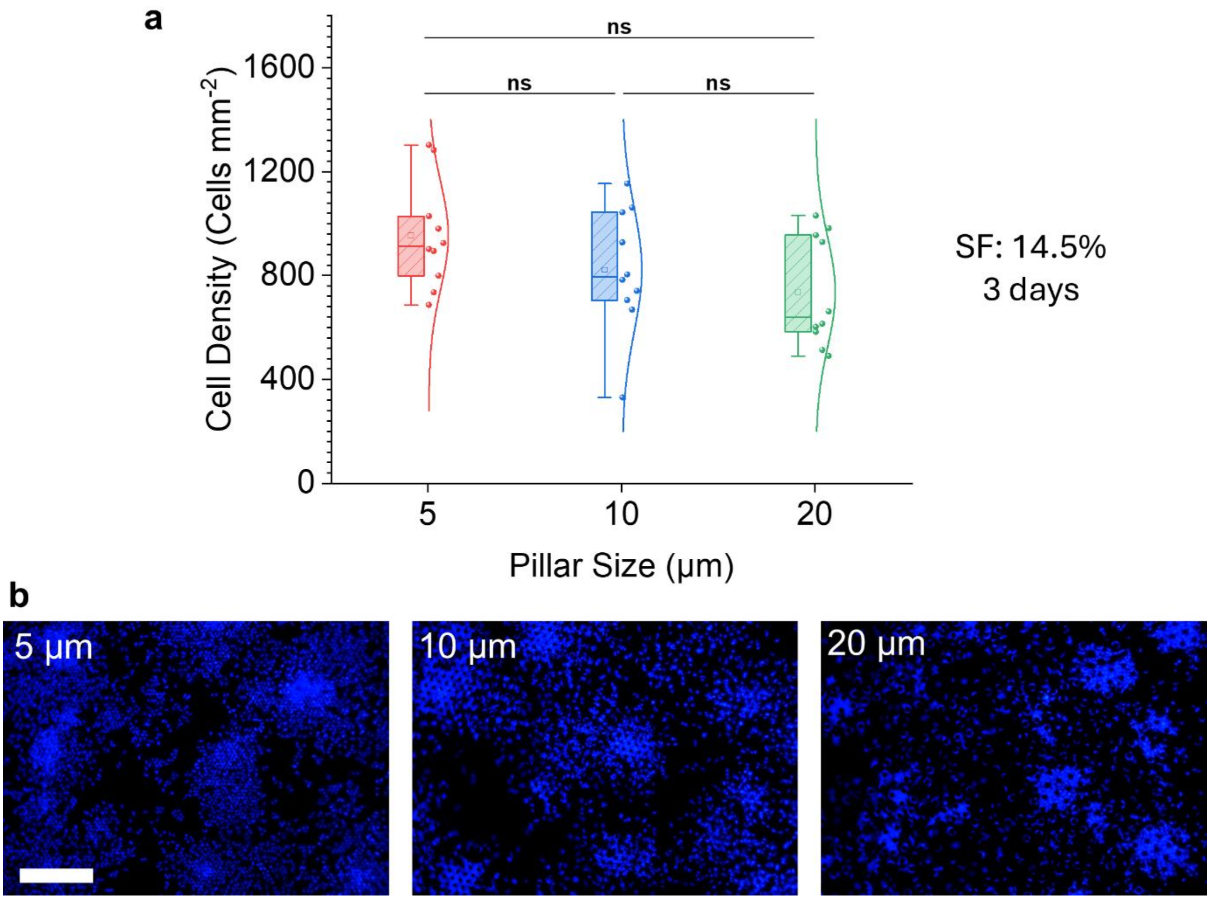

Figure S46. Influence of pillar size at 14.5% solid fraction on cell adhesion and morphology after 3 days of incubation. **a**) Cell density as a function of pillar size for surfaces. **b**) Low-magnification fluorescence microscopy images of cell adhesion on the micropillared surfaces. Scale bar: 200 µm.

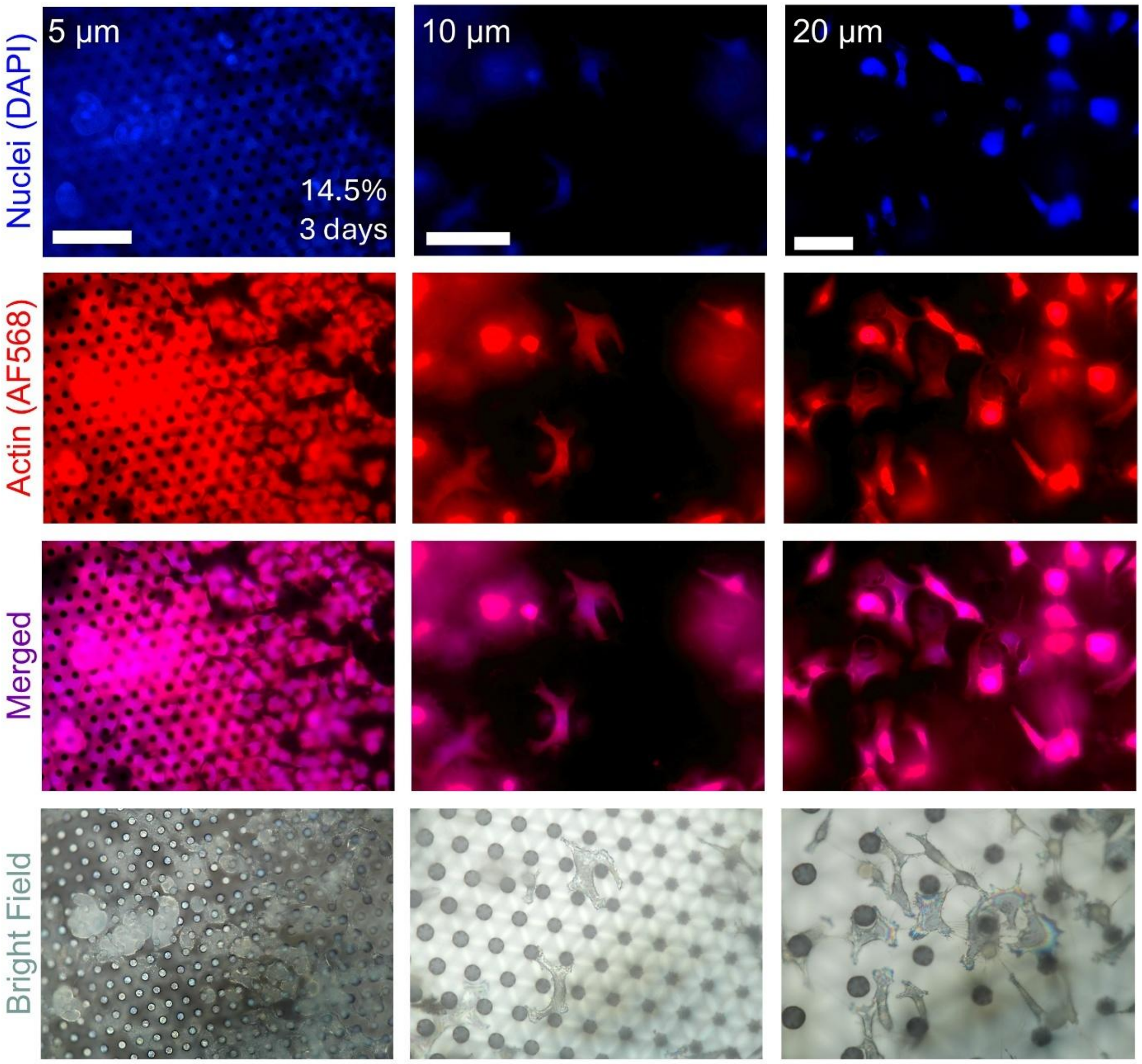

Figure S47. High-magnification fluorescence and bright field images displaying adhered cells on micropillar surfaces with varying pillar sizes (5, 10, and 20 µm) with 14.5% solid fraction after 3 days of incubation showing nuclei (DAPI, blue), actin cytoskeleton (AF568, red), and merged channels. Scale bars: 50 µm.

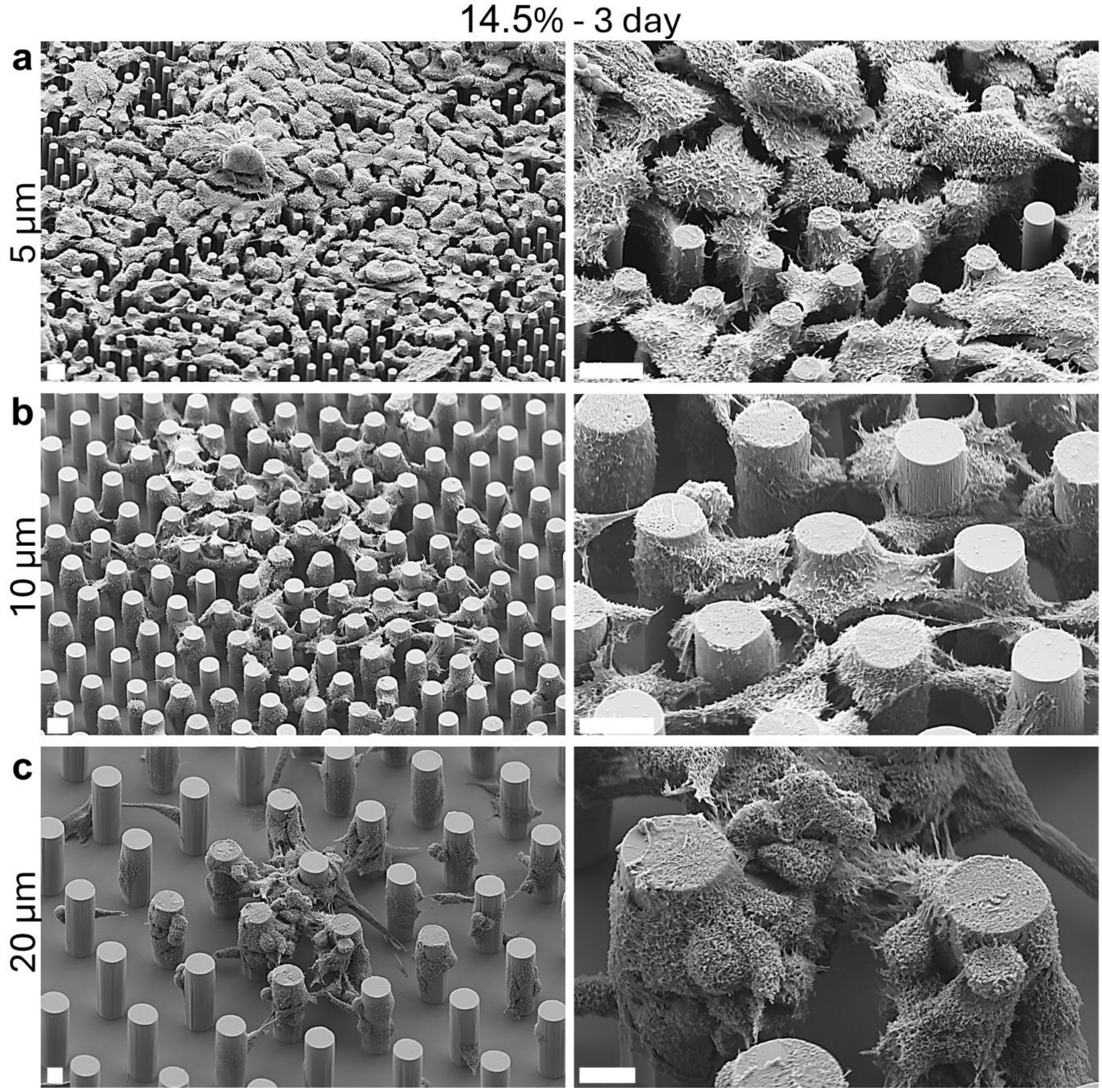

Figure S48. SEM images of hydrophobic micropillared surfaces with 14.5% solid fraction after 3 days of cell incubation. **a–c**) Low (left) and high (right) magnification SEM images of 5 µm, 10 µm, and 20 µm pillar sizes, respectively. Scale bars: 10 µm.

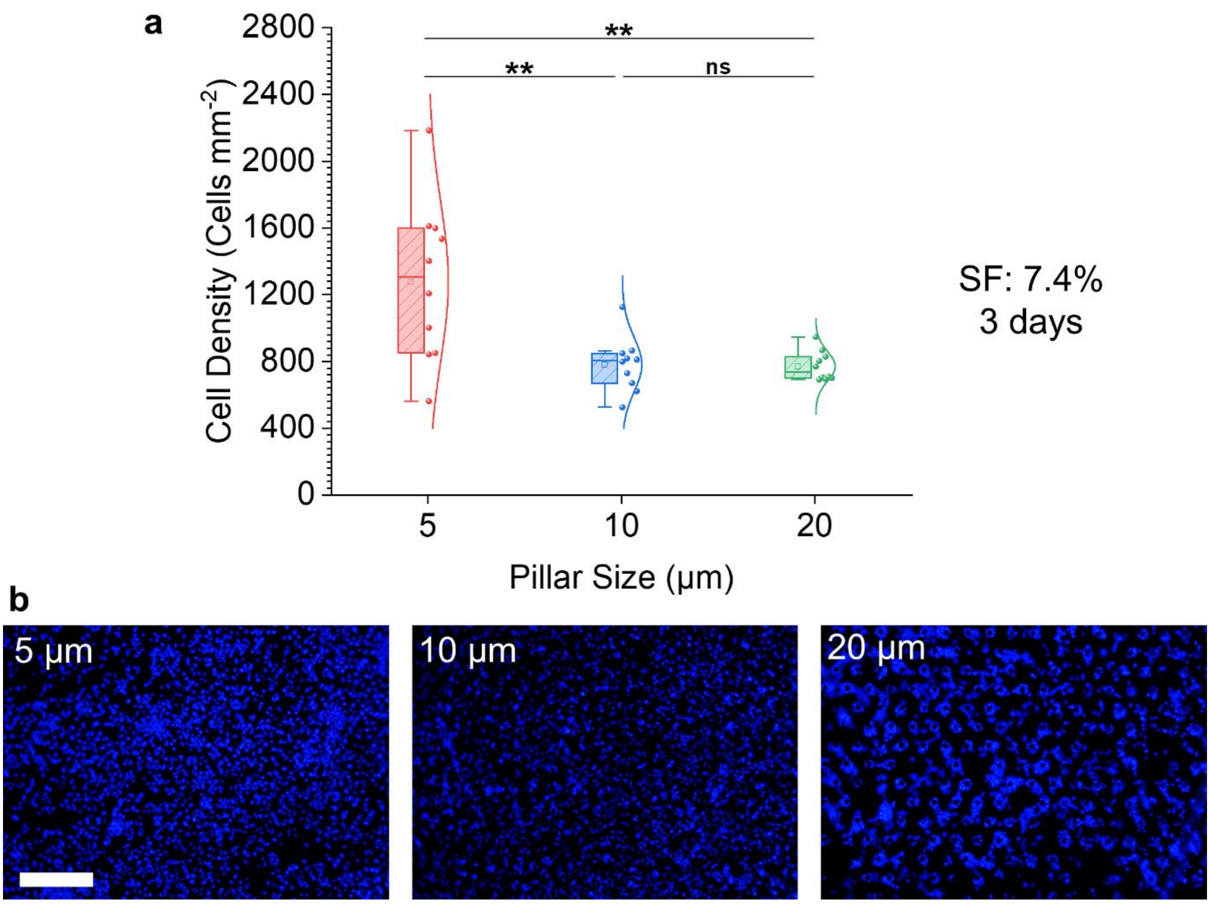

Figure S49. Influence of pillar size at 7.4% solid fraction on cell adhesion and morphology after 3 days of incubation. **a**) Cell density as a function of pillar size for surfaces. **b**) Low-magnification fluorescence microscopy images of cell adhesion on the micropillared surfaces. Scale bar: 200 µm.

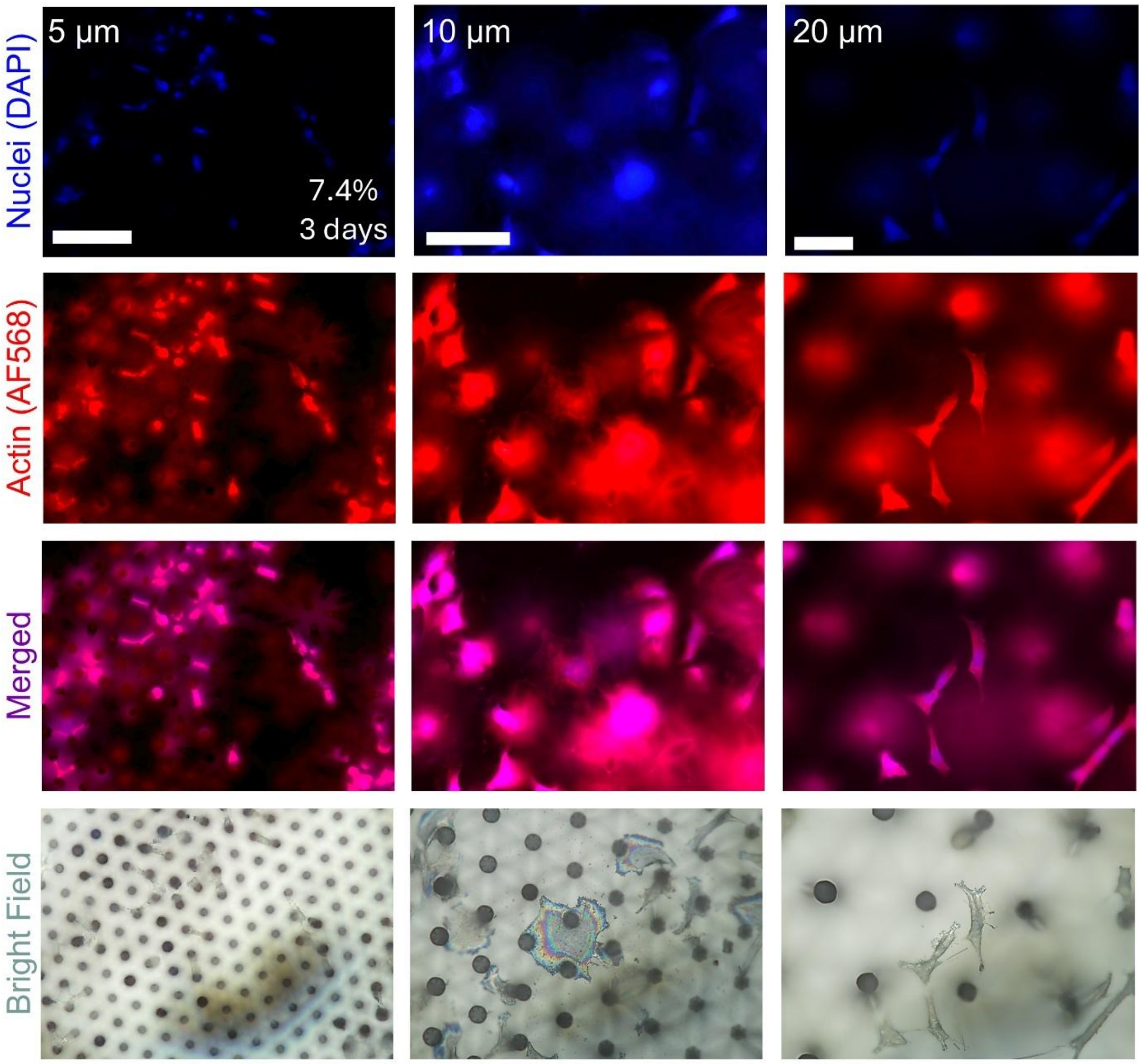

Figure S50. High-magnification fluorescence and bright field images displaying adhered cells on micropillar surfaces with varying pillar sizes (5, 10, and 20 μm) with 7.4% solid fraction after 3 days of incubation showing nuclei (DAPI, blue), actin cytoskeleton (AF568, red), and merged channels. Scale bars: 50 μm.

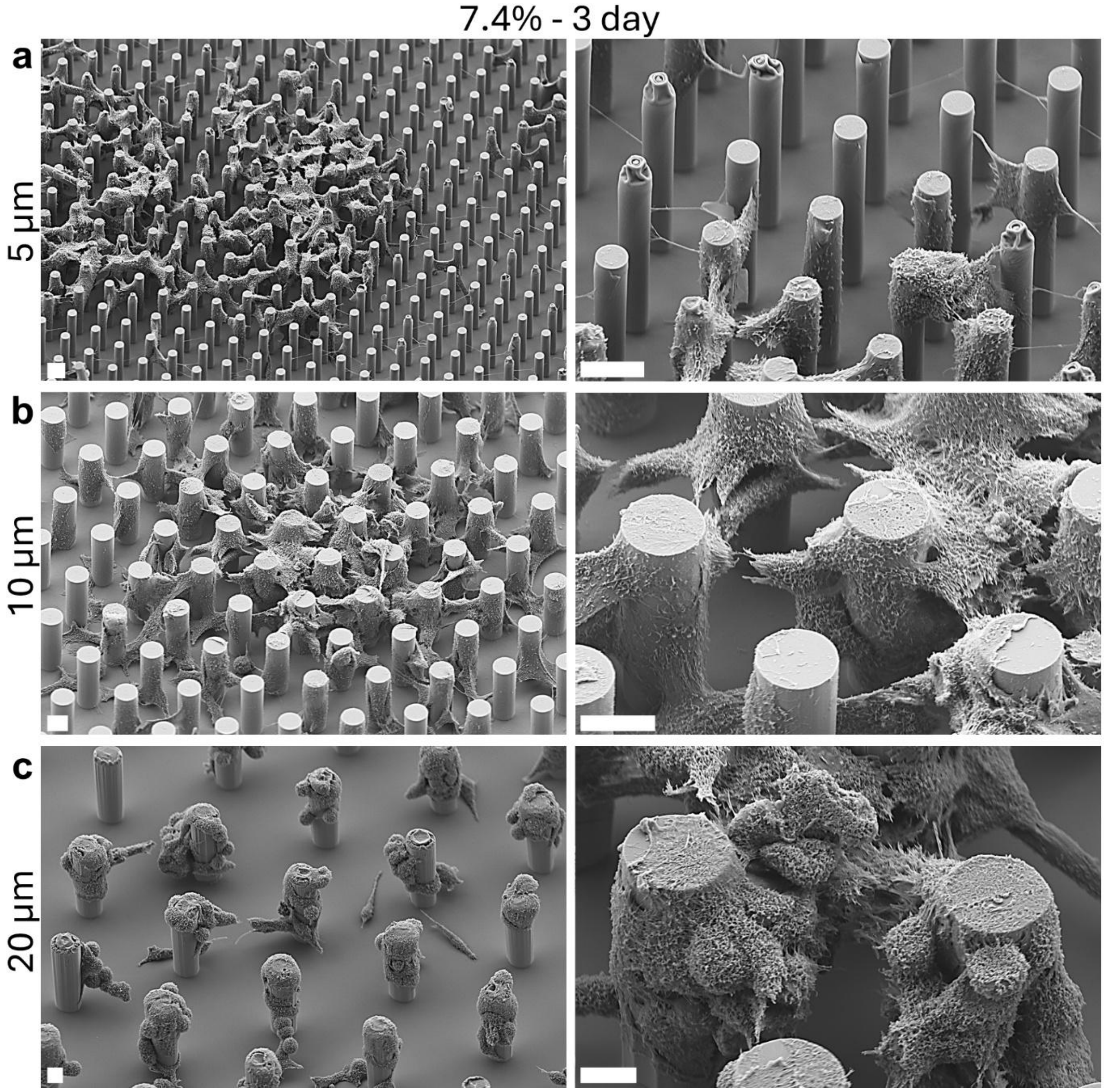

Figure S51. SEM images of superhydrophobic micropillared surfaces with 7.4% solid fraction after 3 days of cell incubation. **a–c**) Low (left) and high (right) magnification SEM images of 5 µm, 10 µm, and 20 µm pillar sizes, respectively. Scale bars: 10 µm.

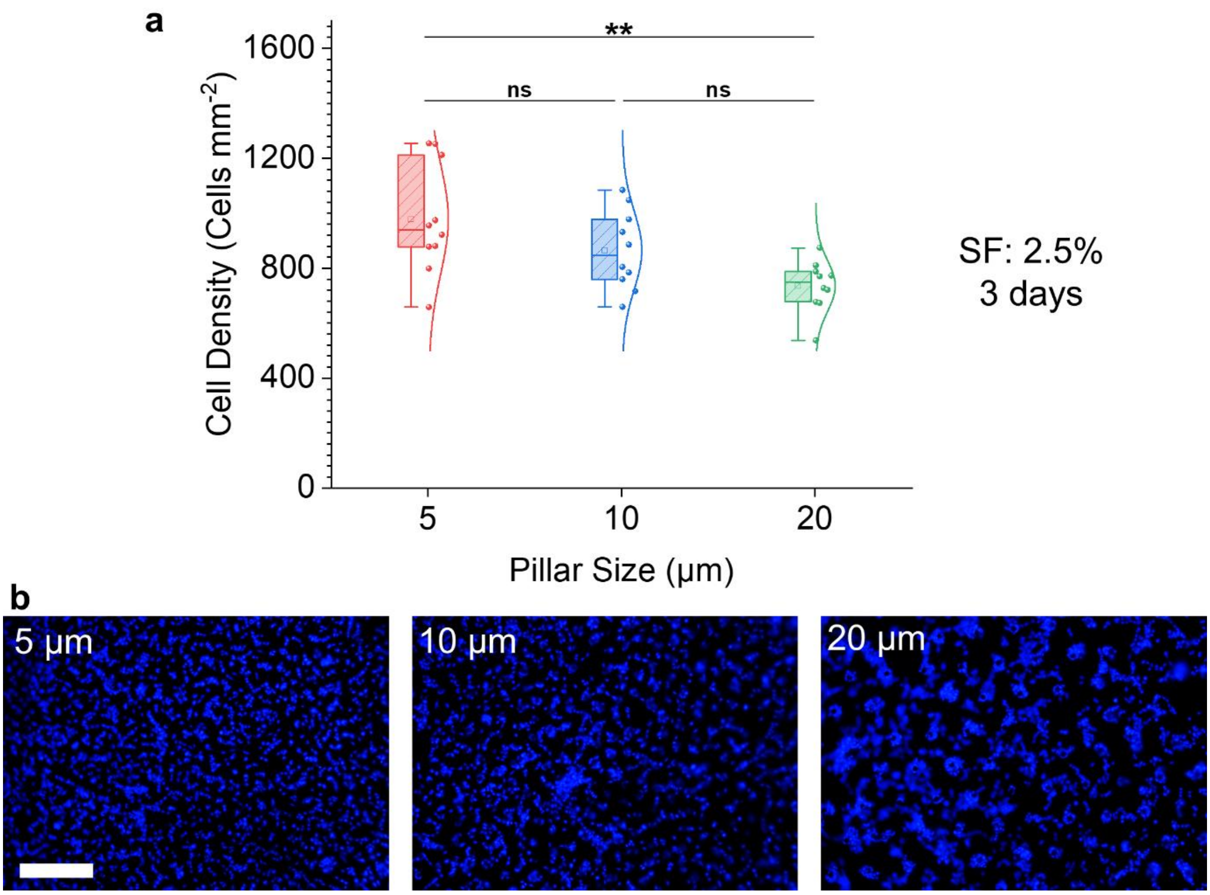

Figure S52. Influence of pillar size at 2.5% solid fraction on cell adhesion and morphology after 3 days of incubation. **a**) Cell density as a function of pillar size for surfaces. **b**) Low-magnification fluorescence microscopy images of cell adhesion on the micropillared surfaces. Scale bar: 200 µm.

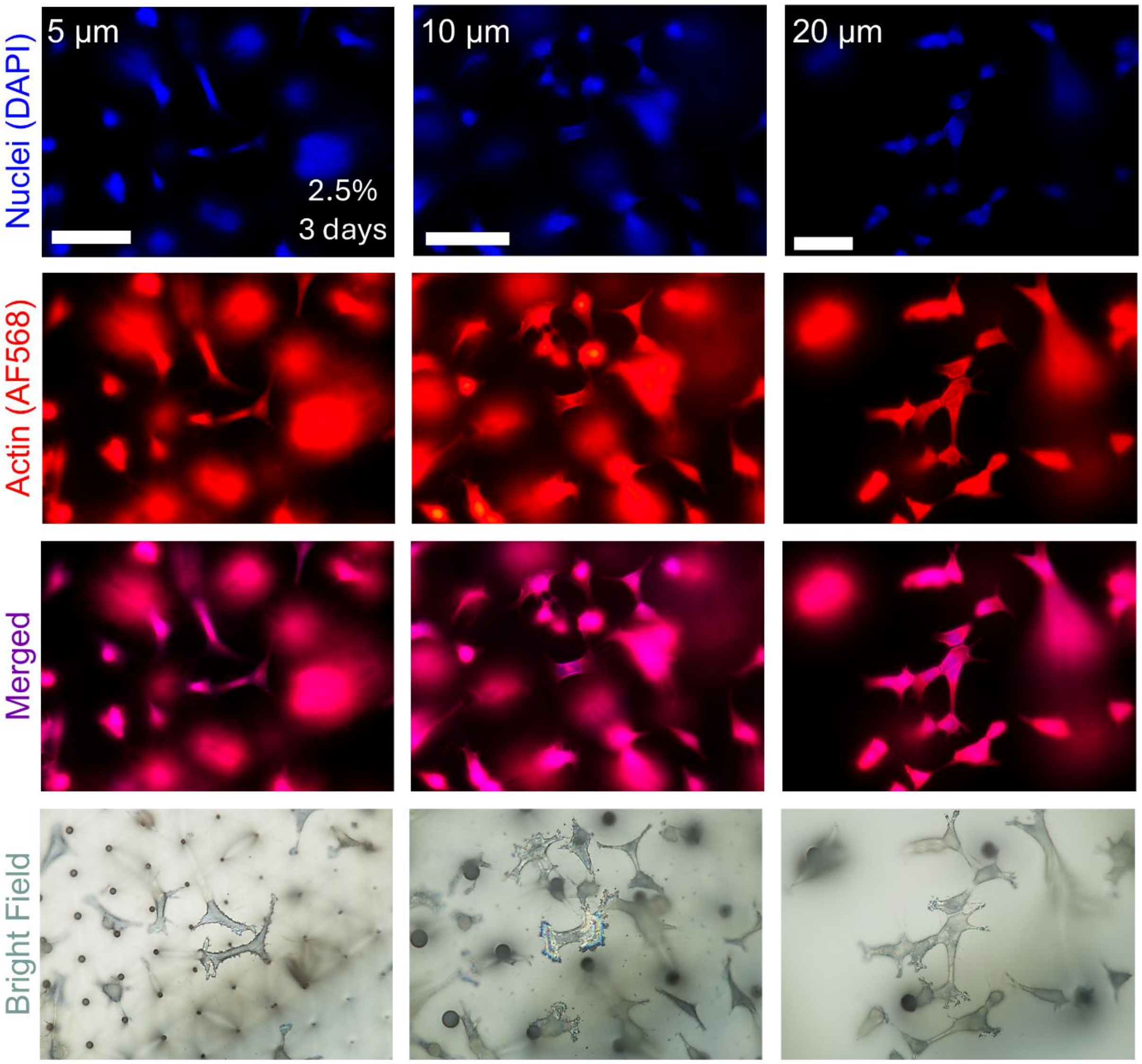

Figure S53. High-magnification fluorescence and bright field images displaying adhered cells on micropillar surfaces with varying pillar sizes (5, 10, and 20 µm) with 2.5% solid fraction after 3 days of incubation showing nuclei (DAPI, blue), actin cytoskeleton (AF568, red), and merged channels. Scale bars: 50 µm.

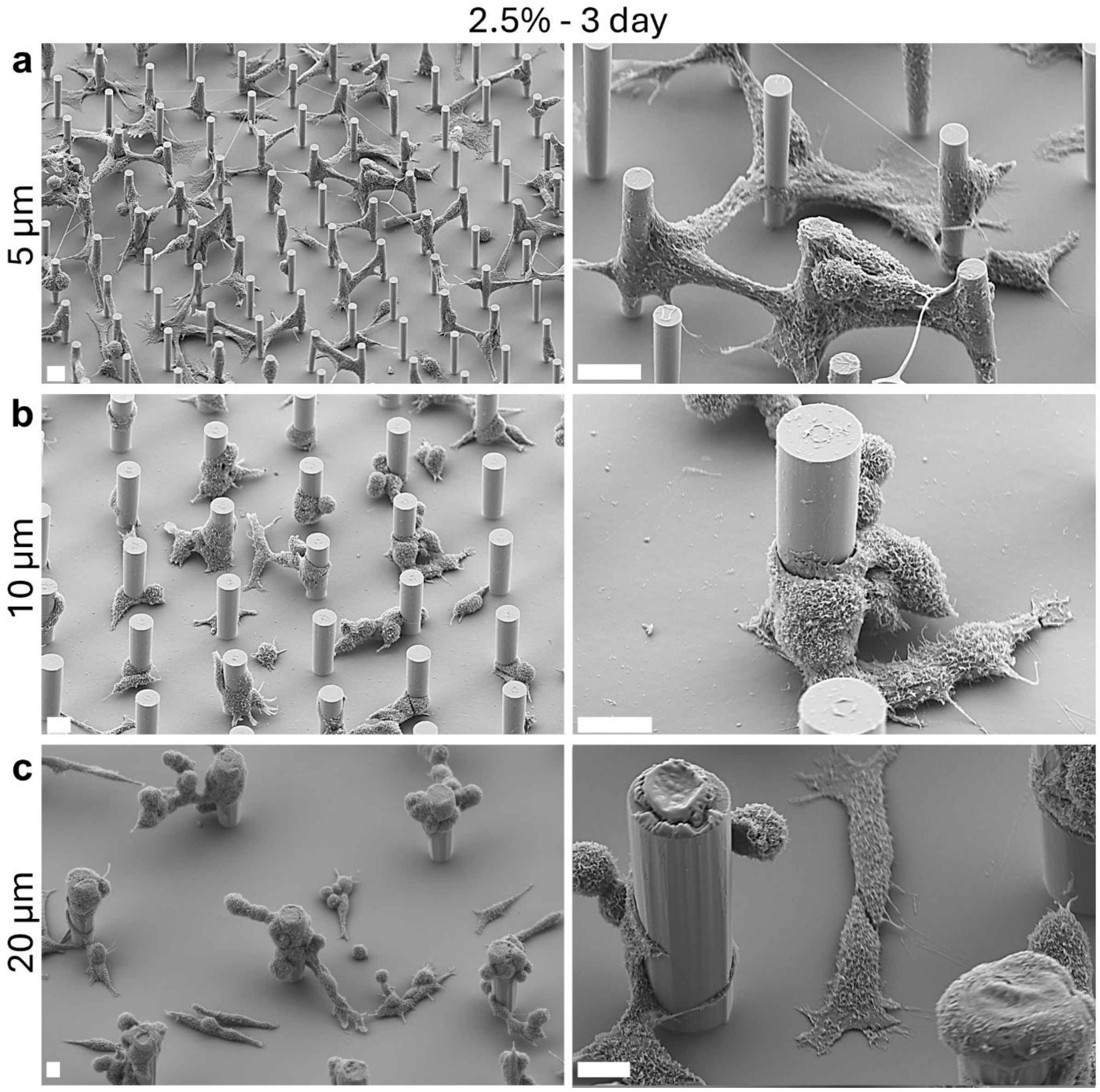

Figure S54. SEM images of superhydrophobic micropillared surfaces with 2.5% solid fraction after 3 days of cell incubation. **a–c**) Low (left) and high (right) magnification SEM images of 5 µm, 10 µm, and 20 µm pillar sizes, respectively. Scale bars: 10 µm.

S6. Effect of Pillar Height on Plastron Stability

The influence of pillar height on plastron longevity was previously studied by our group using micropillared Si surfaces with fixed lateral geometry and varying heights (15 μm and 40 μm).[9] The results revealed a complex interplay between pillar height and solid fraction. For intermediate solid fractions (7.4% and 14.5%), increasing pillar height significantly enhanced plastron stability by preventing meniscus penetration and maintaining the Cassie–Baxter state under immersion. For the lowest solid fraction (2.5%), both heights (15 and 40 μm) were smaller than the pillar spacing (50 μm), and thus, neither condition was sufficient to sustain a stable air layer. In contrast, for the highest solid fraction (22.7%), the increased air volume in the 40 μm tall pillars extended plastron lifetime nearly threefold compared to the shorter version (30 h vs. 10 h), underscoring that air volume and meniscus geometry both critically determine the air-retaining ability. That is why a 40 μm pillar height was selected for the present study to ensure robust and prolonged plastron retention in most surfaces. These findings, supported by wetting dynamics and collapse imaging, affirm that pillar height is a key design parameter for sustaining stable underwater superhydrophobicity in biofluid environments.

S7. Why Reported Cell Adhesion Results on Superhydrophobic Surfaces Vary

While most studies agree that superhydrophobic surfaces hinder eukaryotic cell adhesion, variations persist in the reported outcomes. Several interrelated factors can account for these discrepancies:

Wetting state (Cassie–Baxter vs. Wenzel): A critical determinant is whether the surface remains in the Cassie–Baxter state, maintaining an air plastron, or transitions to the Wenzel state, allowing direct wetting. Many superhydrophobic surfaces described as cell-adhesive may have been partially wetted due to poor plastron stability. Surfaces with high contact angle hysteresis (petal effect) can retain water and support adhesion, while lotus-like surfaces remain cell-repellent. Inconsistent wetting due to preparation factors such as surface contamination or surfactants can also explain divergent results.[9,29–32]

Feature geometry and scale: The micro/nano architecture itself can cause different cell responses. Even among surfaces that are "superhydrophobic" (by static contact angle), a texture composed of sparse micropillars is not the same as a dense nanoneedle forest. Cell types might span or settle differently. For example, a fibroblast might be able to attach across a 20 µm micropillar gap if the gap eventually wets, but it may not find purchase on a surface covered in 100 nm spikes. Smaller features generally correlate with less cell adhesion, but an extremely high aspect ratio microtexture might physically trap a cell (e.g. if a cell falls into a deep microwell in the absence of a plastron). It has indeed been noted that the spacing and arrangement of features can result in cell-selective adhesion patterns. Variations in roughness hierarchy (uni-modal vs. dual-modal roughness) also matter – hierarchical surfaces tend to maintain Cassie–Baxter state better and thus more consistently repel cells. In contrast, single-scale roughness (especially at the microscale) may allow partial wetting and patchy cell adhesion. These geometrical differences can explain why, say, one superhydrophobic polymer

film allowed a few cells to attach (perhaps larger pores, partial Wenzel) while another with finer texture allowed none.[6,30,31]

Surface chemistry and energy: Not all superhydrophobic surfaces are chemically alike. Some use fluorinated coatings, others hydrophobic hydrocarbons or silicones. Beyond simply being water-repellent, these chemistries can interact with proteins differently. Hydrophobic surfaces tend to denature proteins upon adsorption (exposing hydrophobic residues), which can either promote or inhibit subsequent cell binding depending on the protein. In Zhao's nanowire study,[13] two very different chemistries (carbon vs. TiO_2) both yielded cell-repellency, suggesting geometry trumped chemistry. But in other cases, chemistry plays a role – e.g. a mildly hydrophobic surface might allow a protein monolayer that supports some cell attachment, whereas a superhydrophobic perfluorinated surface might delay protein spreading. Additionally, surface chemistry affects plastron stability. Thus, inconsistent chemical stability or differing surface energy (e.g. a contact angle of 150° vs. 170°) can lead to different adhesion outcomes.[13,14]

Protein presence and media conditions: The makeup of the cell culture medium (e.g. serum protein concentration) and flow conditions can influence results. High protein environments can accelerate the collapse of the Cassie–Baxter state, thus shortening the cell-repellency duration. If one experiment was done in serum-free media (or for short duration before protein adsorption), cells might never find footholds, whereas in a serum-rich, long-term culture, proteins could eventually wet the surface and allow some attachment. Flow or agitation can also remove weakly adhered proteins/cells – some anti-adhesive effects of superhydrophobic surfaces manifest more under flow (the "self-cleaning" removes cells that would otherwise loosely stick).

Differences in incubation time are critical: many studies only look at 4–24 h attachment, but a few that extended to days saw eventual changes as proteins accumulated. Thus, comparing studies, one should note the timeframe and media; a surface might be cell-resistant initially but not after several days.[21,30]

Cell type and state: Although most adherent cells respond similarly (reduced adhesion) to superhydrophobic surfaces, there can be subtle differences. Certain cell types with smaller size or less reliance on focal adhesions (e.g. some immune cells or highly transformed cancer cells) might show slightly higher tolerance for rough, hydrophobic substrates. Conversely, cells that require a specific matrix protein to attach (e.g. osteoclast precursors needing vitronectin) might fail completely if that protein does not adsorb. The literature primarily shows a consistent trend across fibroblasts, osteoblasts, endothelial, etc., but variance in cell line robustness or integrin expression could tweak outcomes. For example, MG63 osteosarcoma cells showed 90% lower adhesion on a superhydrophobic surface than on a hydrophilic one, yet their proliferation was poor on both.[18] Another cell line might proliferate better on a moderately hydrophobic surface. Therefore, results can differ if one study uses a notably different cell model or if cells were in a different physiological state (e.g. suspended vs. trypsinized vs. confluent seeding).[18,30]

Assessment methods: Lastly, the definition of "adhesion" or "repellency" can vary. Some papers consider a surface cell-repellent if very few cells remain attached after rinsing (population-level study), others look at cell morphology (round vs. spread – single cell study) or longer-term proliferation. A surface might allow initial cell attachment (so one could report some adhesion), but if those cells do not survive or divide, another perspective is that the surface effectively resists sustained colonization. Differences in assay (staining for attached cell number, measuring viability, microscopy of morphology) could lead to different interpretations. Additionally, any surface toxicity should be ruled out – truly superhydrophobic

surfaces are often made of biocompatible materials (PTFE, silica, etc.), but if a coating leaches something, reduced cell numbers might be partially due to cytotoxicity rather than just lack of adhesion.[21,30]

S8. Epithelial vs. Fibroblasts as a Cell Model – Rationale for Selecting A549 Cells

1. Epithelial cells, such as A549, primarily adhere through tight junctions, adherens junctions, and desmosomes, forming continuous monolayers that closely mimic in vivo epithelial barriers. In contrast, fibroblasts rely more on focal adhesions and stress fibres to migrate and remodel their extracellular environment. Because fibroblasts exhibit stronger adhesion and contractility, they may not be as sensitive to surface wetting transitions as epithelial cells, potentially leading to less distinct differences in adhesion behavior across superhydrophobic and hydrophilic surfaces.
2. A549 cells, as adherent epithelial cells, depend on integrin-mediated adhesion and protein adsorption for surface attachment. Their adhesion behavior is highly affected by surface wettability and the presence of an air plastron, making them an ideal model to study how superhydrophobic micro and nanostructures influence cell repulsion. In contrast, fibroblasts, due to their mesenchymal nature, can exhibit stronger adhesion forces, enabling them to partially spread even on anti-adhesive surfaces, thereby masking subtle effects.
3. A549 cells are widely used as a model for respiratory epithelial barriers, making them particularly relevant for evaluating the potential of superhydrophobic surfaces in biomedical applications, such as biofouling-resistant coatings for medical implants and airway interfaces. Since epithelial cells are the first line of contact in many biomedical settings, understanding their interactions with engineered surfaces is critical. Fibroblasts, while important for wound healing and tissue remodelling, may not directly represent the primary cell type encountered in applications involving surface-based cell Repellency.
4. Previous studies have demonstrated that epithelial cells are more sensitive to plastron stability due to their preference for continuous substrate contact. The presence or absence of an air layer significantly affects their ability to establish adhesion and proliferate.

Fibroblasts, due to their ability to bridge across larger gaps and remodel the ECM, may overcome initial repellent effects, leading to a less pronounced distinction between surface types.[33–36]

5. The mechanism of cell Repellency in this study is based on the entrapment of the air plastron (Cassie–Baxter state), which *physically* prevents direct contact between cells and the underlying substrate. This is, likely, a cell-type independent mechanism, governed primarily by the physical barrier and reduced effective contact area, rather than specific biochemical cues. Therefore, while A549 epithelial cells were chosen as a model system due to their relevance and sensitivity, we expect that other adherent mammalian cells (e.g., fibroblasts, endothelial cells) will exhibit qualitatively similar repellent behavior on stable superhydrophobic plastron-retaining surfaces, especially in early time points where plastron integrity is maintained.

S9. Experimental Section

Table S1. The design parameters of the studied the micropillars.

Pillar Diameter (μm)	Pitch (μm)	Solid Fraction (%)	Wenzel Roughness
2	4	22.7	10.1
	5	14.5	6.8
	7	7.4	4.0
	12	2.5	2.0
5	10	22.7	8.3
	12.5	14.5	5.6
	17.5	7.4	3.4
	30	2.5	1.8
10	20	22.7	4.6
	25	14.5	3.3
	35	7.4	2.2
	60	2.5	1.4
20	40	22.7	3.3
	50	14.5	2.5
	70	7.4	1.7
	120	2.5	1.3
30	60	22.7	2.5
40	80	22.7	2.1
50	100	22.7	1.9

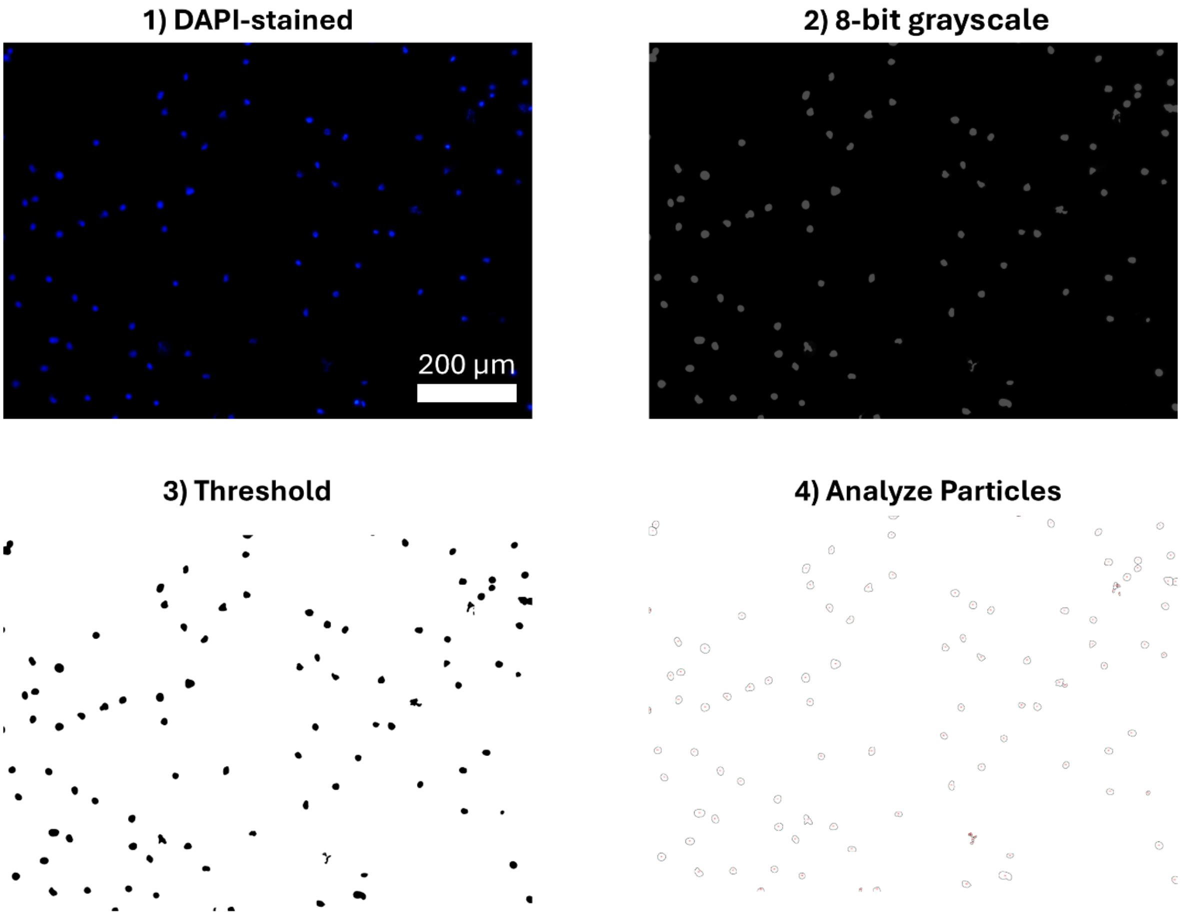

Figure S55. Stepwise processing of DAPI–stained fluorescence images for cell counting using ImageJ. (1) Original fluorescence image of DAPI–stained nuclei (blue). scale bar: 200 µm. (2) Conversion to 8-bit grayscale to enhance contrast for segmentation. (3) Thresholding applied to isolate nuclei from the background. (4) Particle analysis performed to count individual nuclei, with outlines highlighting detected cells. This automated image processing pipeline ensures accurate and reproducible quantification of adhered A549 cells.

Supporting References

(1) Wu, X.; Li, L.; Wang, L.; Lei, Z.; Yang, F.; Liu, R.; Wang, Y.; Peng, K.; Wang, Z. Cell Spreading Behaviors on Hybrid Nanopillar and Nanohole Arrays. *Nanotechnology* **2022**, *33* (4). https://doi.org/10.1088/1361-6528/ac084a.

(2) Flamourakis, G.; Dong, Q.; Kromm, D.; Teurlings, S.; Haren, J. van; Allertz, T.; Smeenk, H.; Vrij, F. M. S. de; Tas, R. P.; Smith, C. S.; Brinks, D.; Accardo, A. Deciphering the Influence of Effective Shear Modulus on Neuronal Network Directionality and Growth Cones' Morphology via Laser-Assisted 3D-Printed Nanostructured Arrays. *Adv Funct Mater* **2025**, *35* (5), 2409451. https://doi.org/10.1002/adfm.202409451.

(3) Nguyen, A. T.; Sathe, S. R.; Yim, E. K. F. From Nano to Micro: Topographical Scale and Its Impact on Cell Adhesion, Morphology and Contact Guidance. *Journal of Physics Condensed Matter*. Institute of Physics Publishing April 12, 2016. https://doi.org/10.1088/0953-8984/28/18/183001.

(4) Bourkoula, A.; Mavrogonatou, E.; Pavli, P.; Petrou, P. S.; Douvas, A. M.; Argitis, P.; Kletsas, D.; Kakabakos, S. E. Guided Cell Adhesion, Orientation, Morphology and Differentiation on Silicon Substrates Photolithographically Micropatterned with a Cell-Repellent Cross-Linked Poly(Vinyl Alcohol) Film. *Biomedical Materials (Bristol)* **2019**, *14* (1). https://doi.org/10.1088/1748-605X/aae7ba.

(5) Vu, H. H.; Nguyen, N. T.; Yadav, S.; Nguyen, T. T. H.; Kashaninejad, N. Roles of Micropillar Topography and Surface Energy on Cancer Cell Dynamics. *Technologies 2024, Vol. 12, Page 130* **2024**, *12* (8), 130. https://doi.org/10.3390/TECHNOLOGIES12080130.

(6) Hejazi, I.; Seyfi, J.; Hejazi, E.; Sadeghi, G. M. M.; Jafari, S. H.; Khonakdar, H. A. Investigating the Role of Surface Micro/Nano Structure in Cell Adhesion Behavior of Superhydrophobic Polypropylene/Nanosilica Surfaces. *Colloids Surf B Biointerfaces* **2015**, *127*, 233–240. https://doi.org/10.1016/j.colsurfb.2015.01.054.

(7) Ranella, A.; Barberoglou, M.; Bakogianni, S.; Fotakis, C.; Stratakis, E. Tuning Cell Adhesion by Controlling the Roughness and Wettability of 3D Micro/Nano Silicon Structures. *Acta Biomater* **2010**, *6* (7), 2711–2720. https://doi.org/10.1016/j.actbio.2010.01.016.

(8) Wang, Y.; Sims, C. E.; Marc, P.; Bachman, M.; Li, G. P.; Allbritton, N. L. Micropatterning of Living Cells on a Heterogeneously Wetted Surface. *Langmuir* **2006**, *22* (19), 8257–8262. https://doi.org/10.1021/LA061602K.

(9) Awashra, M.; Mirmohammadi, S. M.; Meng, L.; Franssila, S.; Jokinen, V. Stable Air Plastron Prolongs Biofluid Repellency of Submerged Superhydrophobic Surfaces. *Langmuir* **2025**, *41* (3), 1807. https://doi.org/https://doi.org/10.1021/acs.langmuir.4c04259.

(10) Zhang, J.; Li, G.; Man, J.; Qu, Y.; Guo, Z.; Zhang, S.; Li, D. Mechanism of Anti-Proteins Adsorption Behavior on Superhydrophobic Titanium Surface. *Surf Coat Technol* **2021**, *421*. https://doi.org/10.1016/j.surfcoat.2021.127421.

(11) Koc, Y.; De Mello, A. J.; McHale, G.; Newton, M. I.; Roach, P.; Shirtcliffe, N. J. Nano-Scale Superhydrophobicity: Suppression of Protein Adsorption and Promotion of Flow-Induced Detachment. *Lab Chip* **2008**, *8* (4), 582–586. https://doi.org/10.1039/b716509a.

(12) Kurylowicz, M.; Paulin, H.; Mogyoros, J.; Giuliani, M.; Dutcher, J. R. The Effect of Nanoscale Surface Curvature on the Oligomerization of Surface-Bound Proteins. *J R Soc Interface* **2014**, *11* (94). https://doi.org/10.1098/RSIF.2013.0818.

(13) Zhao, L.; Hu, L.; Huo, K.; Zhang, Y.; Wu, Z.; Chu, P. K. Mechanism of Cell Repellence on Quasi-Aligned Nanowire Arrays on Ti Alloy. *Biomaterials* **2010**, *31* (32), 8341–8349. https://doi.org/10.1016/J.BIOMATERIALS.2010.07.036.

(14) Meng, J.; Yang, G.; Liu, L.; Song, Y.; Jiang, L.; Wang, S. Cell Adhesive Spectra along Surface Wettability Gradient from Superhydrophilicity to Superhydrophobicity. *Sci China Chem* **2017**, *60* (5), 614–620. https://doi.org/10.1007/s11426-016-9031-8.

(15) Marcon, L.; Addad, A.; Coffinier, Y.; Boukherroub, R. Cell Micropatterning on Superhydrophobic Diamond Nanowires. *Acta Biomater* **2013**, *9* (1), 4585–4591. https://doi.org/10.1016/j.actbio.2012.08.026.

(16) Kontziampasis, D.; Bourkoula, A.; Petrou, P.; Tserepi, A.; Kakabakos, S.; Gogolides, E. Cell Array Fabrication by Plasma Nanotexturing. *https://doi.org/10.1117/12.2017894* **2013**, *8765*, 67–76. https://doi.org/10.1117/12.2017894.

(17) Piret, G.; Galopin, E.; Coffinier, Y.; Boukherroub, R.; Legrand, D.; Slomianny, C. Culture of Mammalian Cells on Patterned Superhydrophilic/Superhydrophobic Silicon Nanowire Arrays. *Soft Matter* **2011**, *7* (18), 8642–8649. https://doi.org/10.1039/c1sm05838j.

(18) Dowling, D. P.; Miller, I. S.; Ardhaoui, M.; Gallagher, W. M. Effect of Surface Wettability and Topography on the Adhesion of Osteosarcoma Cells on Plasma-Modified Polystyrene. *J Biomater Appl* **2011**, *26* (3), 327–347. https://doi.org/10.1177/0885328210372148.

(19) Ballester-Beltrán, J.; Rico, P.; Moratal, D.; Song, W.; Mano, J. F.; Salmerón-Sánchez, M. Role of Superhydrophobicity in the Biological Activity of Fibronectin at the Cell-Material Interface. *Soft Matter* **2011**, *7* (22), 10803–10811. https://doi.org/10.1039/c1sm06102j.

(20) Roach, P.; Shirtcliffe, N. J.; Farrar, D.; Perry, C. C. Quantification of Surface-Bound Proteins by Fluorometric Assay: Comparison with Quartz Crystal Microbalance and Amido Black Assay. *Journal of Physical Chemistry B* **2006**, *110* (41), 20572–20579. https://doi.org/10.1021/jp0621575.

(21) Bauer, S.; Park, J.; Mark, K. von der; Schmuki, P. Improved Attachment of Mesenchymal Stem Cells on Super-Hydrophobic TiO2 Nanotubes. *Acta Biomater* **2008**, *4* (5), 1576–1582. https://doi.org/10.1016/J.ACTBIO.2008.04.004.

(22) Ko, T. J.; Kim, E.; Nagashima, S.; Oh, K. H.; Lee, K. R.; Kim, S.; Moon, M. W. Adhesion Behavior of Mouse Liver Cancer Cells on Nanostructured Superhydrophobic and Superhydrophilic Surfaces. *Soft Matter* **2013**, *9* (36), 8705–8711. https://doi.org/10.1039/C3SM51147B.

(23) Lim, J. Y.; Hansen, J. C.; Siedlecki, C. A.; Runt, J.; Donahue, H. J. Human Foetal Osteoblastic Cell Response to Polymer-Demixed Nanotopographic Interfaces. *J R Soc Interface* **2005**, *2* (2), 97–108. https://doi.org/10.1098/RSIF.2004.0019.

(24) Bian, Q.; Wang, W.; Han, G.; Chen, Y.; Wang, S.; Wang, G. Photoswitched Cell Adhesion on Azobenzene-Containing Self-Assembled Films. *Chemphyschem* **2016**, *17* (16), 2503–2508. https://doi.org/10.1002/CPHC.201600362.

(25) Zhang, J.; Li, G.; Li, D.; Zhang, X.; Li, Q.; Liu, Z.; Fang, Y.; Zhang, S.; Man, J. In Vivo Blood-Repellent Performance of a Controllable Facile-Generated Superhydrophobic Surface. *ACS Appl Mater Interfaces* **2021**, *13* (24), 29021–29033. https://doi.org/10.1021/ACSAMI.0C21058/ASSET/IMAGES/LARGE/AM0C21058_0008.JPEG.

(26) De Vitis, S.; Coluccio, M. L.; Gentile, F.; Malara, N.; Perozziello, G.; Dattola, E.; Candeloro, P.; Di Fabrizio, E. Surface Enhanced Raman Spectroscopy Measurements of MCF7 Cells Adhesion in Confined Micro-Environments. *Opt Lasers Eng* **2016**, *76*, 9–16. https://doi.org/10.1016/J.OPTLASENG.2015.04.010.

(27) Accardo, A.; Shalabaeva, V.; La Rocca, R. Colon Cancer Cells Adhesion on Polymeric Nanostructured Surfaces. *MRS Commun* **2018**, *8* (1), 35–39. https://doi.org/10.1557/MRC.2017.128.

(28) Ghalandarzadeh, A.; Ganjali, M.; Hosseini, M. Effects of Surface Topography through Laser Texturing on the Surface Characteristics of Zirconia-Based Dental Materials: Surface Hydrophobicity, Antibacterial Behavior, and Cellular Response. *Surf Topogr* **2023**, *11* (2), 025007. https://doi.org/10.1088/2051-672X/ACD076.

(29) Zhou, J.; Fan, J. B.; Nie, Q.; Wang, S. Three-Dimensional Superhydrophobic Copper 7,7,8,8-Tetracyanoquinodimethane Biointerfaces with the Capability of High Adhesion of Osteoblasts. *Nanoscale* **2016**, *8* (6), 3264–3267. https://doi.org/10.1039/C5NR08305B.

(30) Falde, E. J.; Yohe, S. T.; Colson, Y. L.; Grinstaff, M. W. Superhydrophobic Materials for Biomedical Applications. *Biomaterials*. Elsevier Ltd October 1, 2016, pp 87–103. https://doi.org/10.1016/j.biomaterials.2016.06.050.

(31) Cui, H.; Wang, W.; Shi, L.; Song, W.; Wang, S. Superwettable Surface Engineering in Controlling Cell Adhesion for Emerging Bioapplications. *Small Methods*. John Wiley and Sons Inc December 1, 2020. https://doi.org/10.1002/smtd.202000573.

(32) Luo, J.; Yu, H.; Lu, B.; Wang, D.; Deng, X. Superhydrophobic Biological Fluid-Repellent Surfaces: Mechanisms and Applications. *Small Methods*. John Wiley and Sons Inc December 15, 2022. https://doi.org/10.1002/smtd.202201106.

(33) Ferrari, M.; Cirisano, F.; Carmen Morán, M. Mammalian Cell Behavior on Hydrophobic Substrates: Influence of Surface Properties. *Colloids and Interfaces*. MDPI AG 2019. https://doi.org/10.3390/colloids3020048.

(34) Haynes, J.; Srivastava, J.; Madson, N.; Wittmann, T.; Barber, D. L. Dynamic Actin Remodeling during Epithelial–Mesenchymal Transition Depends on Increased Moesin

Expression. *Mol Biol Cell* **2011**, *22* (24), 4750. https://doi.org/10.1091/MBC.E11-02-0119.

(35) Zuchowska, A.; Kwiatkowski, P.; Jastrzebska, E.; Chudy, M.; Dybko, A.; Brzozka, Z. Adhesion of MRC-5 and A549 Cells on Poly(Dimethylsiloxane) Surface Modified by Proteins. *Electrophoresis* **2016**, *37* (3), 536–544. https://doi.org/10.1002/ELPS.201500250.

(36) Günzel, D.; Yu, A. S. L. Claudins and the Modulation of Tight Junction Permeability. *Physiol Rev* **2013**, *93* (2), 525. https://doi.org/10.1152/PHYSREV.00019.2012.
